# Supplementary figures and images for: SRC and ERK Regulate the Turnover of Cytoskeletal Keratin Filaments
Source: Int J Mol Sci. 2025 Jun 7;26(12):5476. doi: 10.3390/ijms26125476 (PMC12193469; doi:10.3390/ijms26125476)

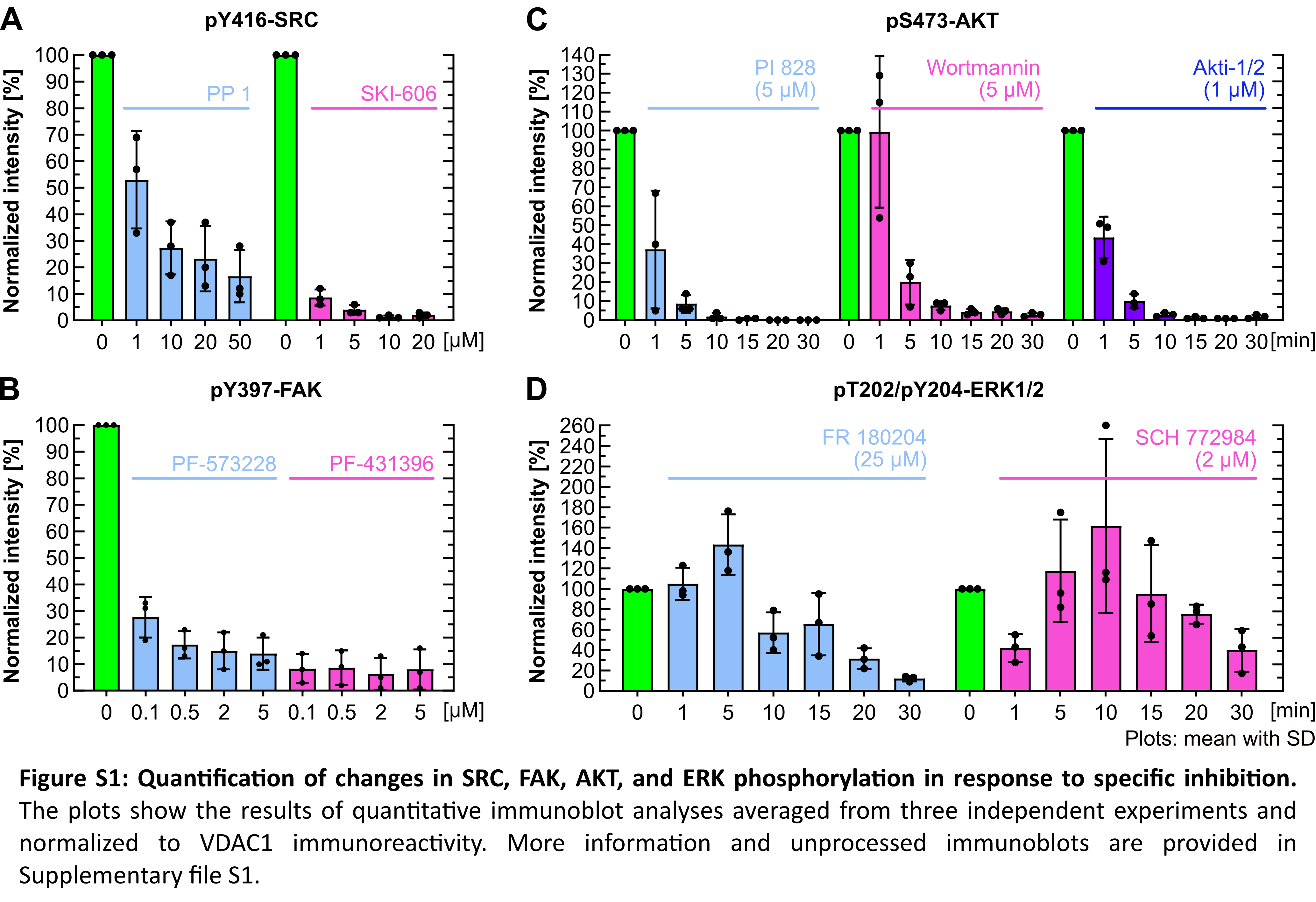

Supplement: Supplementary file 1 [file ijms-26-05476-s001.zip › Figure S1.png]

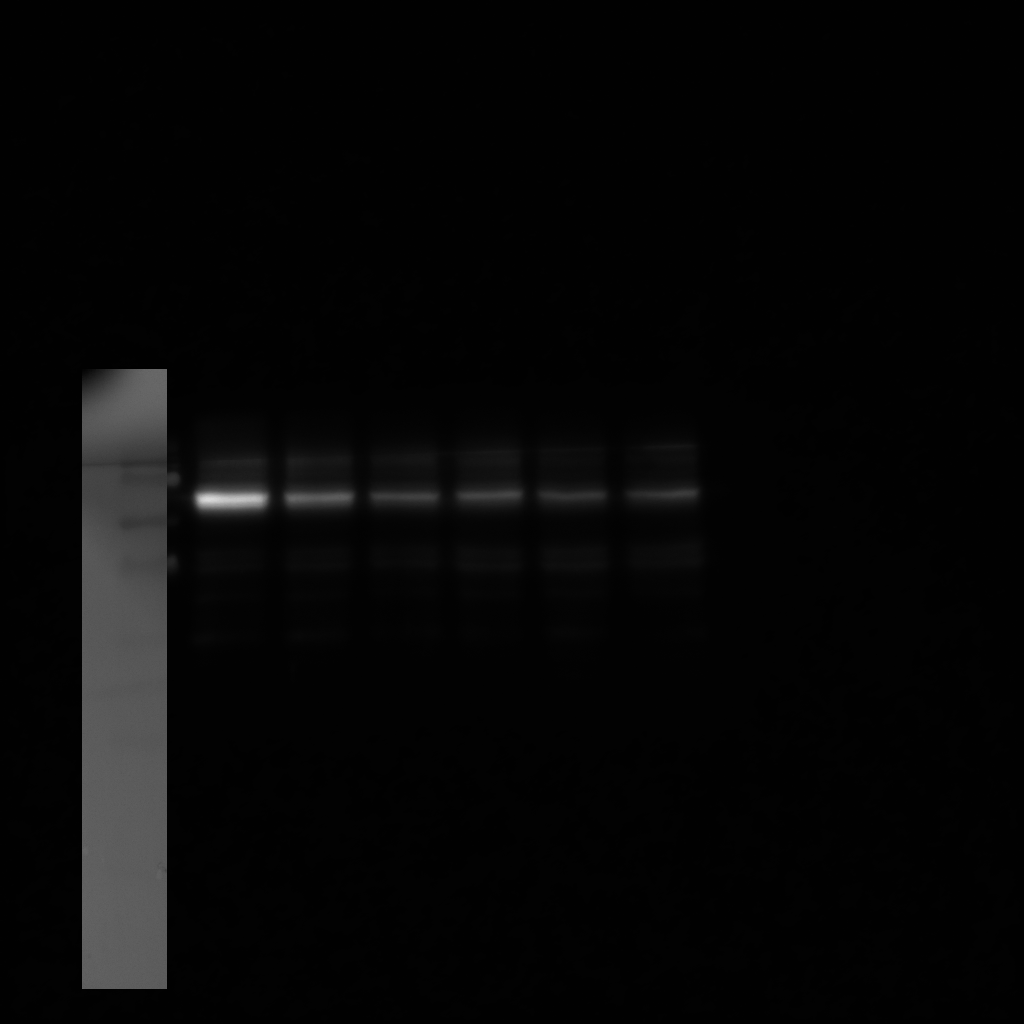

Supplement: Supplementary file 1 [file ijms-26-05476-s001.zip › Supplementary materials S1 Unprocessed immunoblots/Fig.1_SRC (PP 1)/Figure 1B_241128_1_blot1_PP1_1.0_pSrc (rb185).Tif]

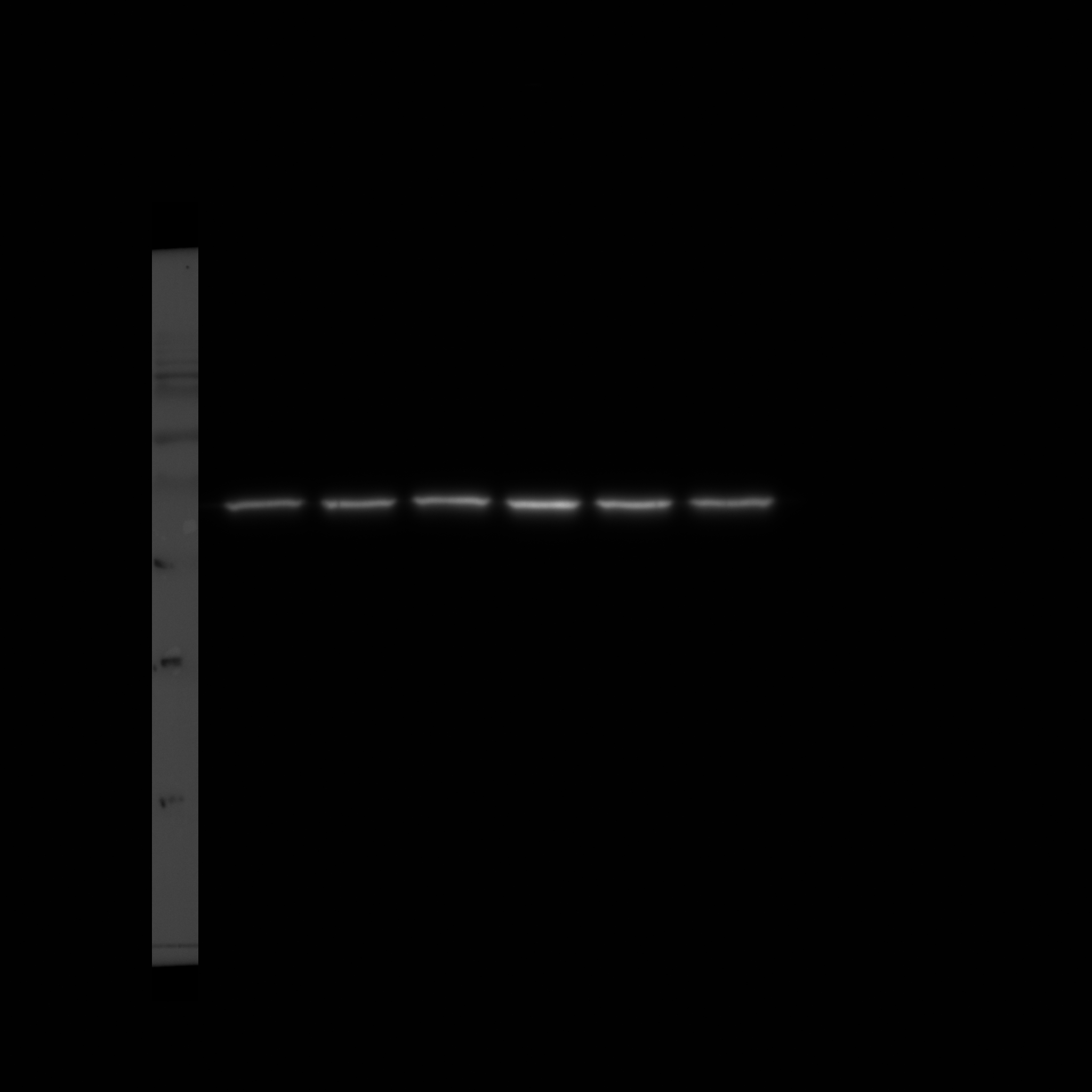

Supplement: Supplementary file 1 [file ijms-26-05476-s001.zip › Supplementary materials S1 Unprocessed immunoblots/Fig.1_SRC (PP 1)/Figure 1B_241128_2_blot1_PP1_1.0_VDAC1 (rb201).Tif]

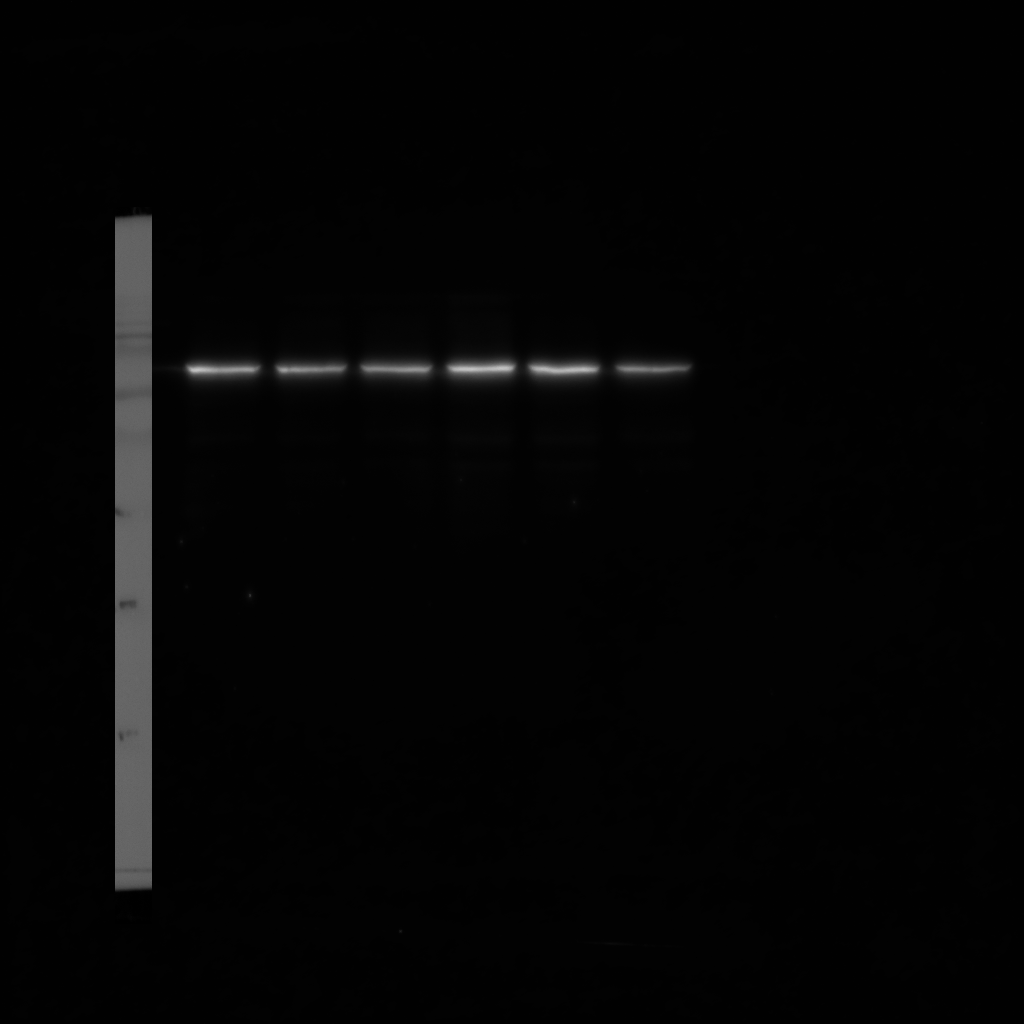

Supplement: Supplementary file 1 [file ijms-26-05476-s001.zip › Supplementary materials S1 Unprocessed immunoblots/Fig.1_SRC (PP 1)/Figure 1B_241128_3_blot1_PP1_1.0_Src (mc241).Tif]

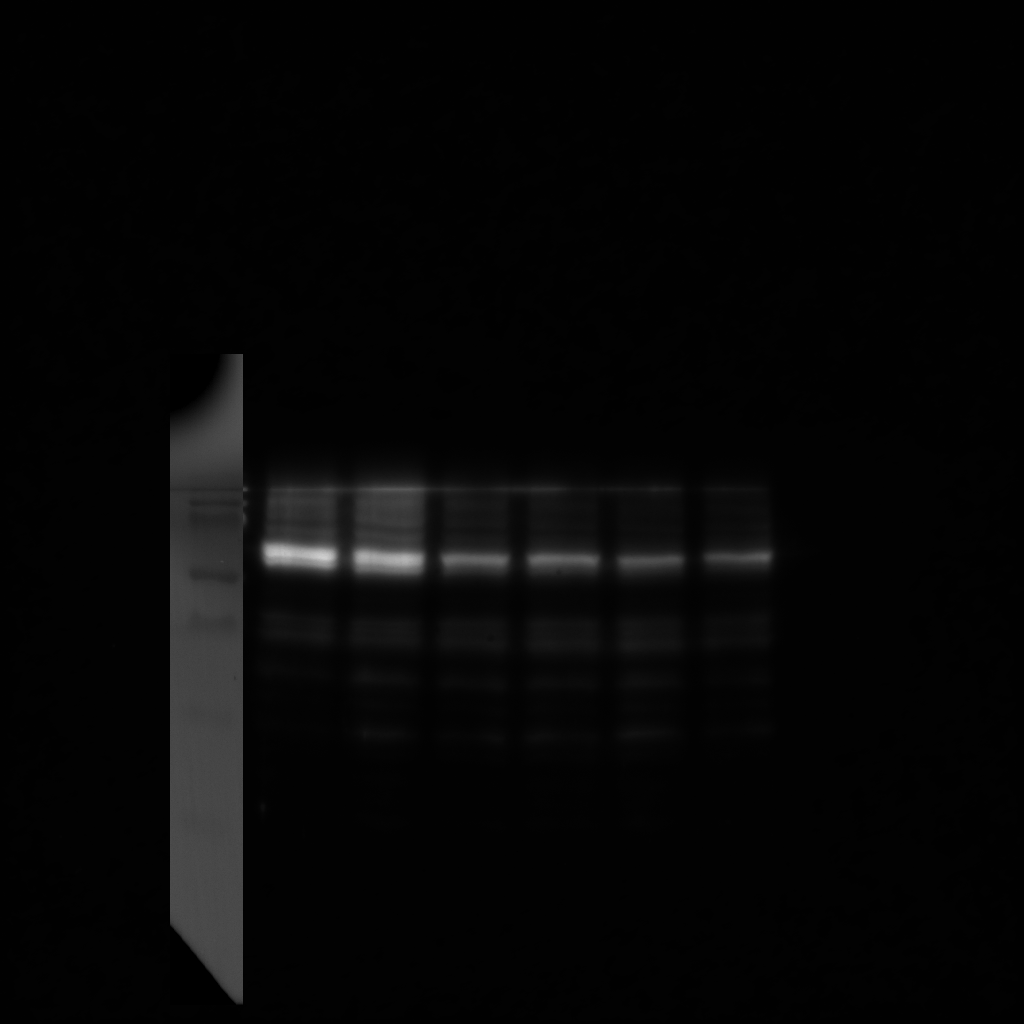

Supplement: Supplementary file 1 [file ijms-26-05476-s001.zip › Supplementary materials S1 Unprocessed immunoblots/Fig.1_SRC (PP 1)/not shown_241128_1b_blot3_PP1_1.2_pSrc (rb185).Tif]

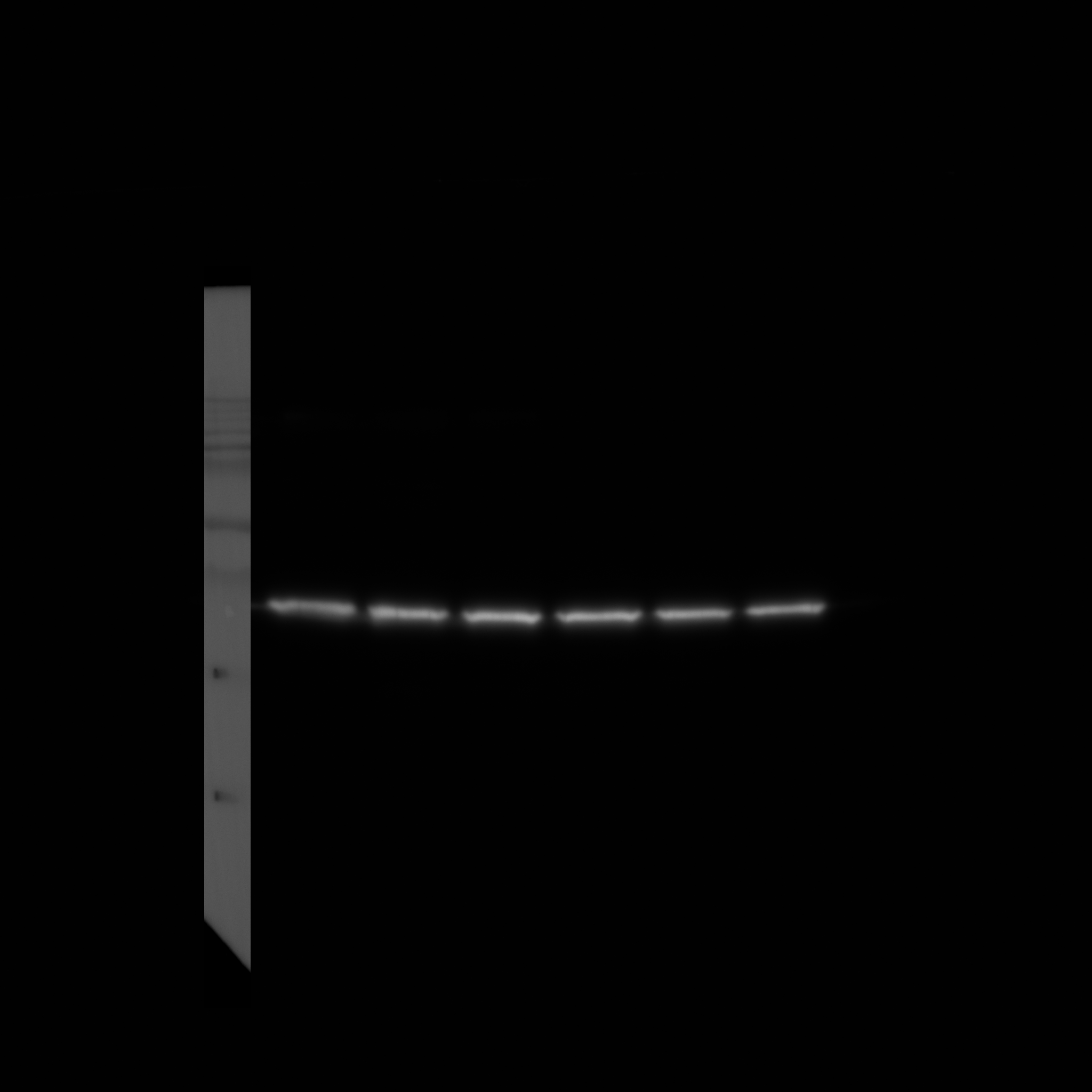

Supplement: Supplementary file 1 [file ijms-26-05476-s001.zip › Supplementary materials S1 Unprocessed immunoblots/Fig.1_SRC (PP 1)/not shown_241128_2_blot3_PP1_1.2_VDAC1 (rb201).Tif]

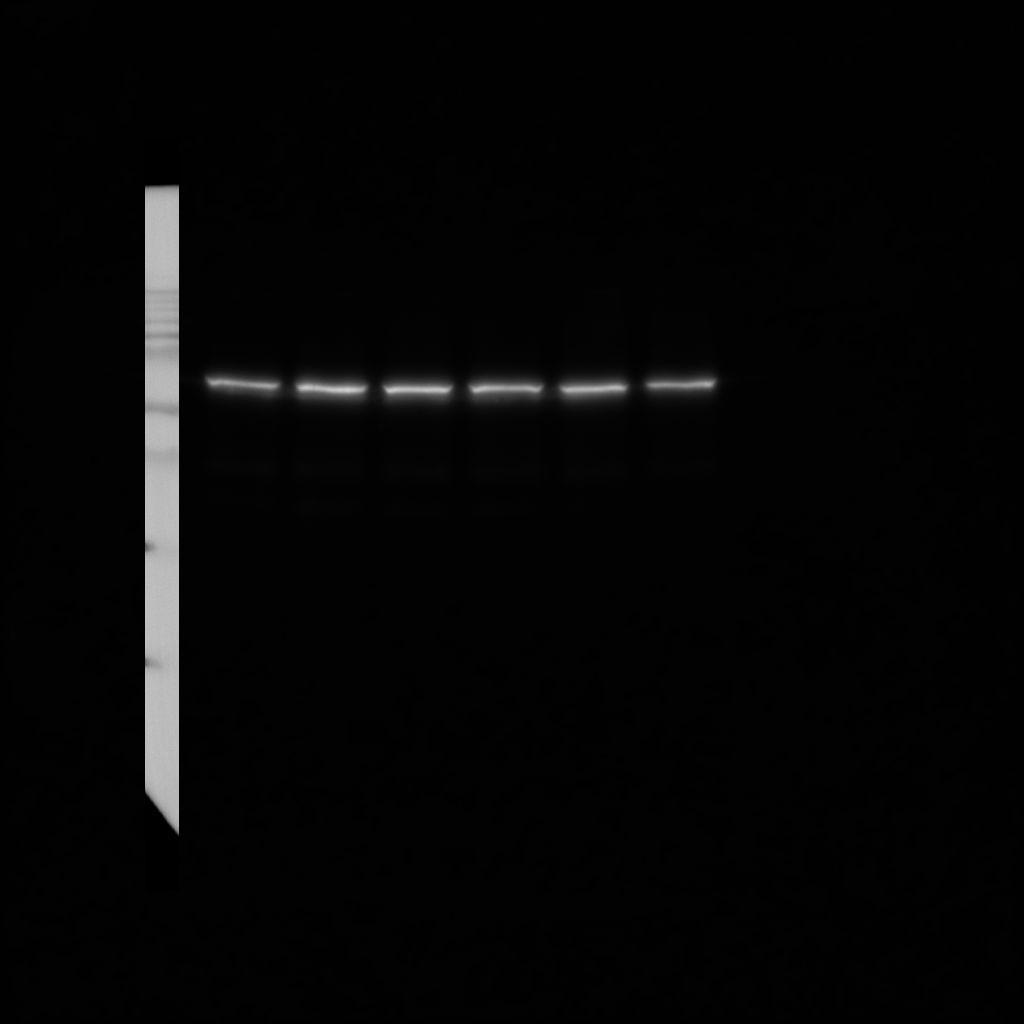

Supplement: Supplementary file 1 [file ijms-26-05476-s001.zip › Supplementary materials S1 Unprocessed immunoblots/Fig.1_SRC (PP 1)/not shown_241128_3_blot3_PP1_1.2_Src (mc241).Tif]

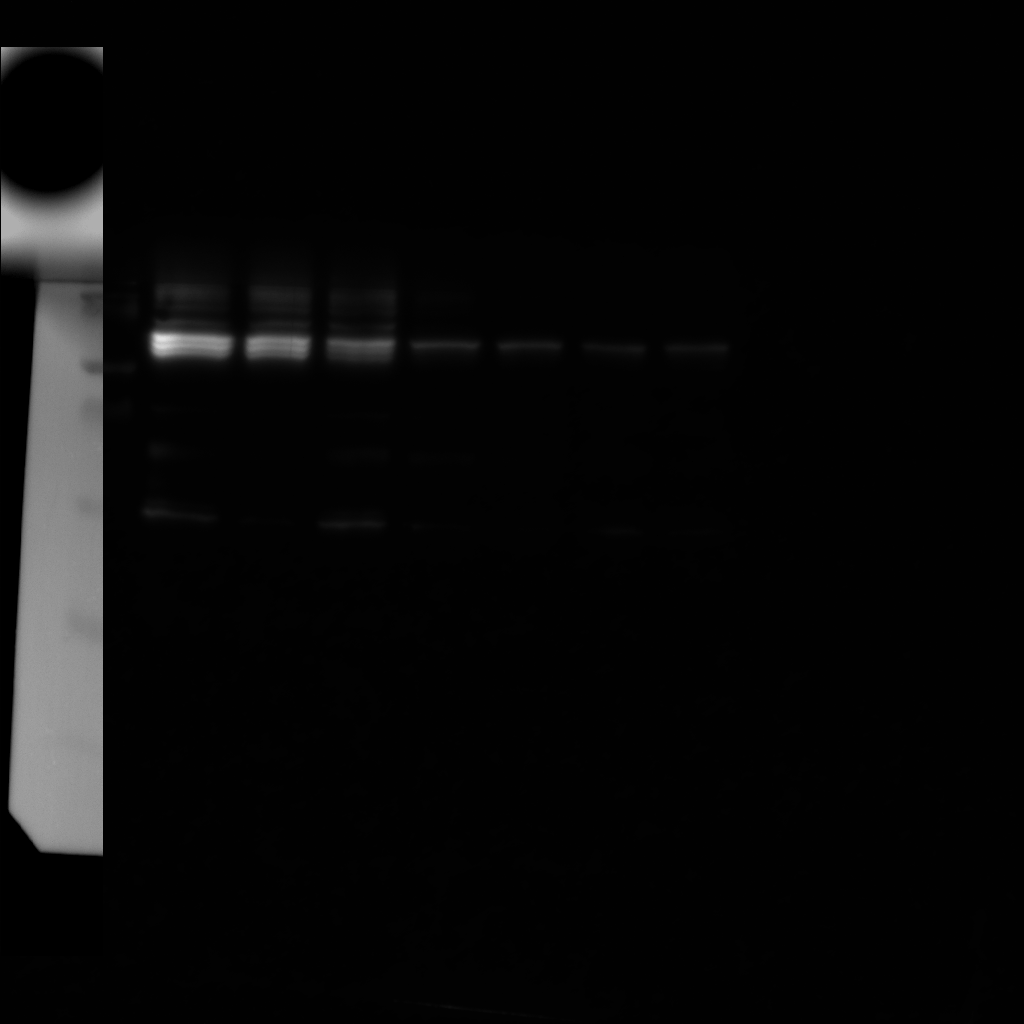

Supplement: Supplementary file 1 [file ijms-26-05476-s001.zip › Supplementary materials S1 Unprocessed immunoblots/Fig.1_SRC (PP 1)/not shown_241219_1_AK13-1_PP1_1.1_redone_pSrc (rb185).Tif]

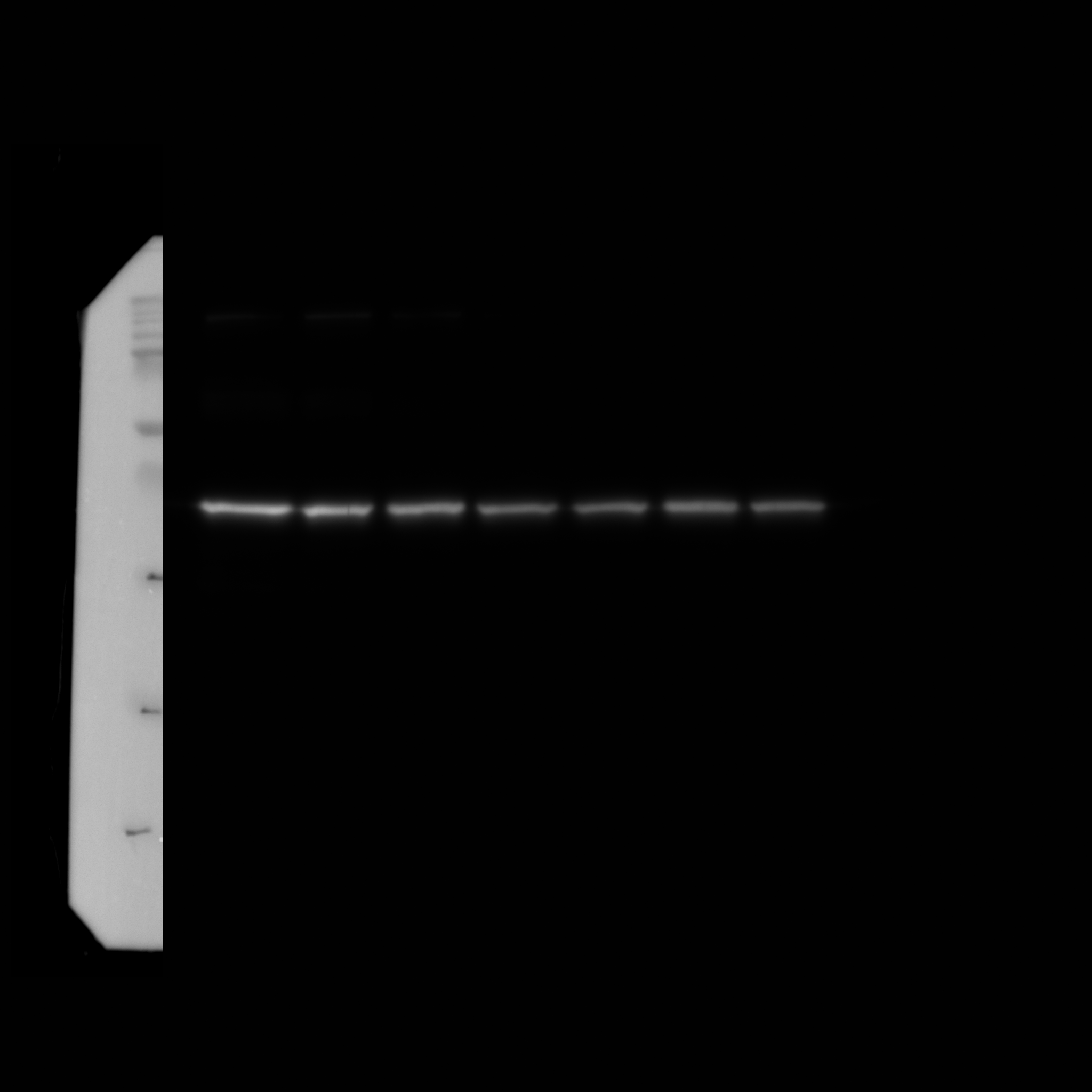

Supplement: Supplementary file 1 [file ijms-26-05476-s001.zip › Supplementary materials S1 Unprocessed immunoblots/Fig.1_SRC (PP 1)/not shown_241219_2_AK13-1_PP1_1.1_redone_VDAC1 (rb201).Tif]

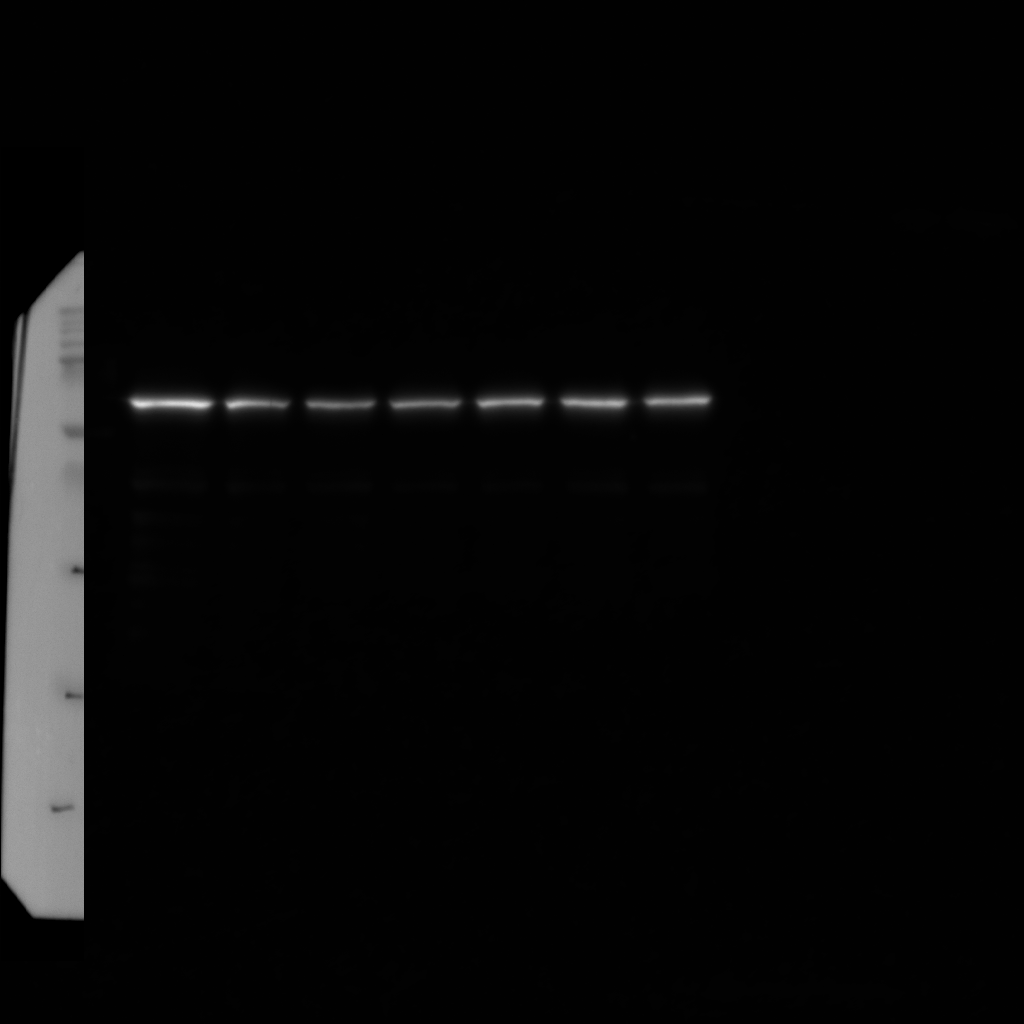

Supplement: Supplementary file 1 [file ijms-26-05476-s001.zip › Supplementary materials S1 Unprocessed immunoblots/Fig.1_SRC (PP 1)/not shown_241219_3_AK13-1_PP1_1.1_redone_Src (mc241).Tif]

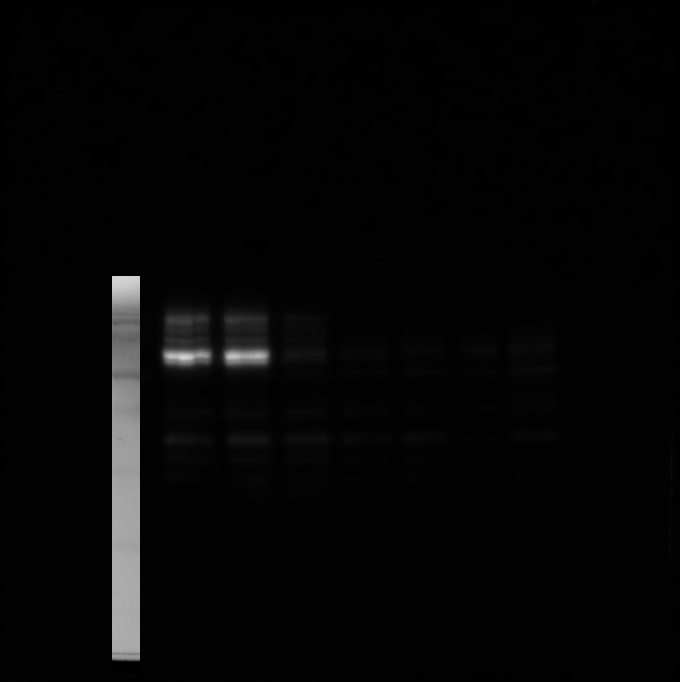

Supplement: Supplementary file 1 [file ijms-26-05476-s001.zip › Supplementary materials S1 Unprocessed immunoblots/Fig.1_SRC (SKI-606)/Figure 1B_241202_2b_blot3_SKI-606_v.1.2_pSrc (rb185).Tif]

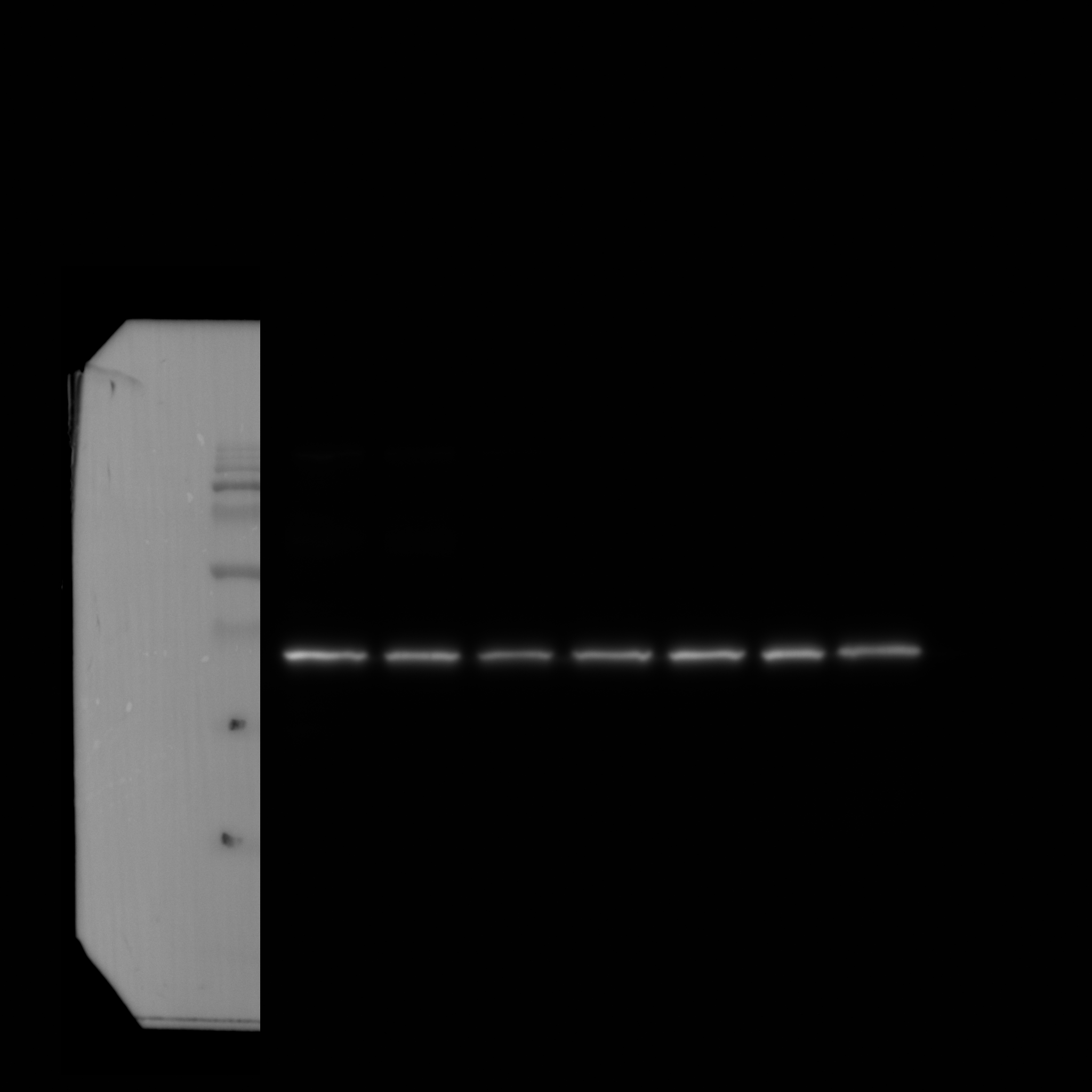

Supplement: Supplementary file 1 [file ijms-26-05476-s001.zip › Supplementary materials S1 Unprocessed immunoblots/Fig.1_SRC (SKI-606)/Figure 1B_241202_3_blot3_SKI-606_v.1.2_VDAC1 (rb201).Tif]

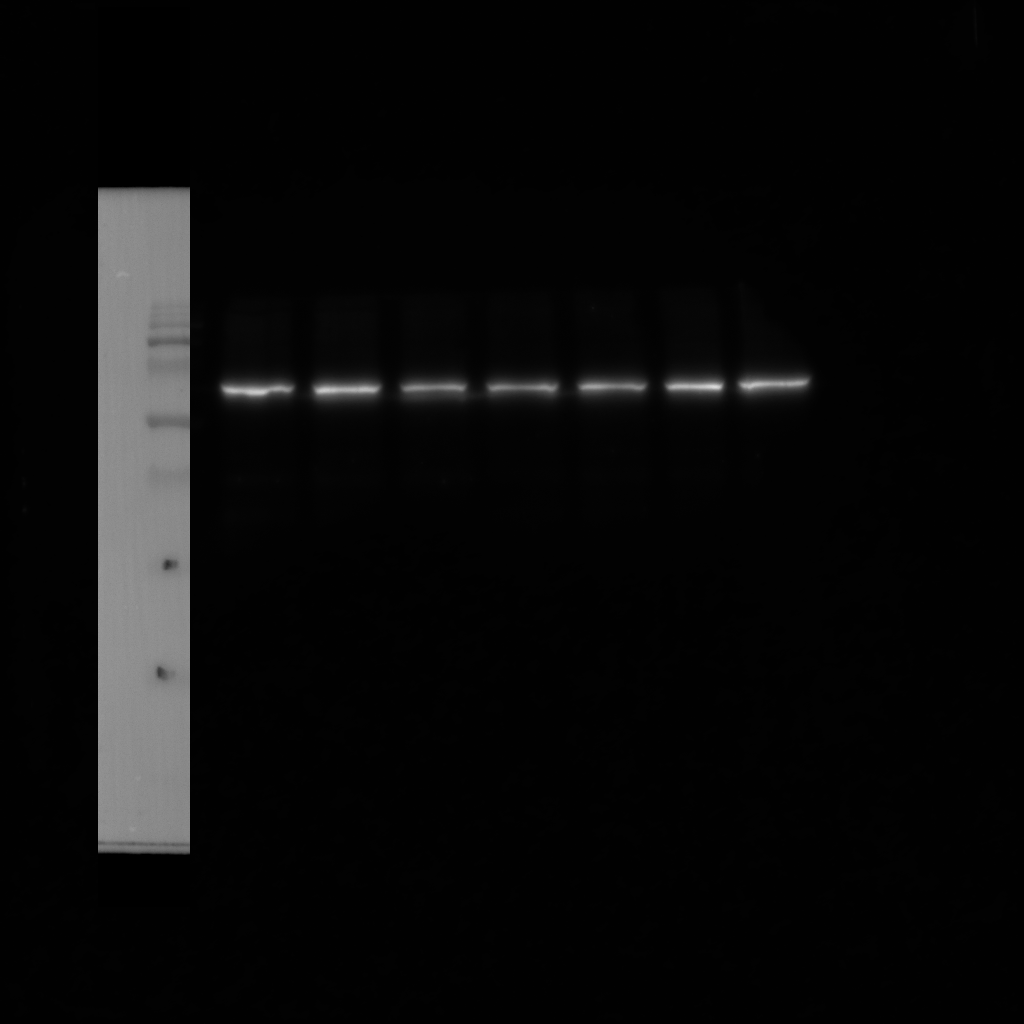

Supplement: Supplementary file 1 [file ijms-26-05476-s001.zip › Supplementary materials S1 Unprocessed immunoblots/Fig.1_SRC (SKI-606)/Figure 1B_241202_4_blot3_SKI-606_v.1.2_Src (mc241).Tif]

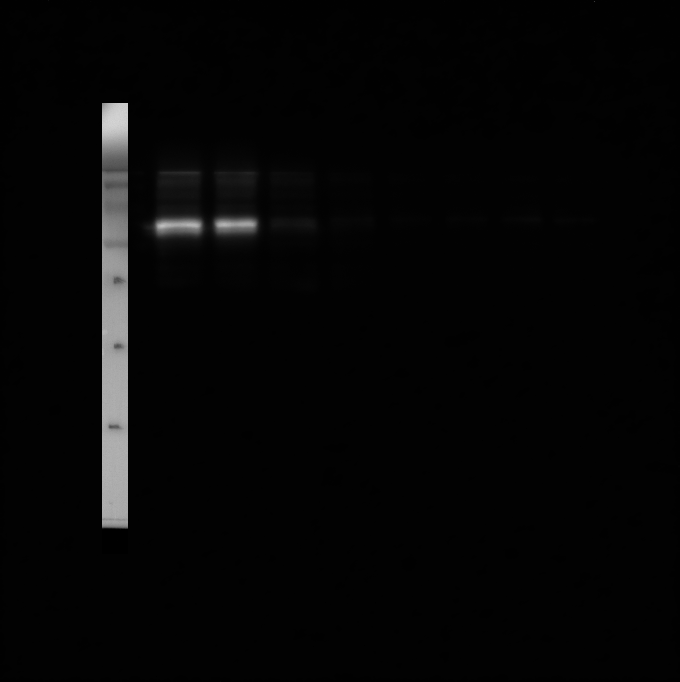

Supplement: Supplementary file 1 [file ijms-26-05476-s001.zip › Supplementary materials S1 Unprocessed immunoblots/Fig.1_SRC (SKI-606)/not shown_241202_2b_blot1_SKI-606_v.1.0_pSrc (rb185).Tif]

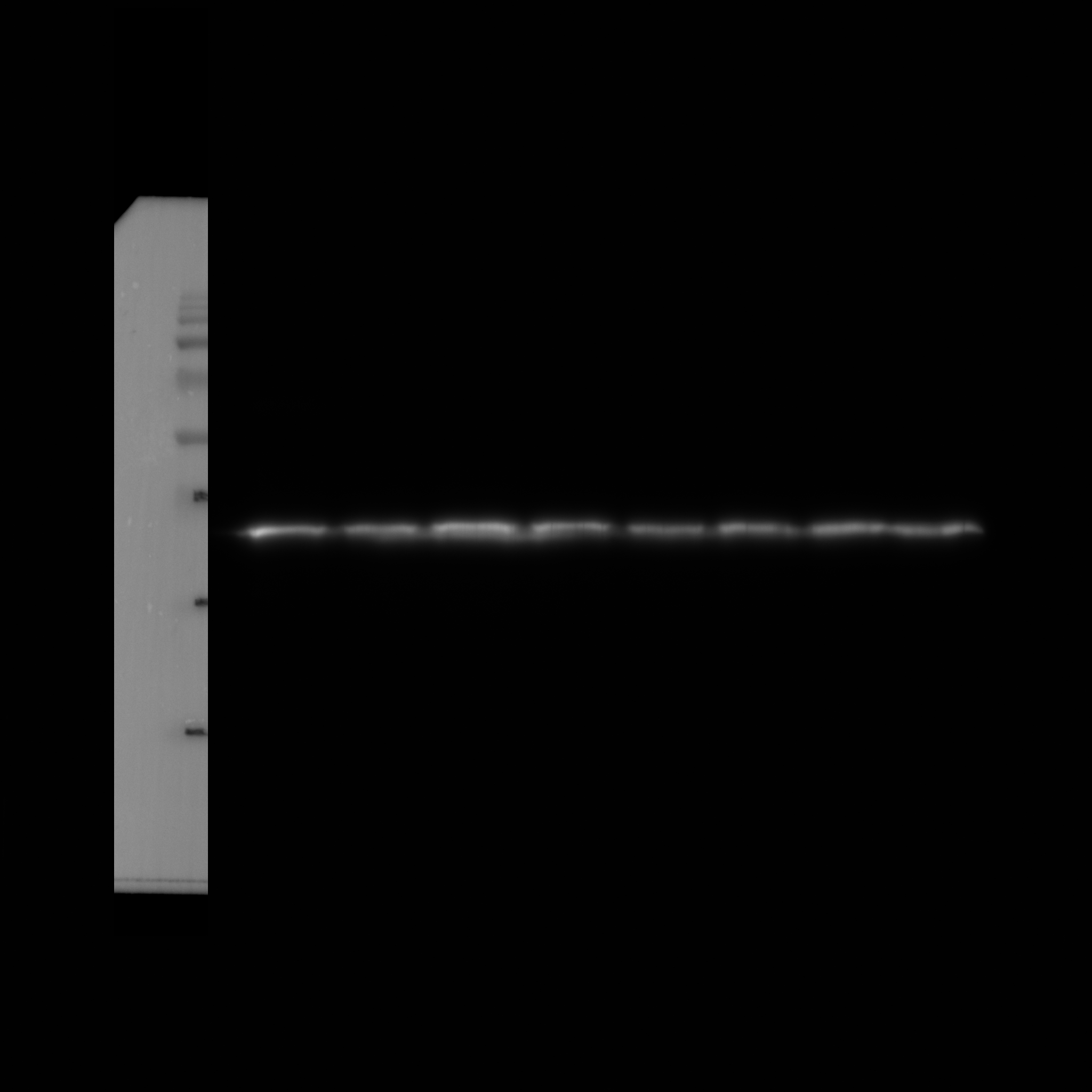

Supplement: Supplementary file 1 [file ijms-26-05476-s001.zip › Supplementary materials S1 Unprocessed immunoblots/Fig.1_SRC (SKI-606)/not shown_241202_3_blot1_SKI-606_v.1.0_VDAC1 (rb201).Tif]

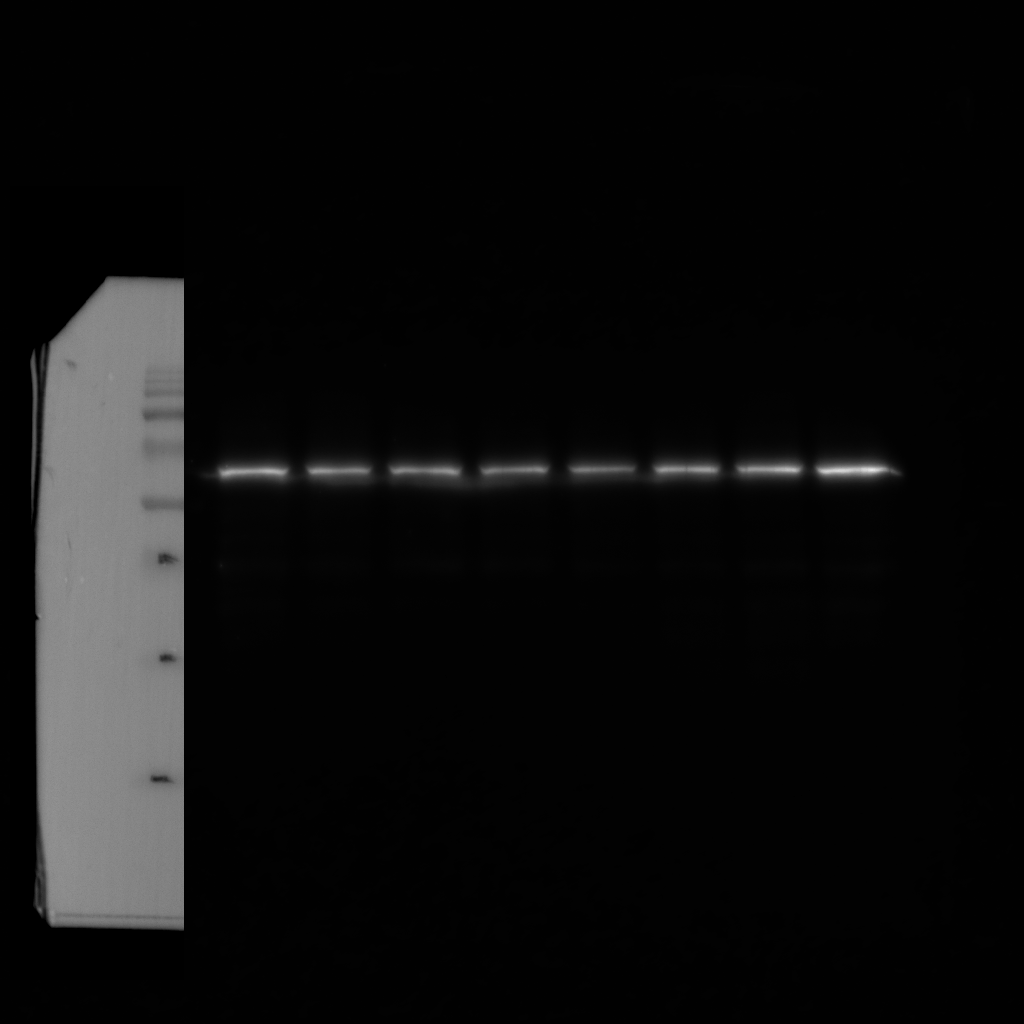

Supplement: Supplementary file 1 [file ijms-26-05476-s001.zip › Supplementary materials S1 Unprocessed immunoblots/Fig.1_SRC (SKI-606)/not shown_241202_4_blot1_SKI-606_v.1.0_Src (mc241).Tif]

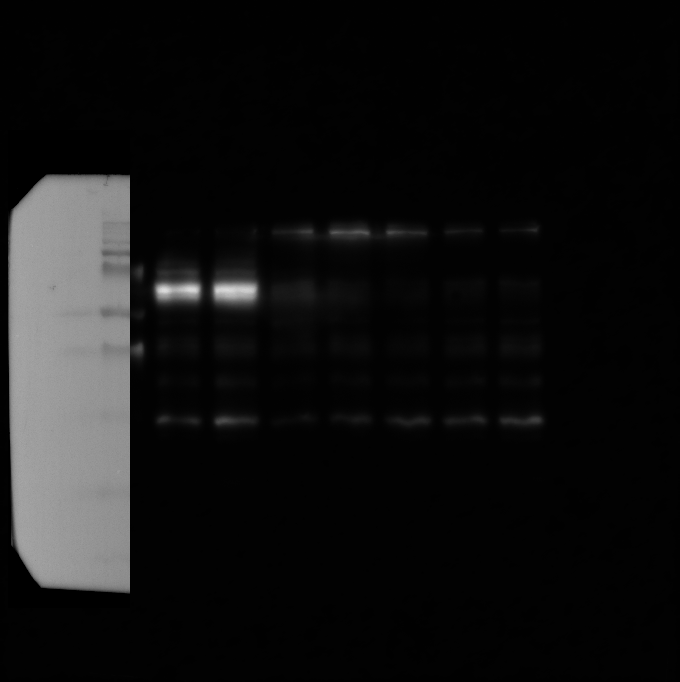

Supplement: Supplementary file 1 [file ijms-26-05476-s001.zip › Supplementary materials S1 Unprocessed immunoblots/Fig.1_SRC (SKI-606)/not shown_241215_1_blot2_SKI-606_v.1.1_pSrc (rb185).Tif]

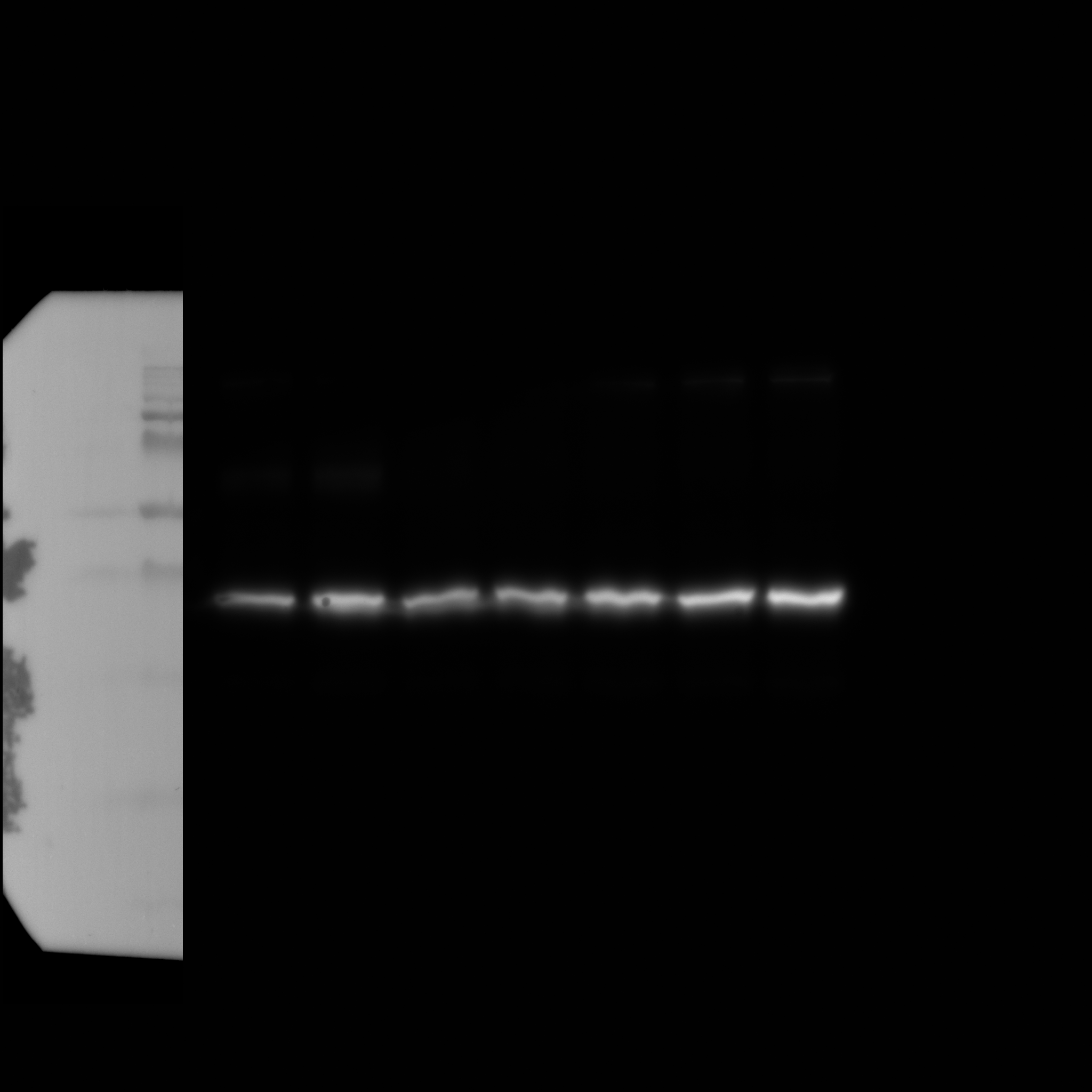

Supplement: Supplementary file 1 [file ijms-26-05476-s001.zip › Supplementary materials S1 Unprocessed immunoblots/Fig.1_SRC (SKI-606)/not shown_241215_2_blot2_SKI-606_v.1.1_VDAC1 (rb201).Tif]

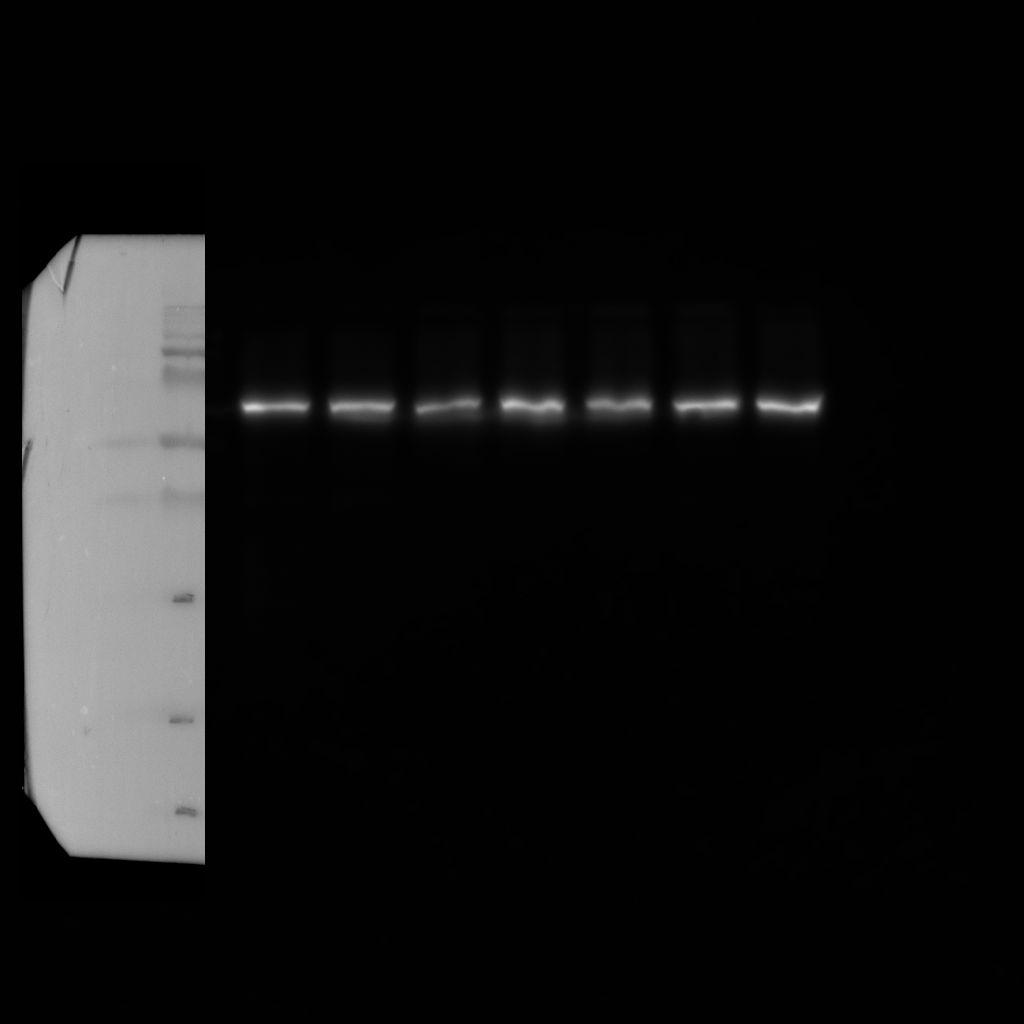

Supplement: Supplementary file 1 [file ijms-26-05476-s001.zip › Supplementary materials S1 Unprocessed immunoblots/Fig.1_SRC (SKI-606)/not shown_241215_5_blot2_SKI-606_v.1.1_Src (mc241).Tif]

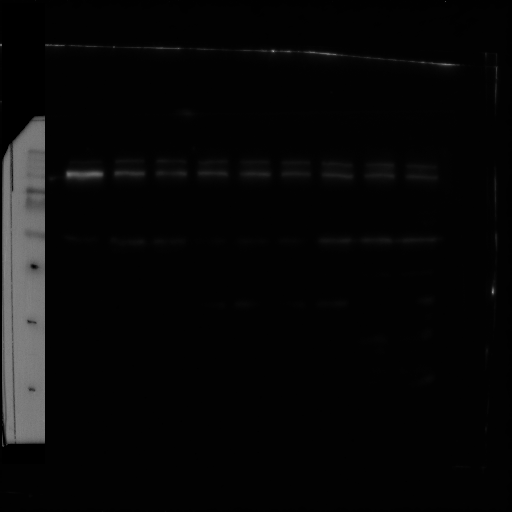

Supplement: Supplementary file 1 [file ijms-26-05476-s001.zip › Supplementary materials S1 Unprocessed immunoblots/Fig.2_FAK (PF-228, PF-396)/Figure 2A_2024-12-06_1_blot1_PF-228_PF-396_v1.0_pFAK (rb152).Tif]

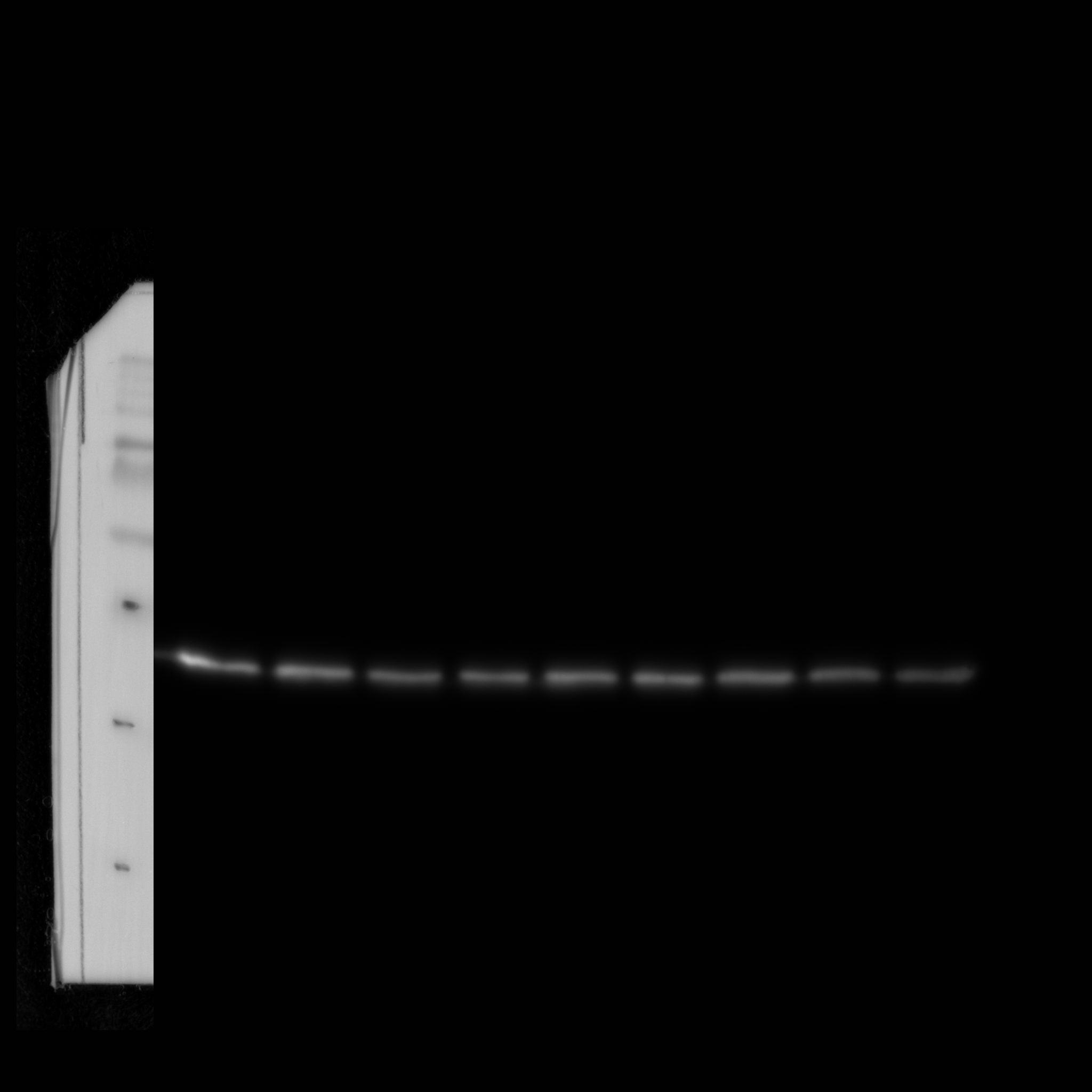

Supplement: Supplementary file 1 [file ijms-26-05476-s001.zip › Supplementary materials S1 Unprocessed immunoblots/Fig.2_FAK (PF-228, PF-396)/Figure 2A_2024-12-06_2_blot1_PF-228_PF-396_v1.0_VDAC1 (rb201).Tif]

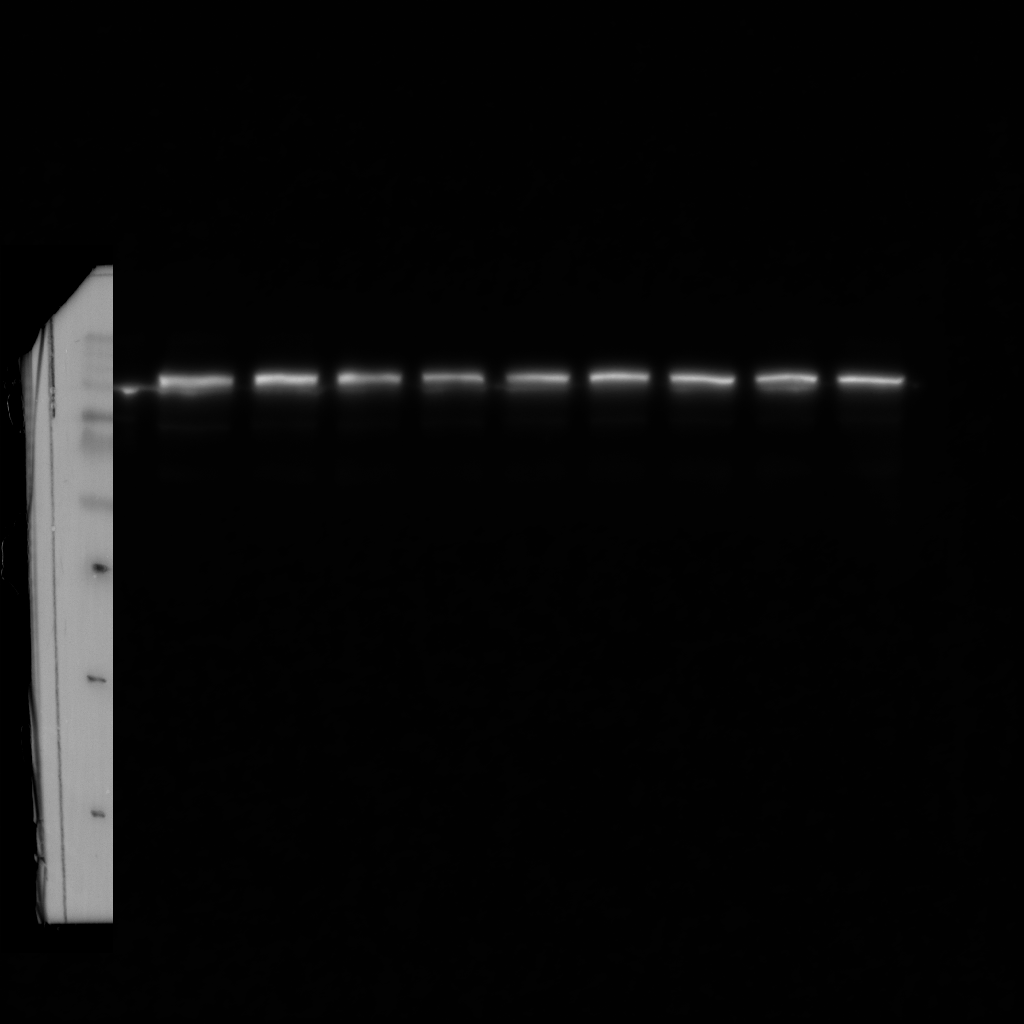

Supplement: Supplementary file 1 [file ijms-26-05476-s001.zip › Supplementary materials S1 Unprocessed immunoblots/Fig.2_FAK (PF-228, PF-396)/Figure 2A_2024-12-06_3_blot1_PF-228_PF-396_v1.0_mc111.Tif]

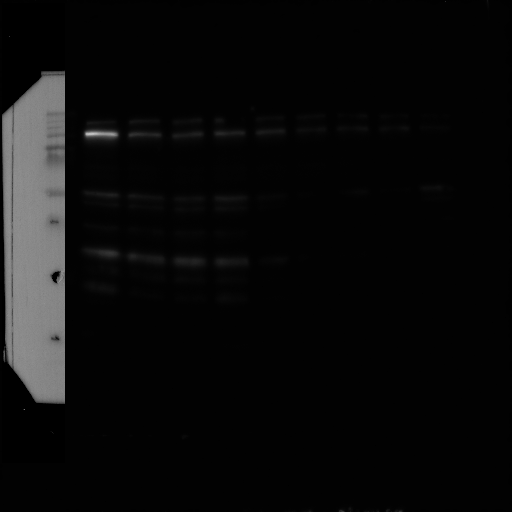

Supplement: Supplementary file 1 [file ijms-26-05476-s001.zip › Supplementary materials S1 Unprocessed immunoblots/Fig.2_FAK (PF-228, PF-396)/not shown_2024-12-06_1_blot2_PF-228_PF-396_v1.1_pFAK (rb152).Tif]

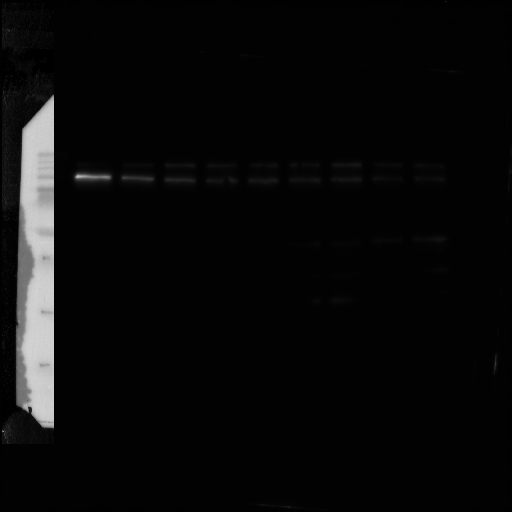

Supplement: Supplementary file 1 [file ijms-26-05476-s001.zip › Supplementary materials S1 Unprocessed immunoblots/Fig.2_FAK (PF-228, PF-396)/not shown_2024-12-06_1_blot3_PF-228_PF-396_v1.2_pFAK (rb152).Tif]

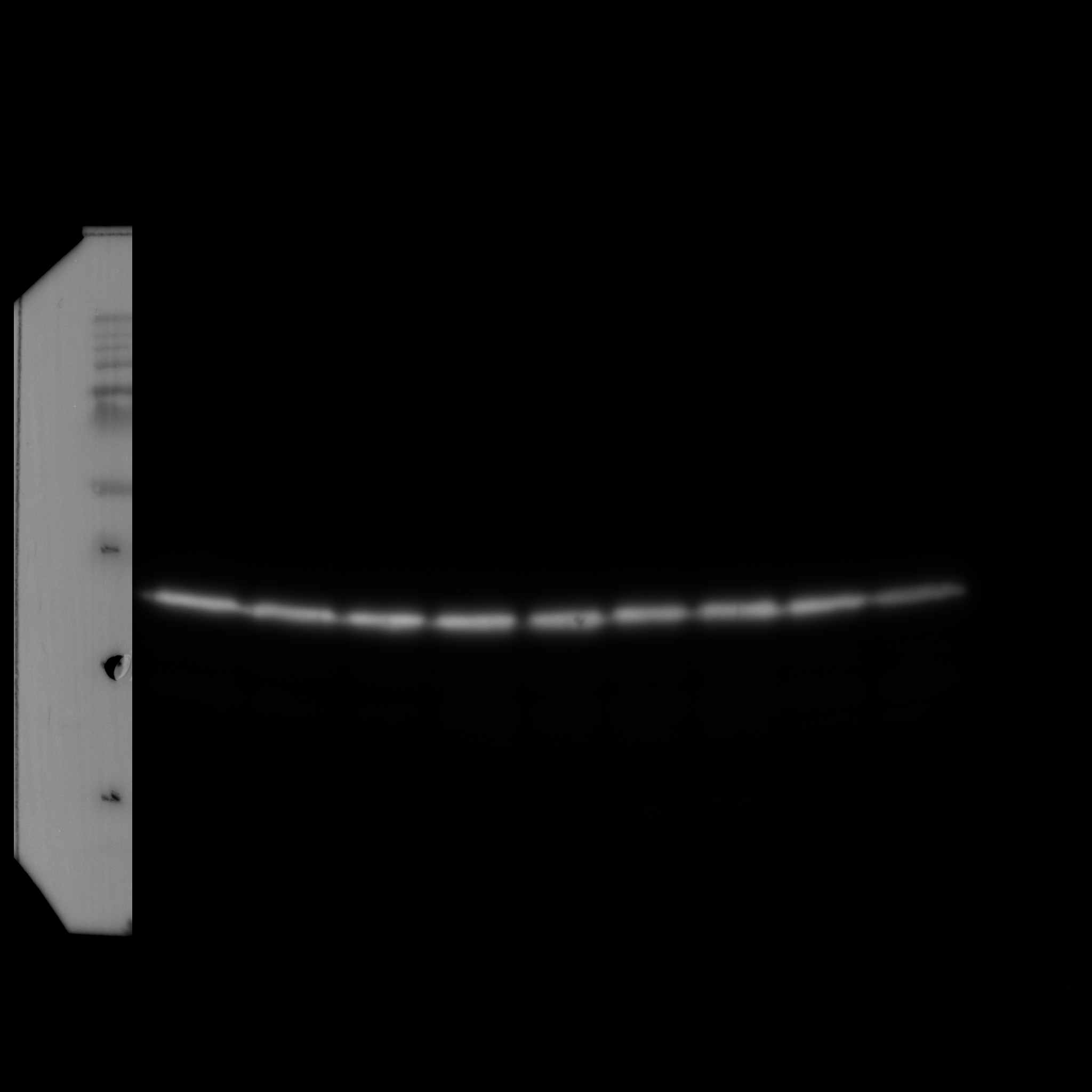

Supplement: Supplementary file 1 [file ijms-26-05476-s001.zip › Supplementary materials S1 Unprocessed immunoblots/Fig.2_FAK (PF-228, PF-396)/not shown_2024-12-06_2_blot2_PF-228_PF-396_v1.1_VDAC1 (rb201).Tif]

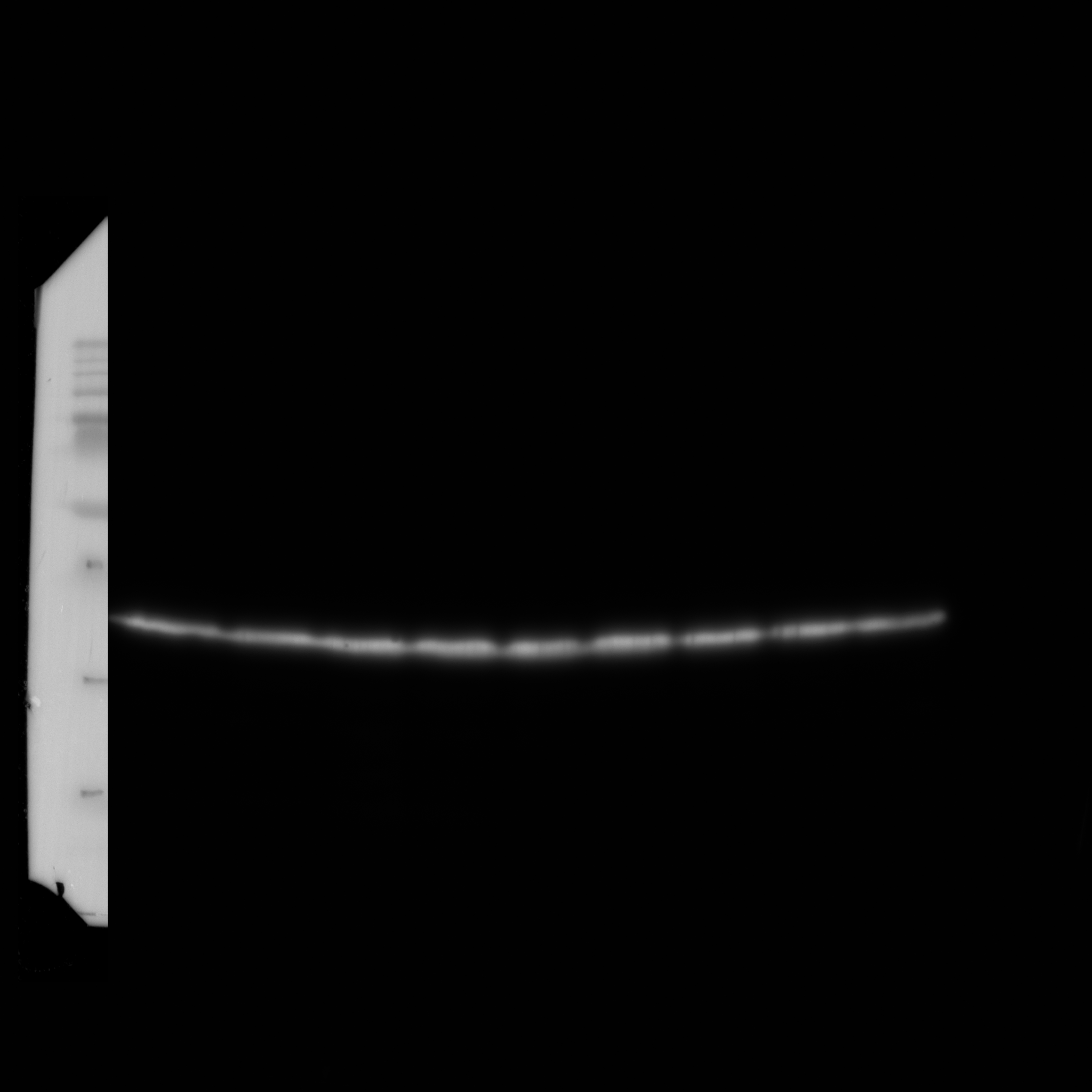

Supplement: Supplementary file 1 [file ijms-26-05476-s001.zip › Supplementary materials S1 Unprocessed immunoblots/Fig.2_FAK (PF-228, PF-396)/not shown_2024-12-06_2_blot3_PF-228_PF-396_v1.2_VDAC1 (rb201).Tif]

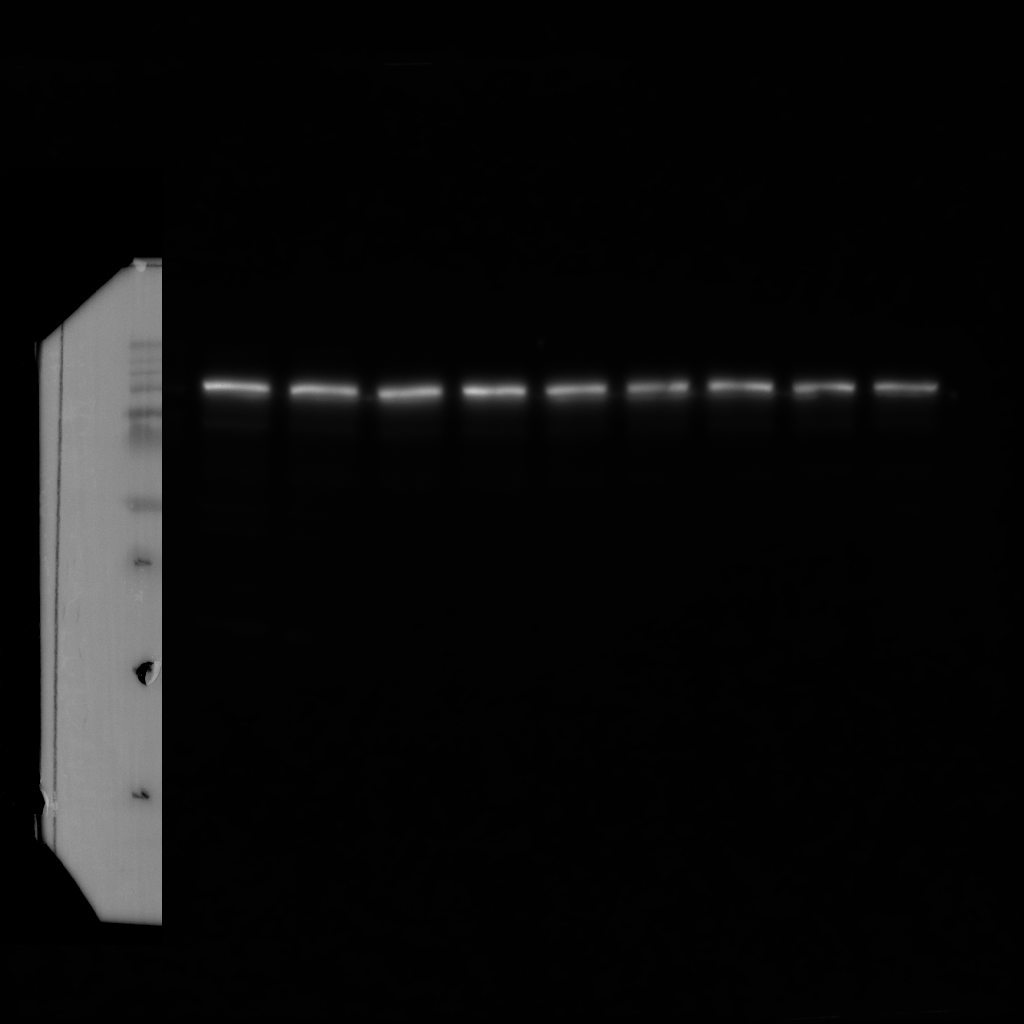

Supplement: Supplementary file 1 [file ijms-26-05476-s001.zip › Supplementary materials S1 Unprocessed immunoblots/Fig.2_FAK (PF-228, PF-396)/not shown_2024-12-06_3_blot2_PF-228_PF-396_v1.1_mc111.Tif]

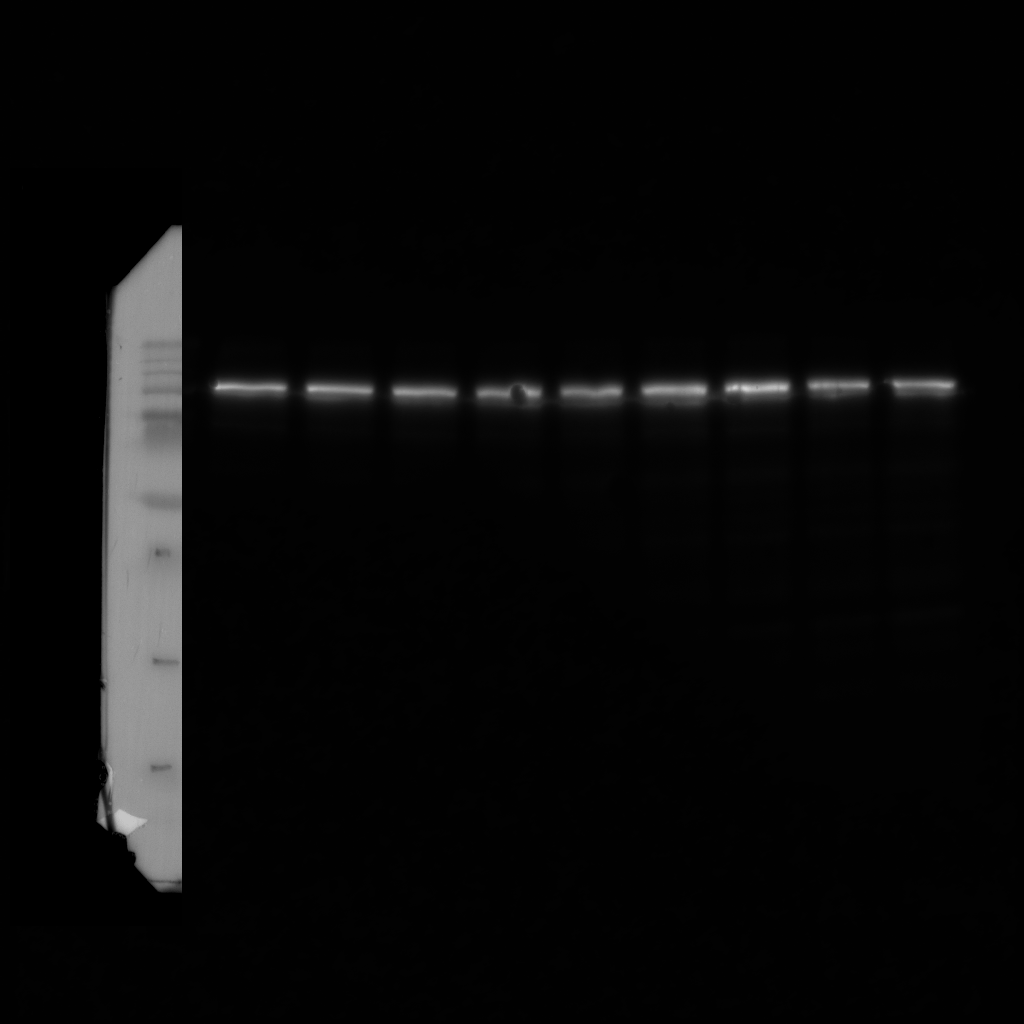

Supplement: Supplementary file 1 [file ijms-26-05476-s001.zip › Supplementary materials S1 Unprocessed immunoblots/Fig.2_FAK (PF-228, PF-396)/not shown_2024-12-06_3_blot3_PF-228_PF-396_v1.2_mc111.Tif]

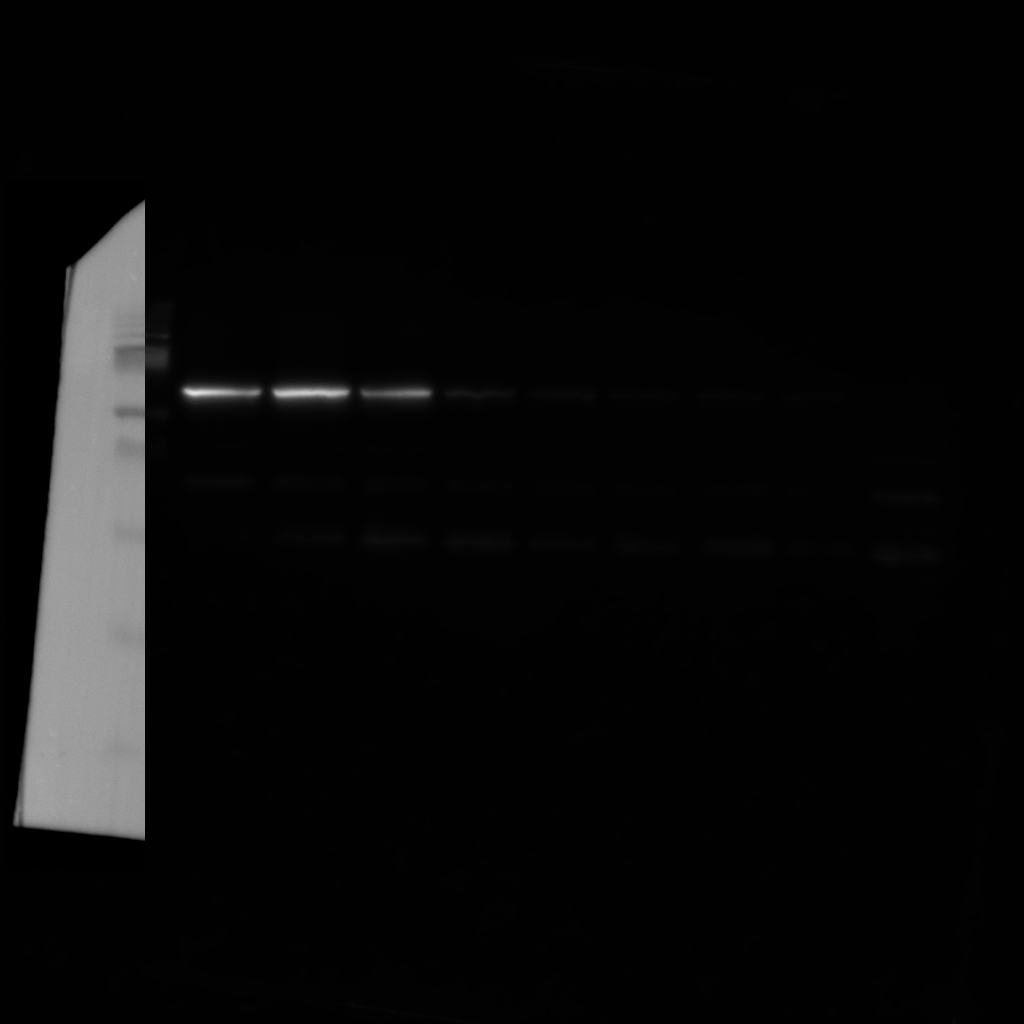

Supplement: Supplementary file 1 [file ijms-26-05476-s001.zip › Supplementary materials S1 Unprocessed immunoblots/Fig.3_AKT (Akti)/Figure 3C_241219_1_blot1_AK13-1_0%FCS_Akti_1uM_time_v.1.0_pAkt (rb362).Tif]

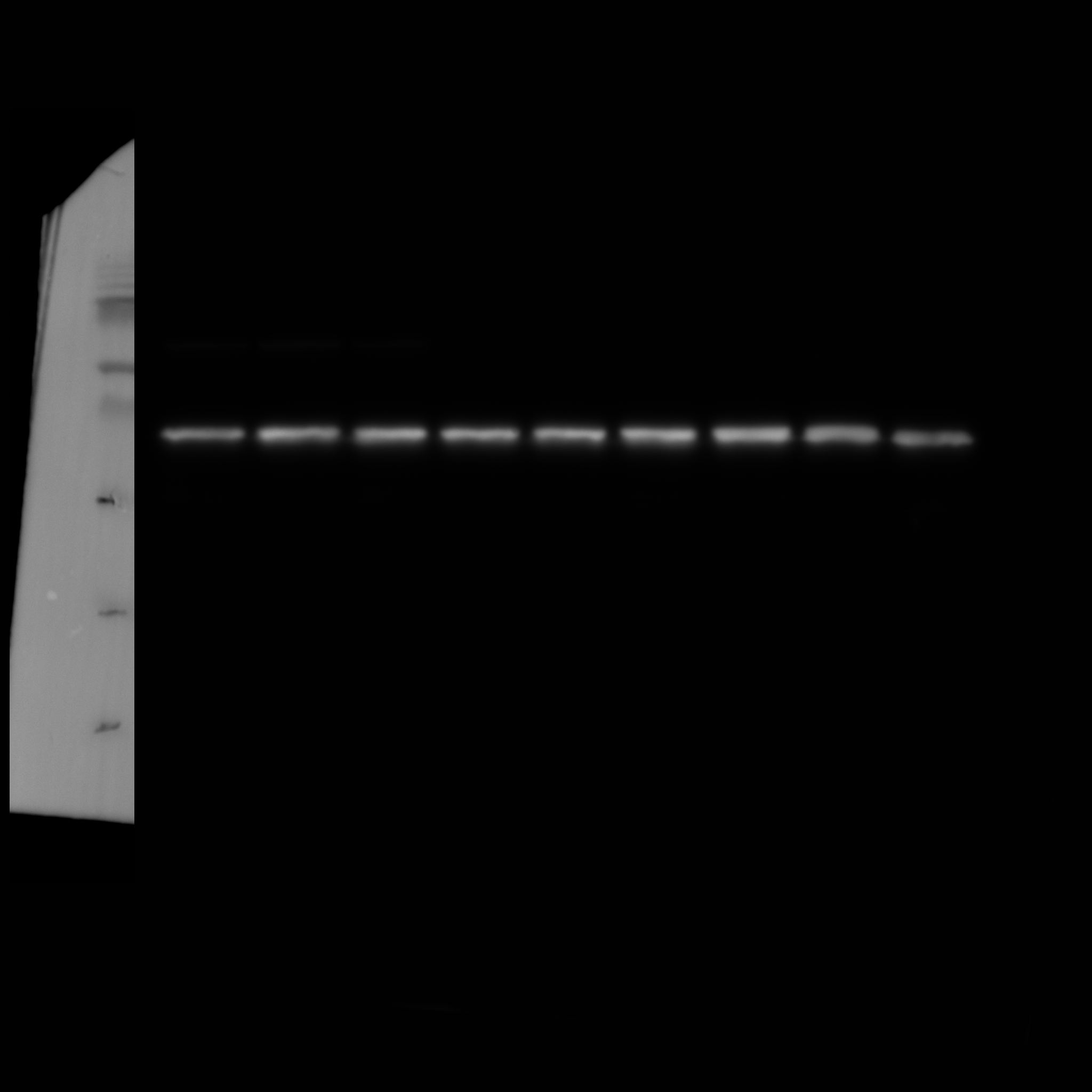

Supplement: Supplementary file 1 [file ijms-26-05476-s001.zip › Supplementary materials S1 Unprocessed immunoblots/Fig.3_AKT (Akti)/Figure 3C_241219_2_blot1_AK13-1_0%FCS_Akti_1uM_time_v.1.0_ VDAC1 (rb201).Tif]

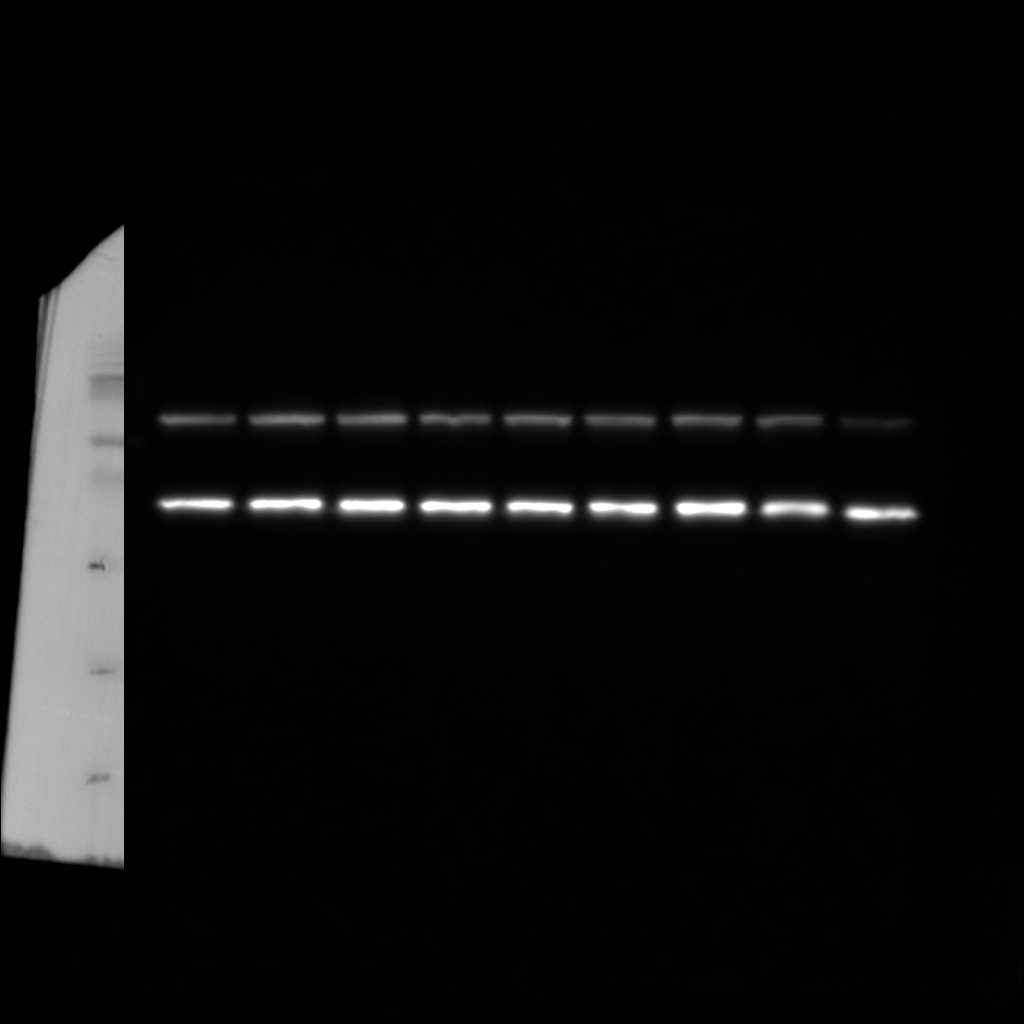

Supplement: Supplementary file 1 [file ijms-26-05476-s001.zip › Supplementary materials S1 Unprocessed immunoblots/Fig.3_AKT (Akti)/Figure 3C_241219_3_blot1_AK13-1_0%FCS_Akti_1uM_time_v.1.0_Akt (rb361).Tif]

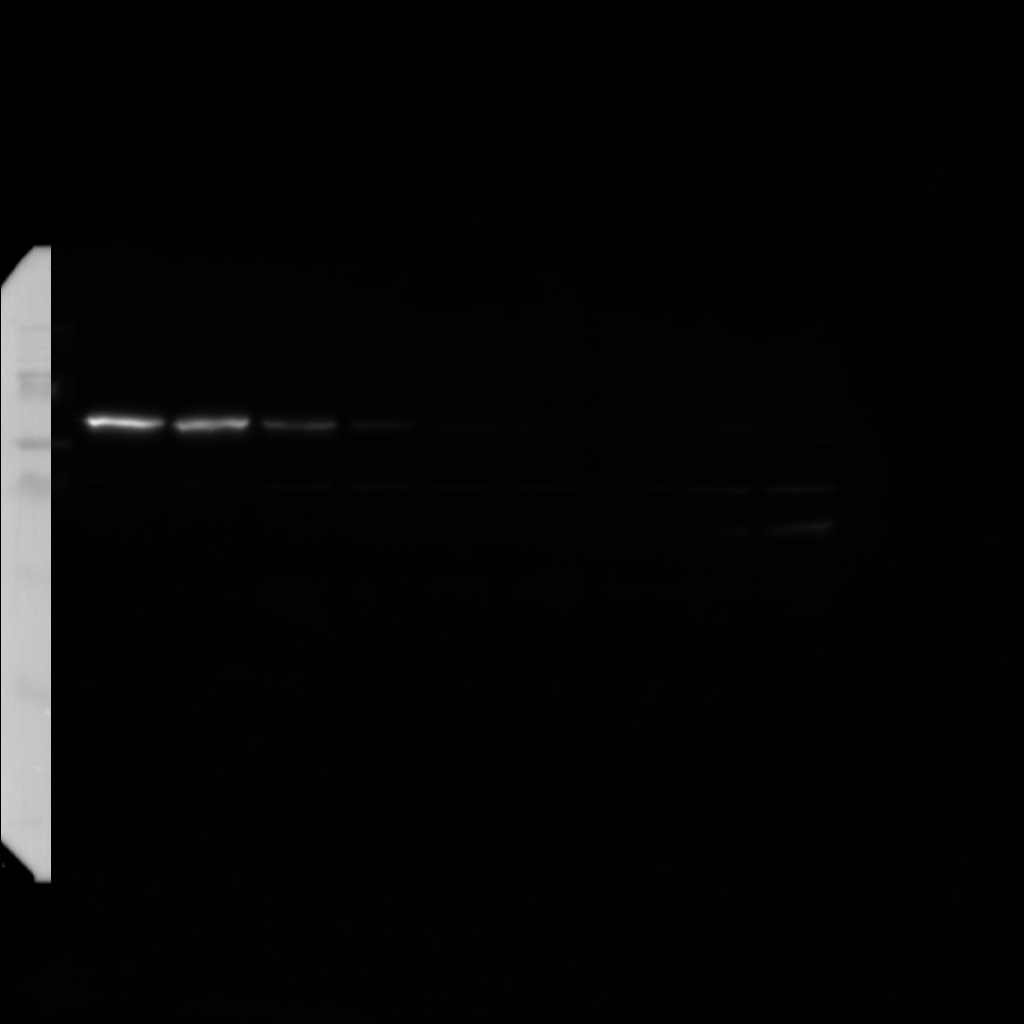

Supplement: Supplementary file 1 [file ijms-26-05476-s001.zip › Supplementary materials S1 Unprocessed immunoblots/Fig.3_AKT (Akti)/not shown_241219_1_blot2_AK13-1_0%FCS_Akti_1uM_time_v.1.1_pAkt (rb362).Tif]

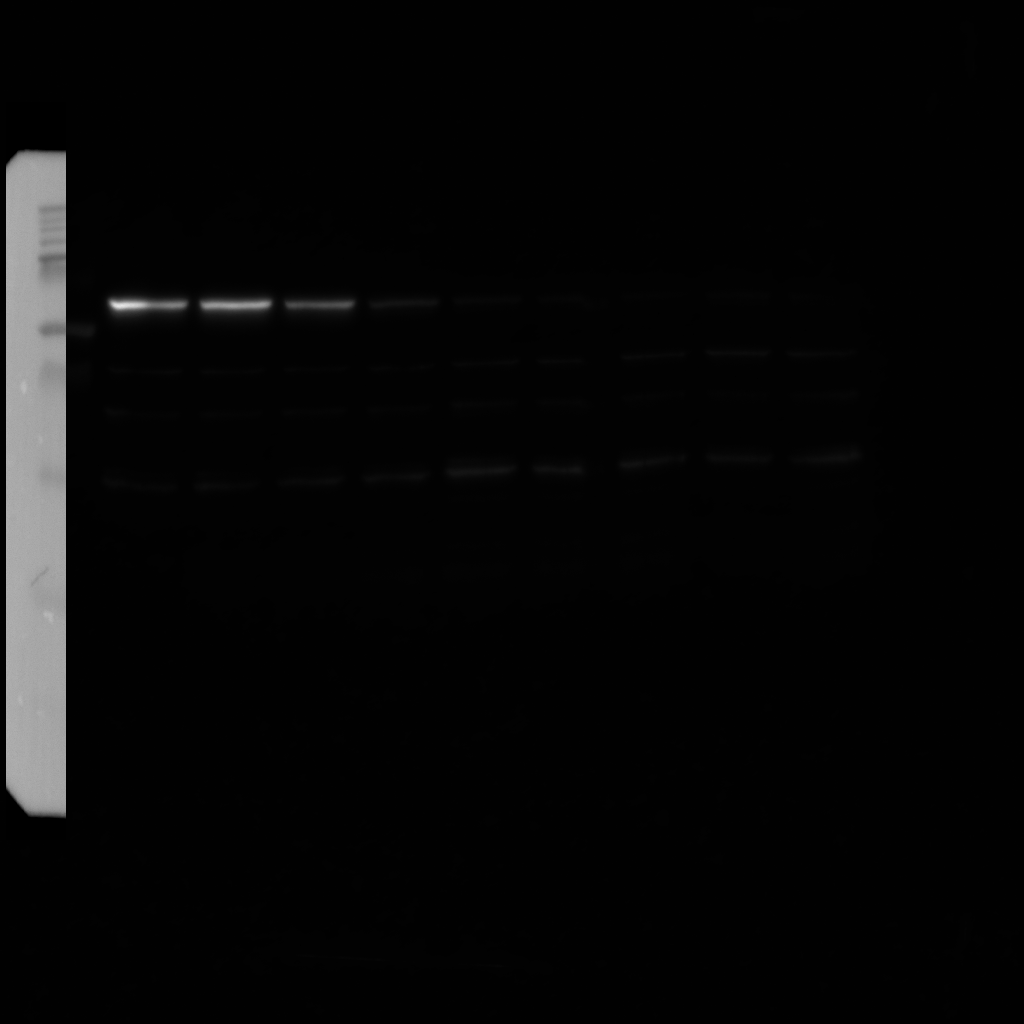

Supplement: Supplementary file 1 [file ijms-26-05476-s001.zip › Supplementary materials S1 Unprocessed immunoblots/Fig.3_AKT (Akti)/not shown_241219_1_blot3_AK13-1_0%FCS_Akti_1uM_time_v.1.2_pAkt (rb362).Tif]

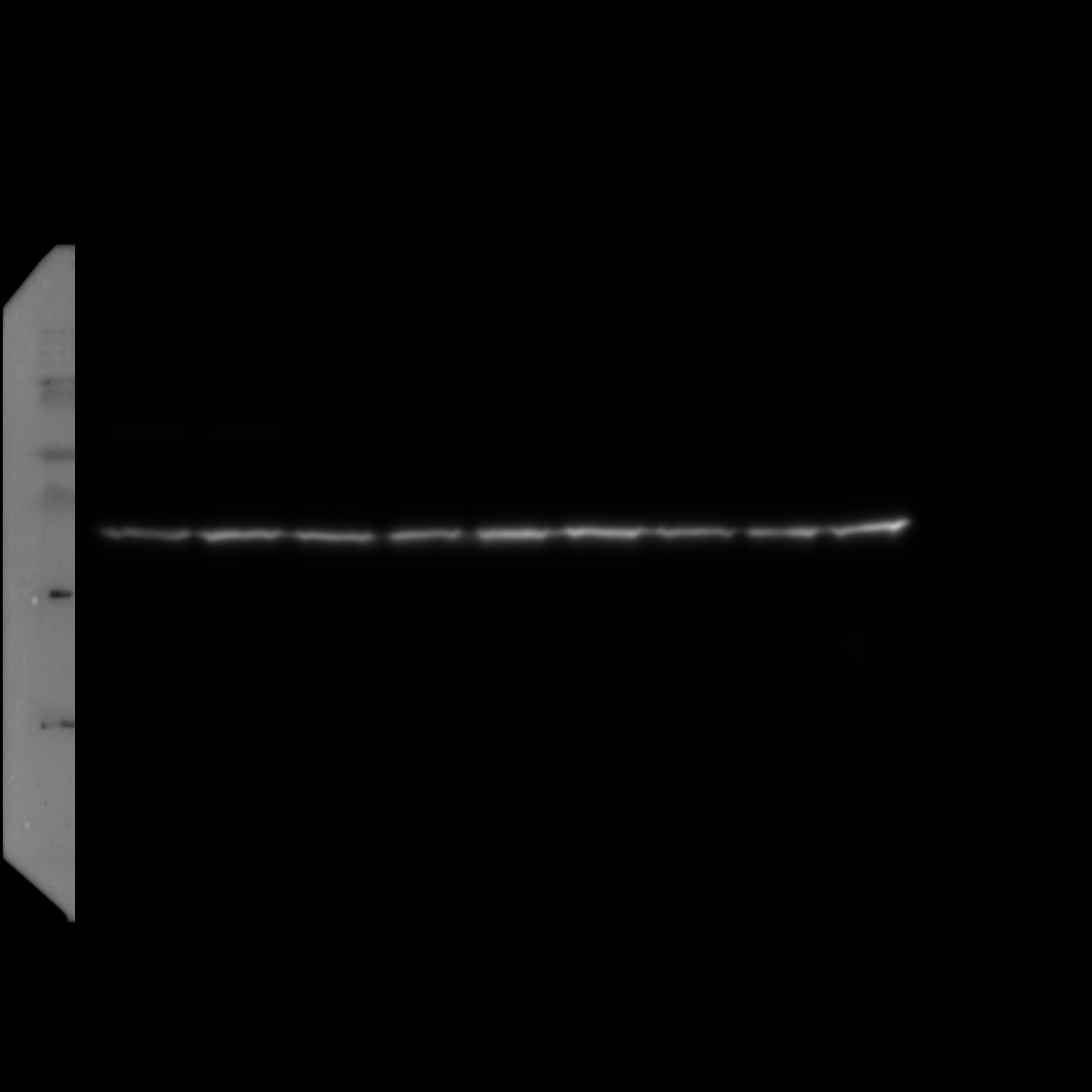

Supplement: Supplementary file 1 [file ijms-26-05476-s001.zip › Supplementary materials S1 Unprocessed immunoblots/Fig.3_AKT (Akti)/not shown_241219_2_blot2_AK13-1_0%FCS_Akti_1uM_time_v.1.1_VDAC1 (rb201).Tif]

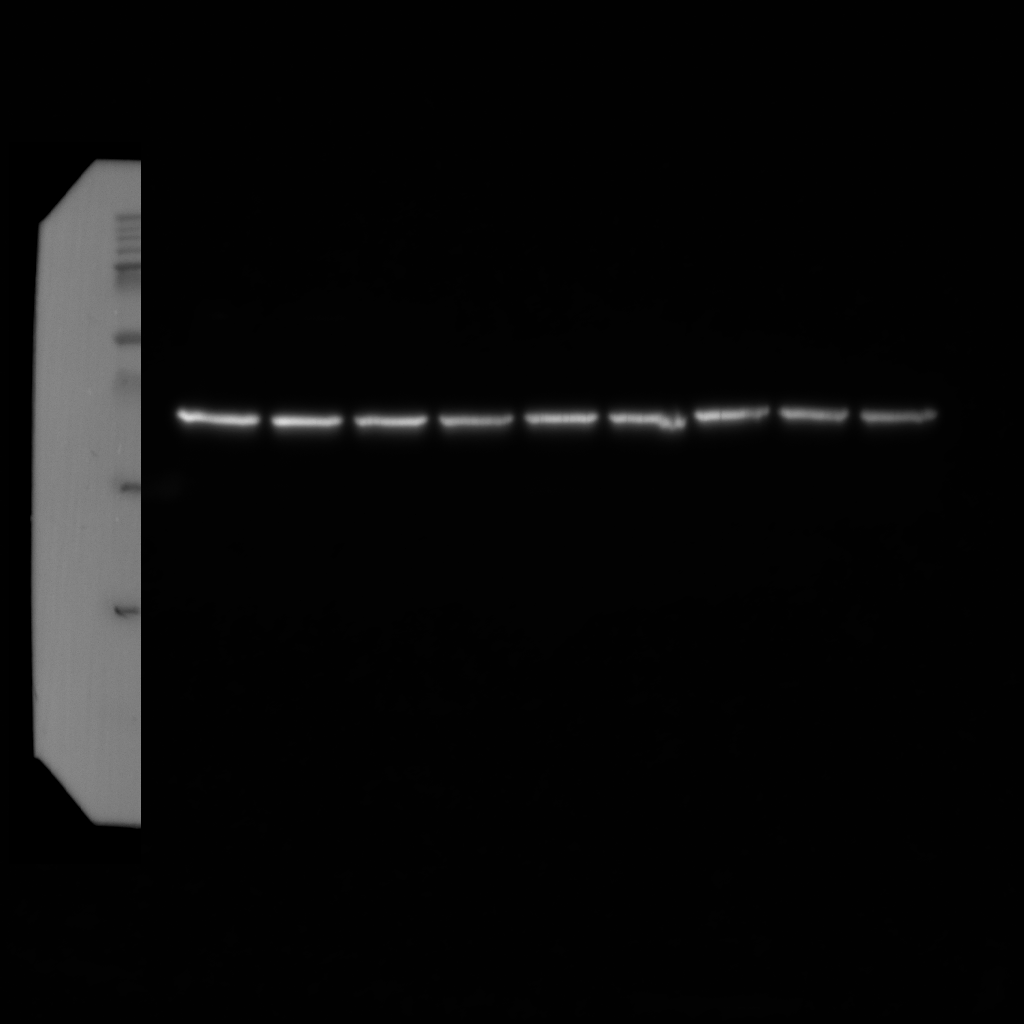

Supplement: Supplementary file 1 [file ijms-26-05476-s001.zip › Supplementary materials S1 Unprocessed immunoblots/Fig.3_AKT (Akti)/not shown_241219_2_blot3_AK13-1_0%FCS_Akti_1uM_time_v.1.2_VDAC1(rb201).Tif]

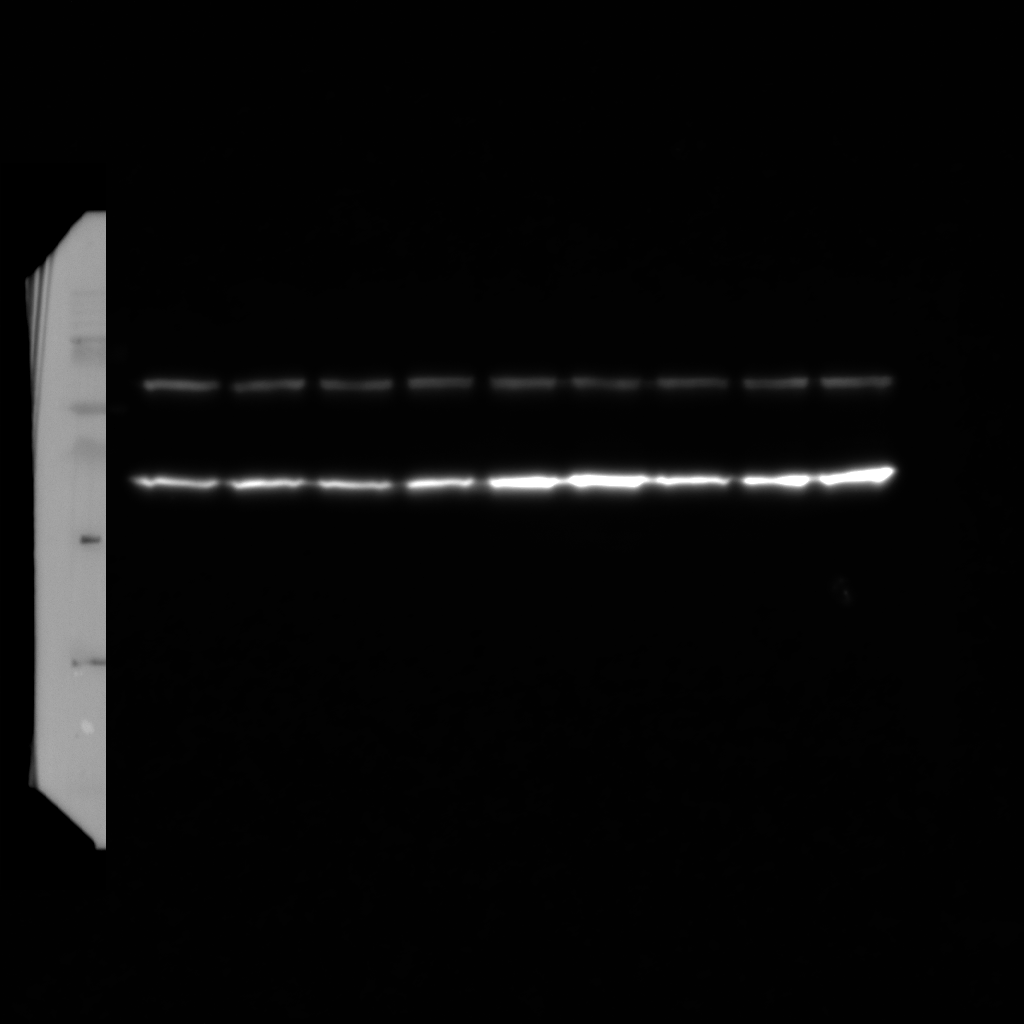

Supplement: Supplementary file 1 [file ijms-26-05476-s001.zip › Supplementary materials S1 Unprocessed immunoblots/Fig.3_AKT (Akti)/not shown_241219_3_blot2_AK13-1_0%FCS_Akti_1uM_time_v.1.1_Akt (rb361).Tif]

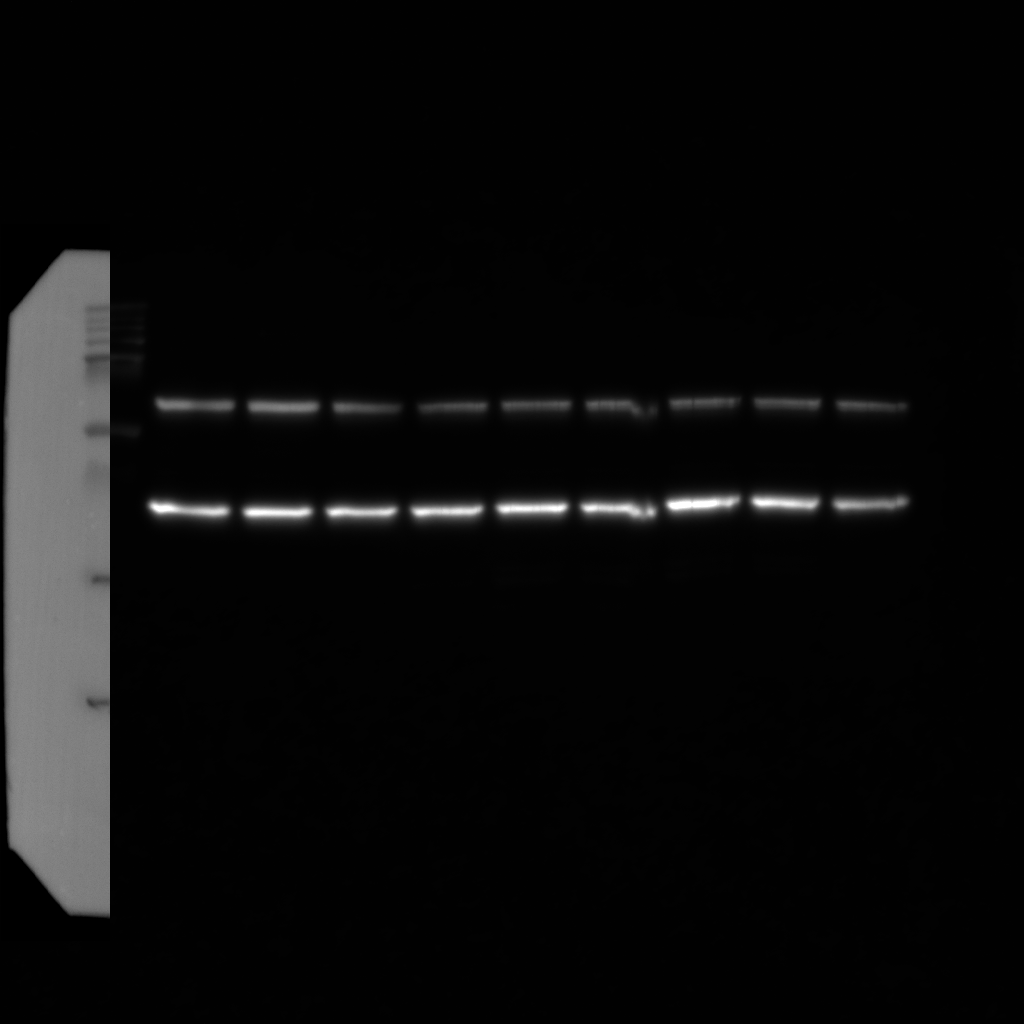

Supplement: Supplementary file 1 [file ijms-26-05476-s001.zip › Supplementary materials S1 Unprocessed immunoblots/Fig.3_AKT (Akti)/not shown_241219_3_blot3_AK13-1_0%FCS_Akti_1uM_time_v.1.2_Akt (rb361).Tif]

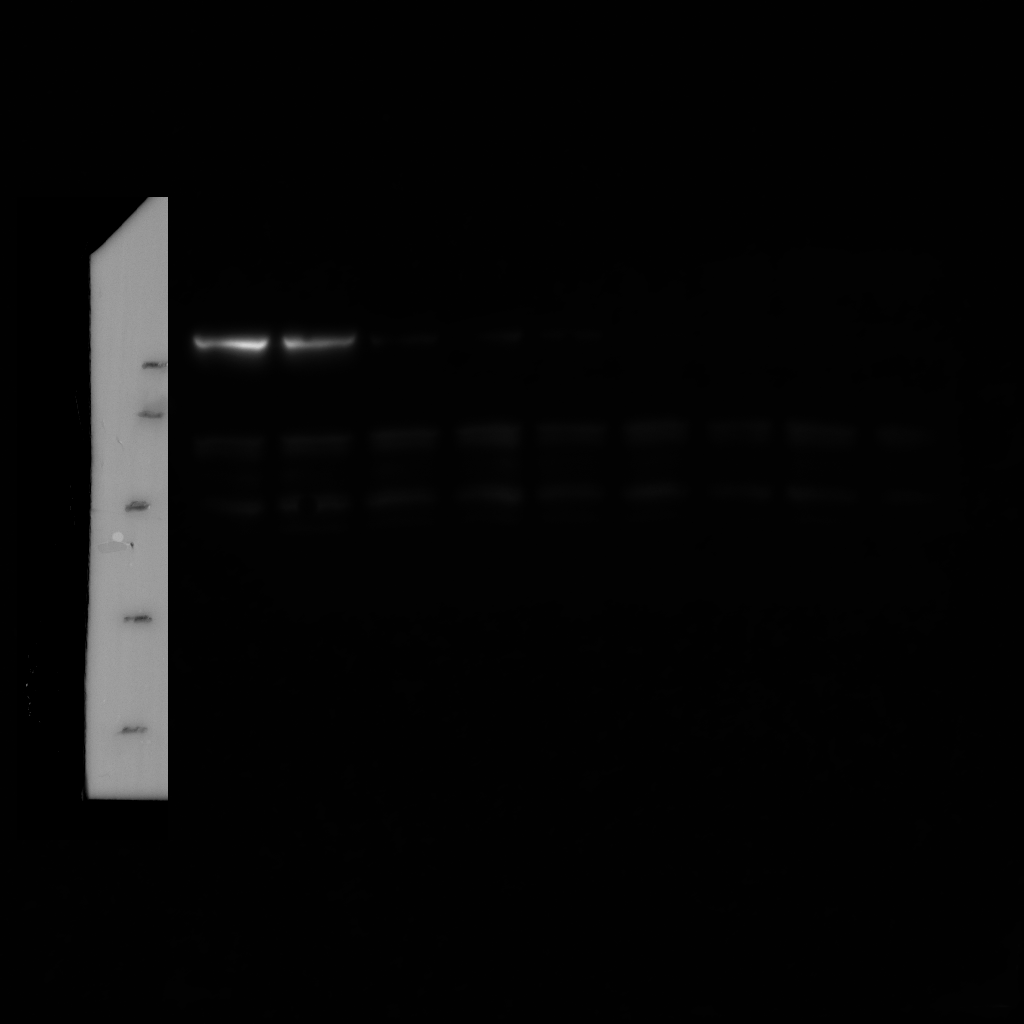

Supplement: Supplementary file 1 [file ijms-26-05476-s001.zip › Supplementary materials S1 Unprocessed immunoblots/Fig.3_AKT (PI828)/Figure 3A_241216_1_blot1_AK13-1_0%FCS_PI828_0-30min_v.1.0_pAkt (rb362).Tif]

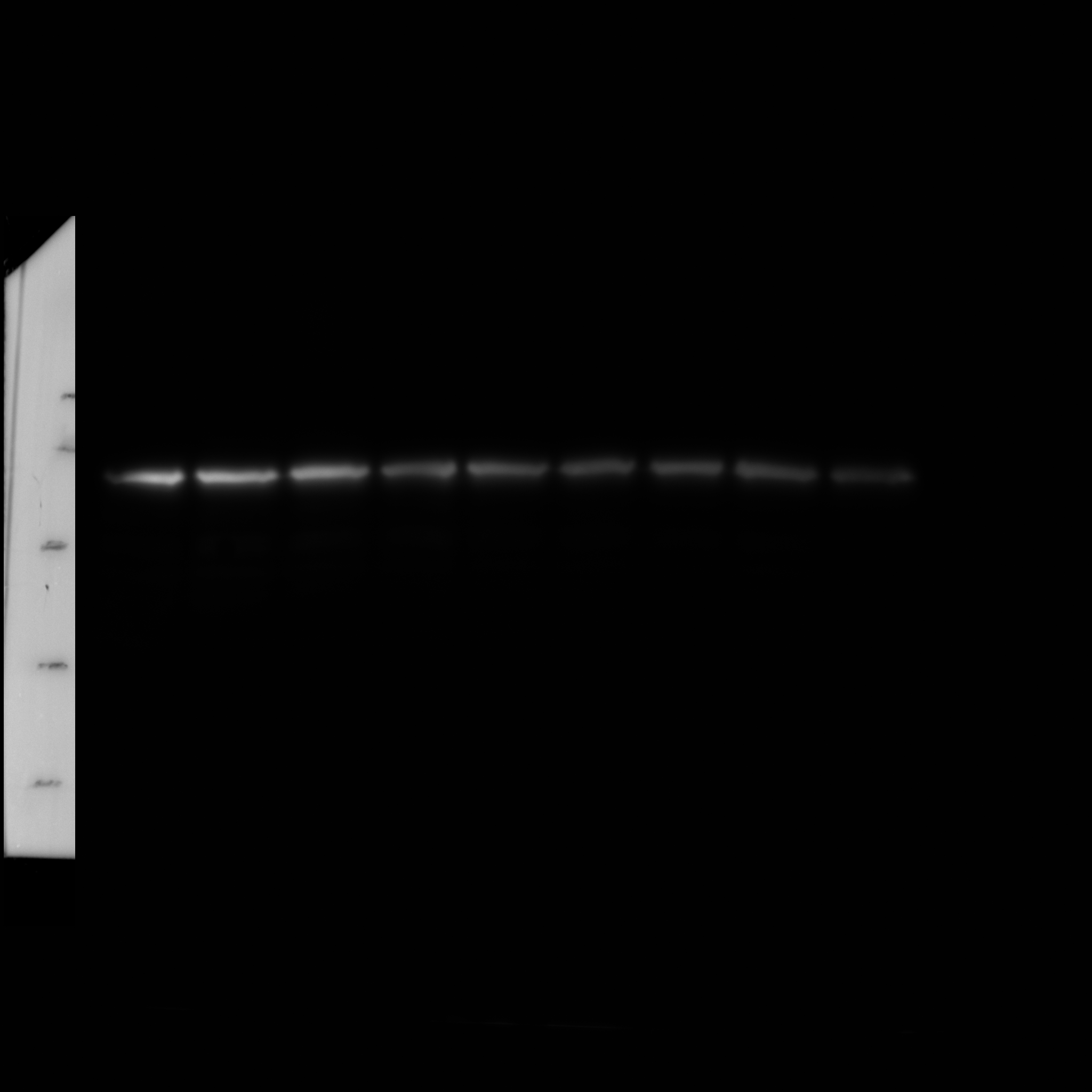

Supplement: Supplementary file 1 [file ijms-26-05476-s001.zip › Supplementary materials S1 Unprocessed immunoblots/Fig.3_AKT (PI828)/Figure 3A_241216_2_blot1_AK13-1_0%FCS_PI828_0-30min_v.1.0_VDAC1 (rb201).Tif]

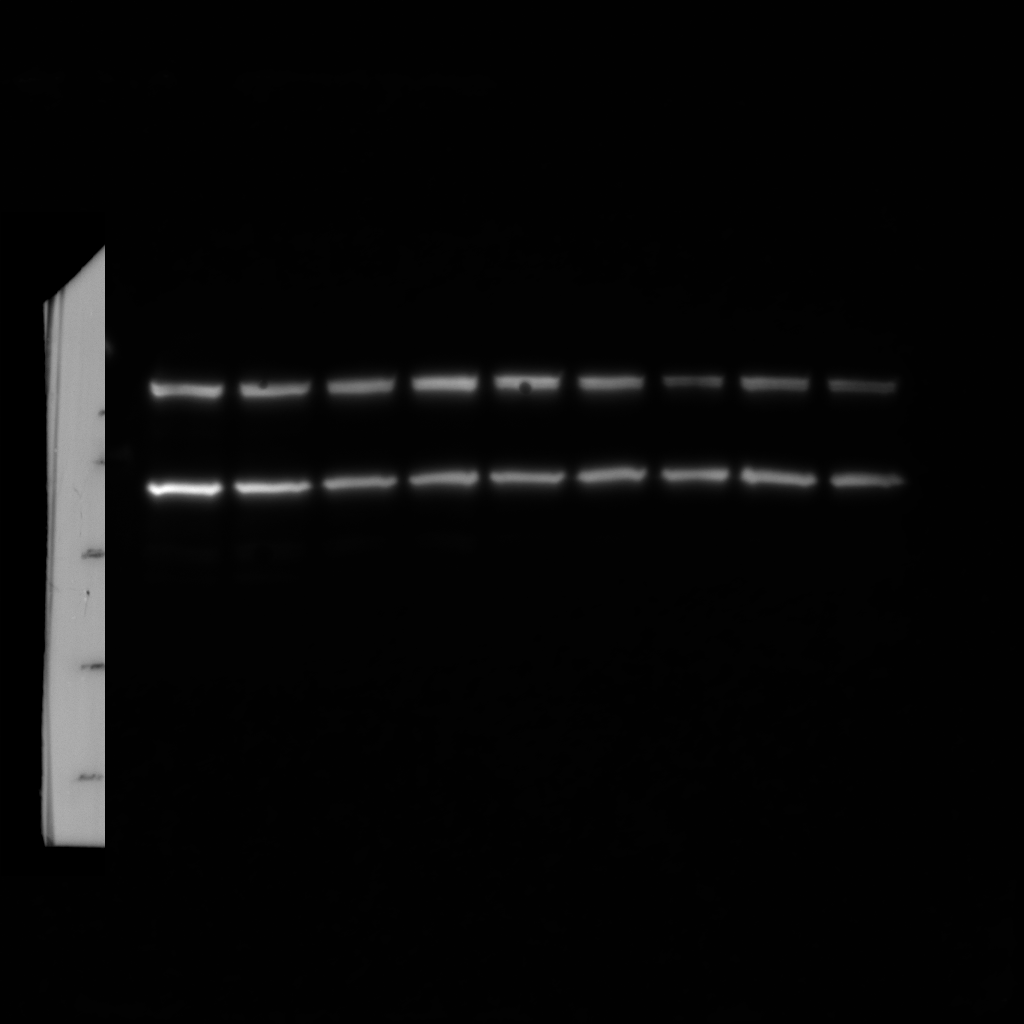

Supplement: Supplementary file 1 [file ijms-26-05476-s001.zip › Supplementary materials S1 Unprocessed immunoblots/Fig.3_AKT (PI828)/Figure 3A_241216_3_blot1_AK13-1_0%FCS_PI828_0-30min_v.1.0_Akt (rb361).Tif]

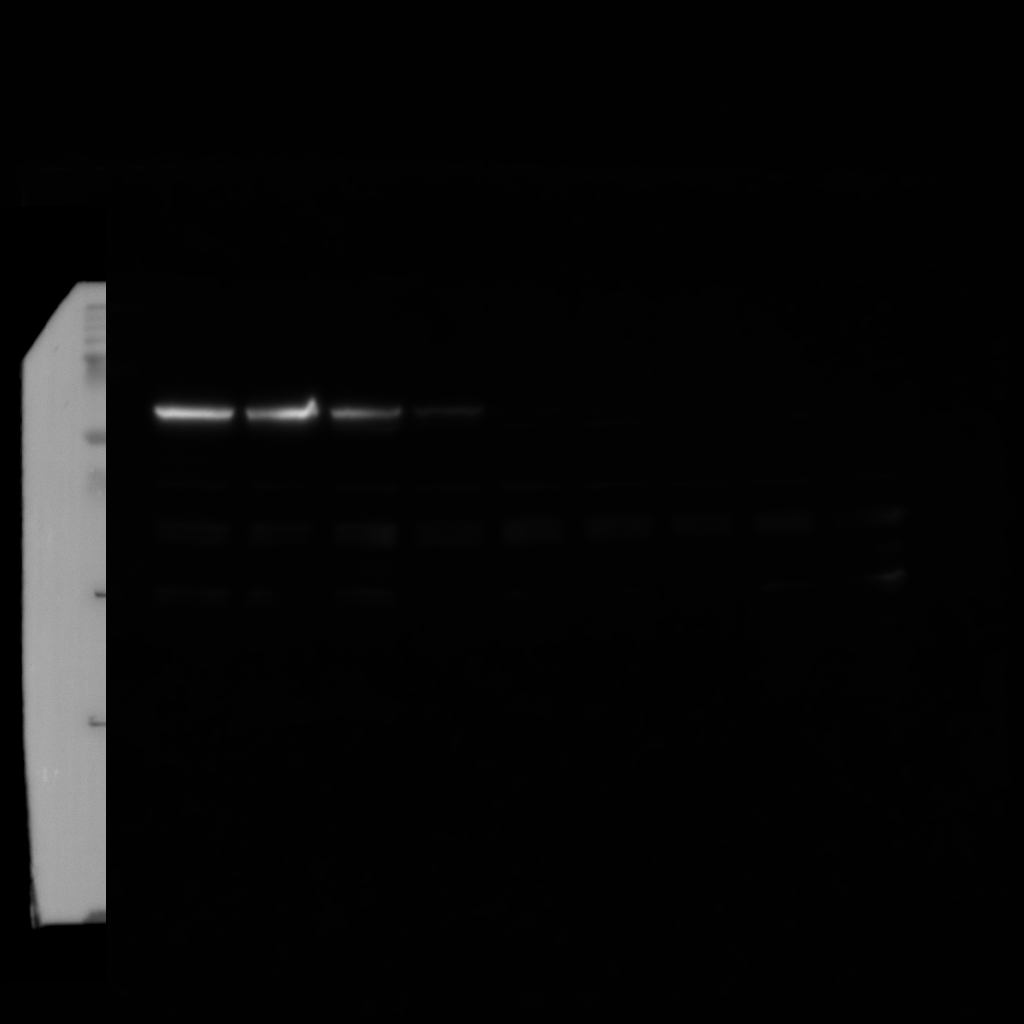

Supplement: Supplementary file 1 [file ijms-26-05476-s001.zip › Supplementary materials S1 Unprocessed immunoblots/Fig.3_AKT (PI828)/not shown_241216_redone_1_blot4_AK13-1_0%FCS_PI828_0-30min_v.1.1_pAkt (rb362).Tif]

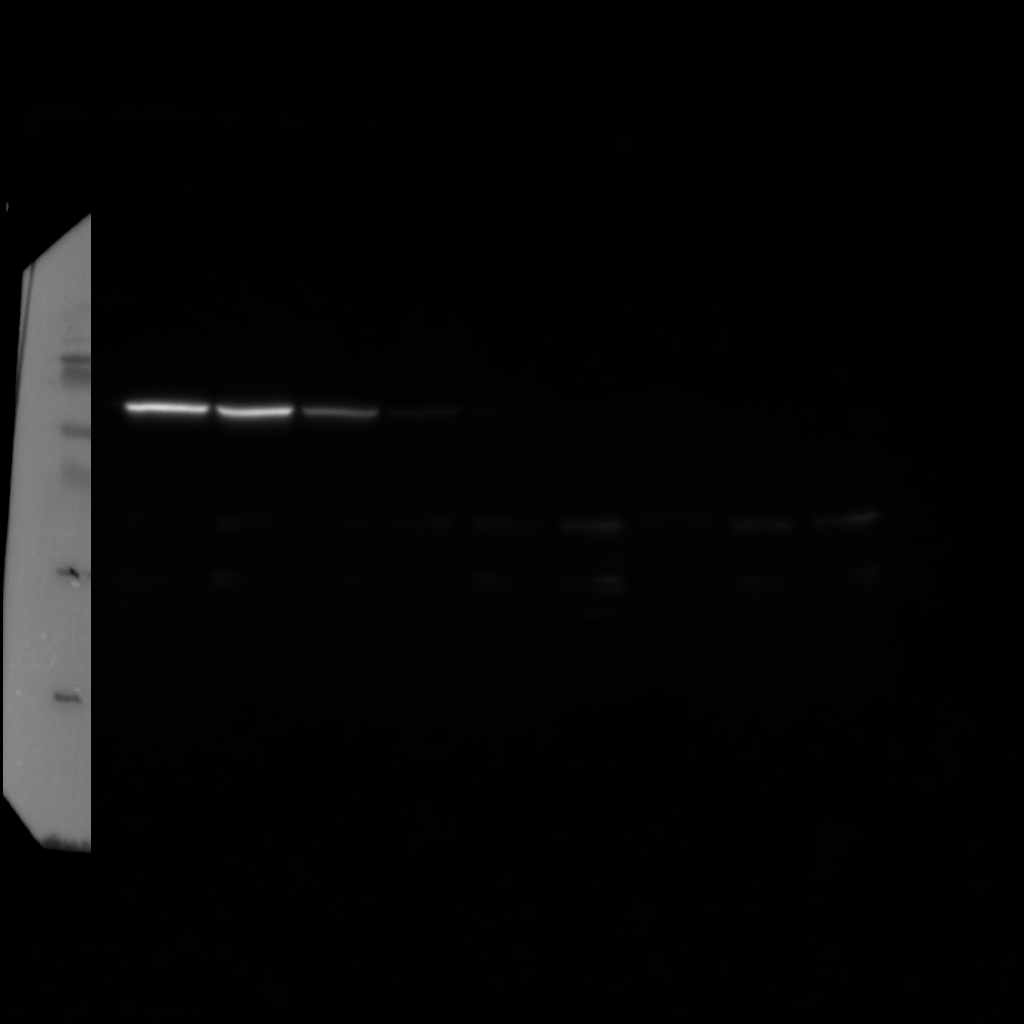

Supplement: Supplementary file 1 [file ijms-26-05476-s001.zip › Supplementary materials S1 Unprocessed immunoblots/Fig.3_AKT (PI828)/not shown_241216_redone_1_blot5_AK13-1_0%FCS_PI828_0-30min_v.1.2_pAkt (rb362).Tif]

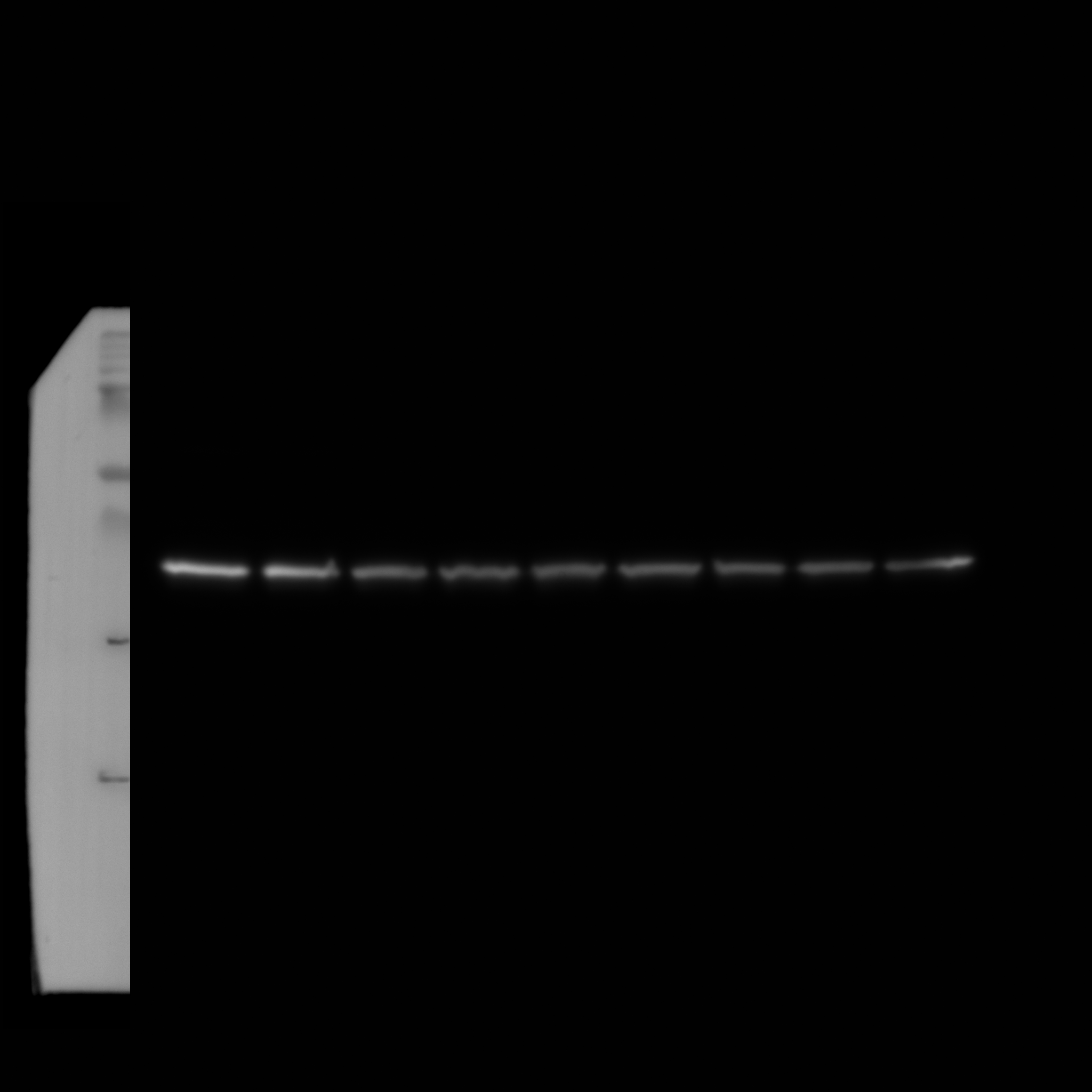

Supplement: Supplementary file 1 [file ijms-26-05476-s001.zip › Supplementary materials S1 Unprocessed immunoblots/Fig.3_AKT (PI828)/not shown_241216_redone_2_blot4_AK13-1_0%FCS_PI828_0-30min_v.1.1_VDAC1 (rb201).Tif]

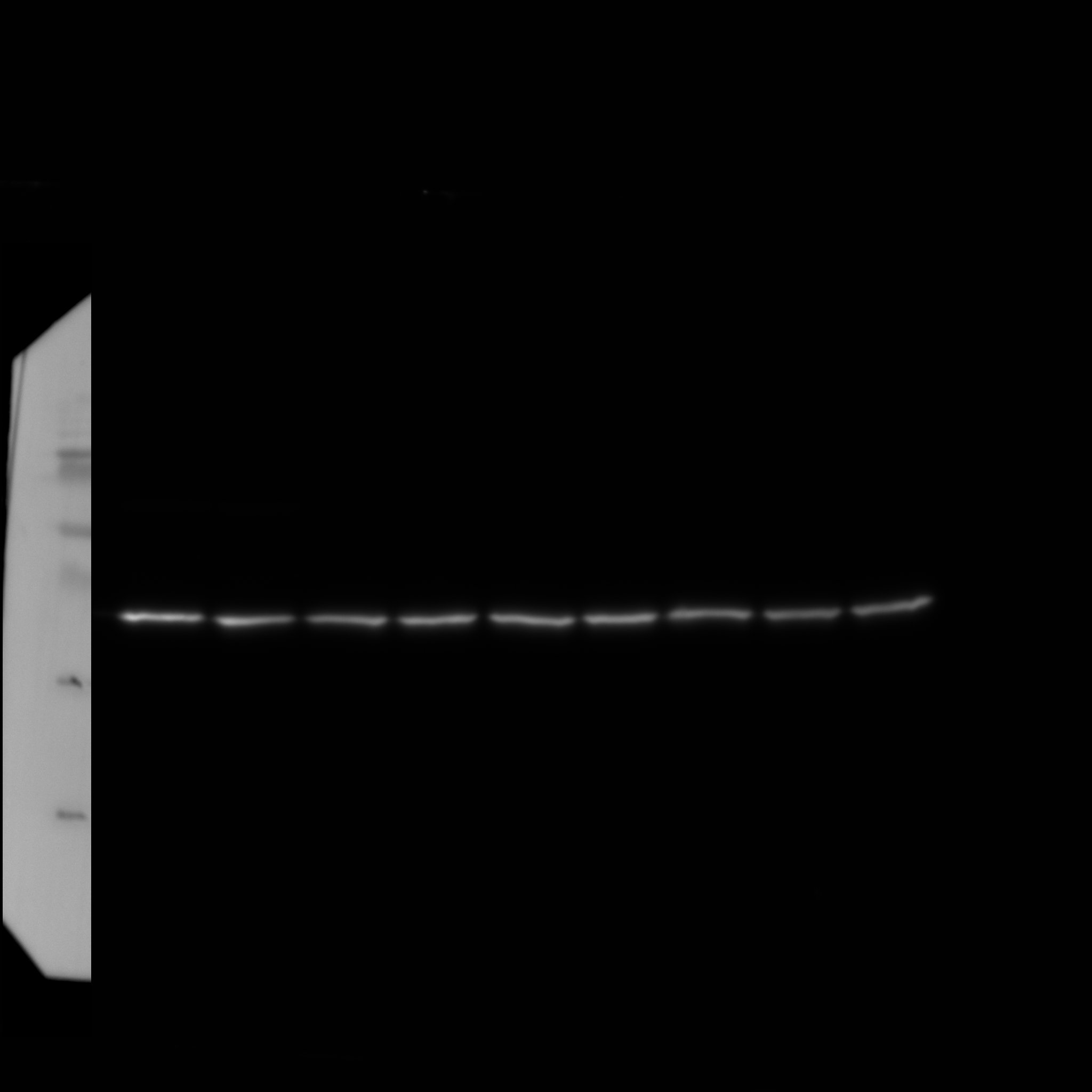

Supplement: Supplementary file 1 [file ijms-26-05476-s001.zip › Supplementary materials S1 Unprocessed immunoblots/Fig.3_AKT (PI828)/not shown_241216_redone_2_blot5_AK13-1_0%FCS_PI828_0-30min_v.1.2_VDAC1 (rb201).Tif]

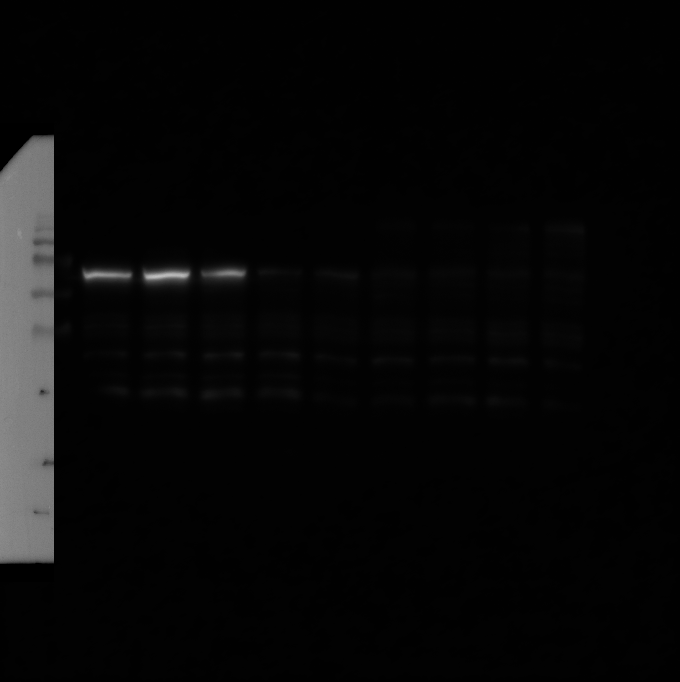

Supplement: Supplementary file 1 [file ijms-26-05476-s001.zip › Supplementary materials S1 Unprocessed immunoblots/Fig.3_AKT (Wortmannin)/Figure 3A_241215_1_blot1_AK13-1_5uM_Wort_0-30min_v.1.1_pAkt (rb362).Tif]

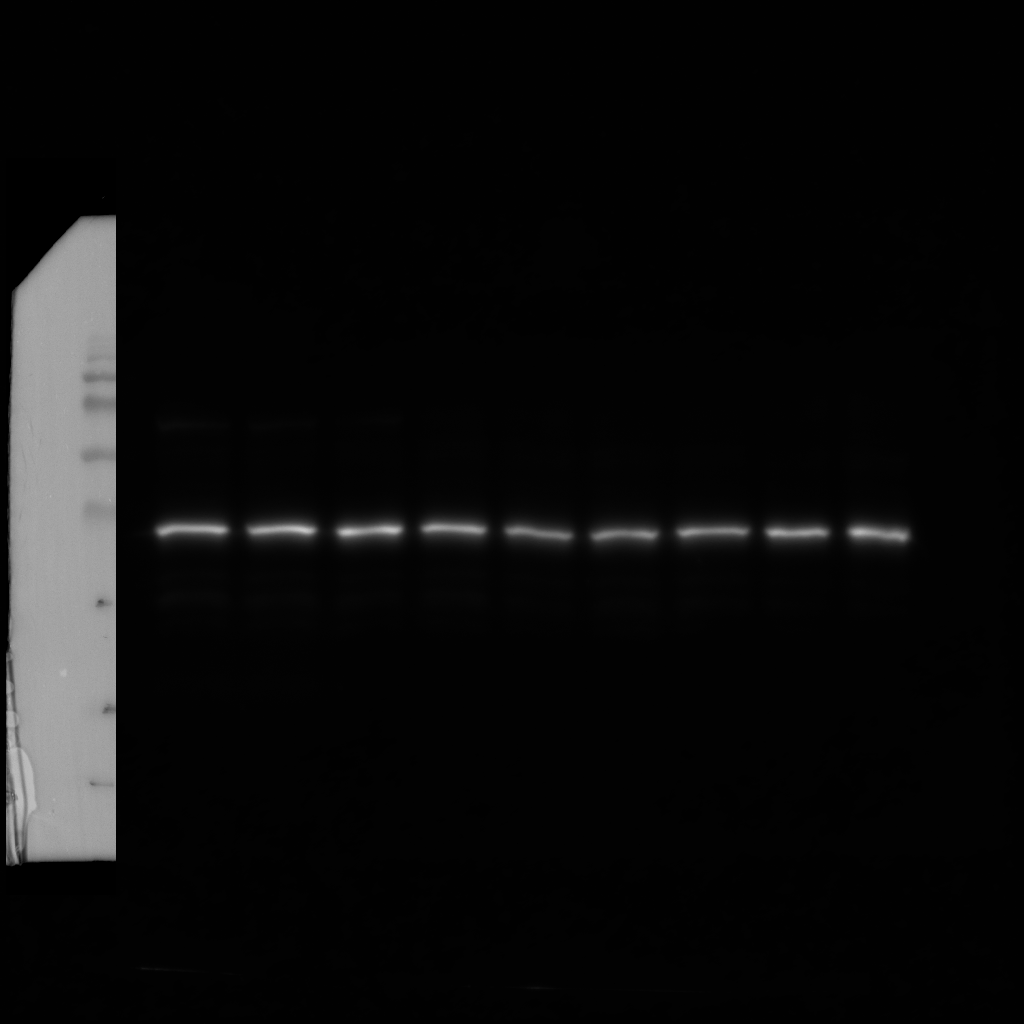

Supplement: Supplementary file 1 [file ijms-26-05476-s001.zip › Supplementary materials S1 Unprocessed immunoblots/Fig.3_AKT (Wortmannin)/Figure 3A_241215_3_blot1_AK13-1_5uM_Wort_0-30min_v.1.1_VDAC1 (rb201).Tif]

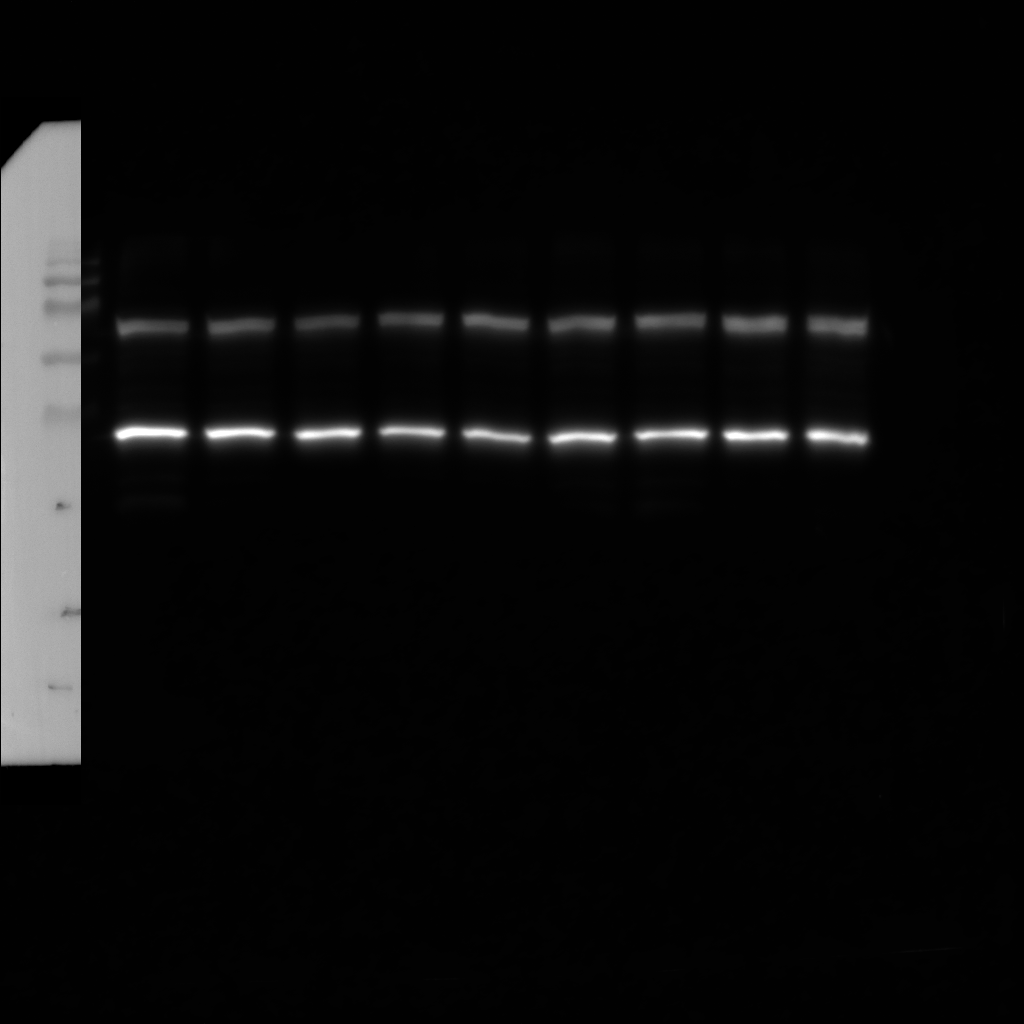

Supplement: Supplementary file 1 [file ijms-26-05476-s001.zip › Supplementary materials S1 Unprocessed immunoblots/Fig.3_AKT (Wortmannin)/Figure 3A_241215_4_blot1_AK13-1_5uM_Wort_0-30min_v.1.1_Akt (rb361).Tif]

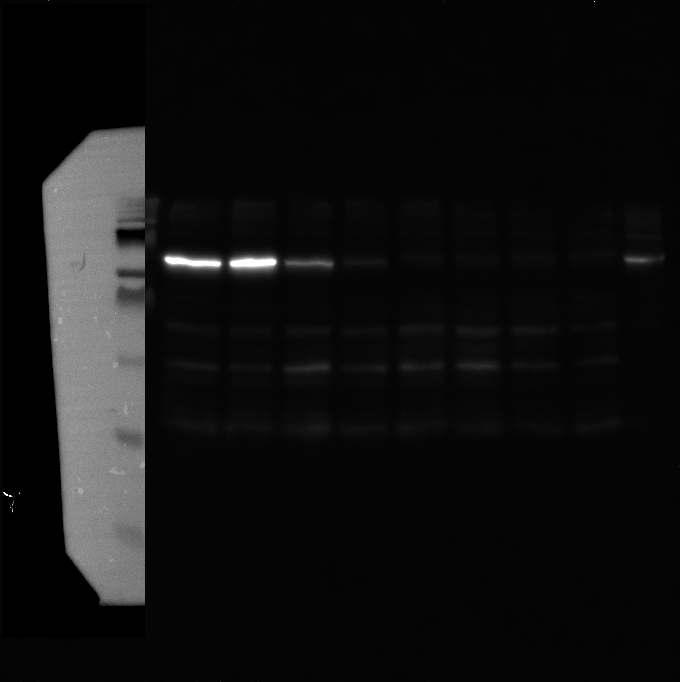

Supplement: Supplementary file 1 [file ijms-26-05476-s001.zip › Supplementary materials S1 Unprocessed immunoblots/Fig.3_AKT (Wortmannin)/not shown_240430_blot2_1_AK13-1_5uM_Wort_0-30min_pAkt (rb362).Tif]

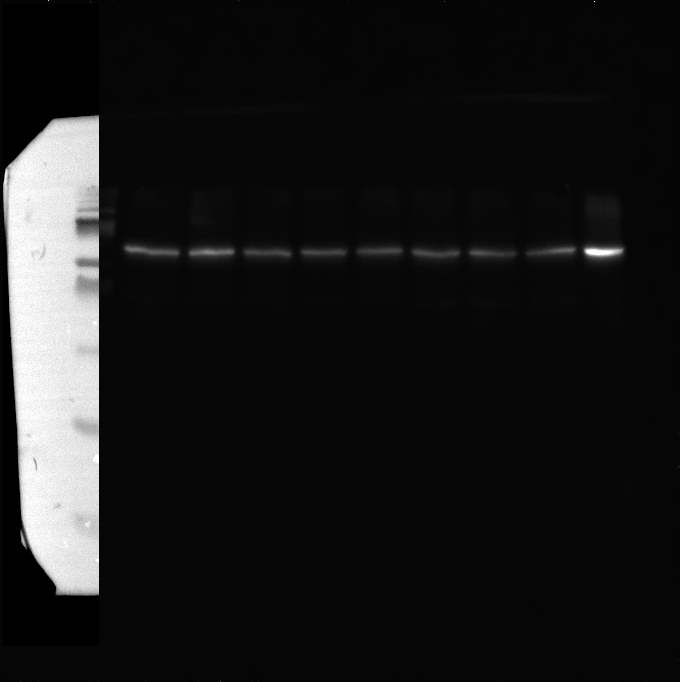

Supplement: Supplementary file 1 [file ijms-26-05476-s001.zip › Supplementary materials S1 Unprocessed immunoblots/Fig.3_AKT (Wortmannin)/not shown_240430_blot2_2_AK13-1_5uM_Wort_0-30min_Akt (rb361).Tif]

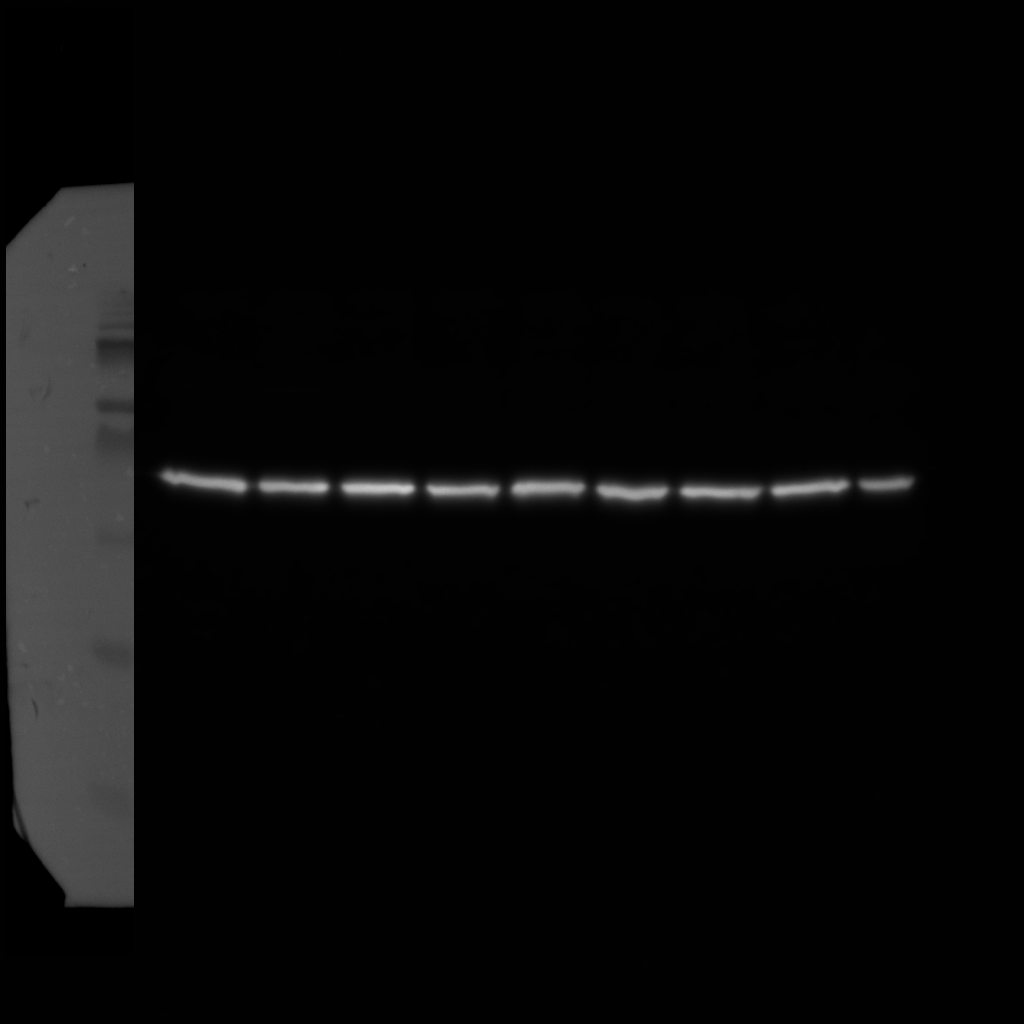

Supplement: Supplementary file 1 [file ijms-26-05476-s001.zip › Supplementary materials S1 Unprocessed immunoblots/Fig.3_AKT (Wortmannin)/not shown_240430_blot2_3_AK13-1_5uM_Wort_0-30min_VDAC1 (rb201).Tif]

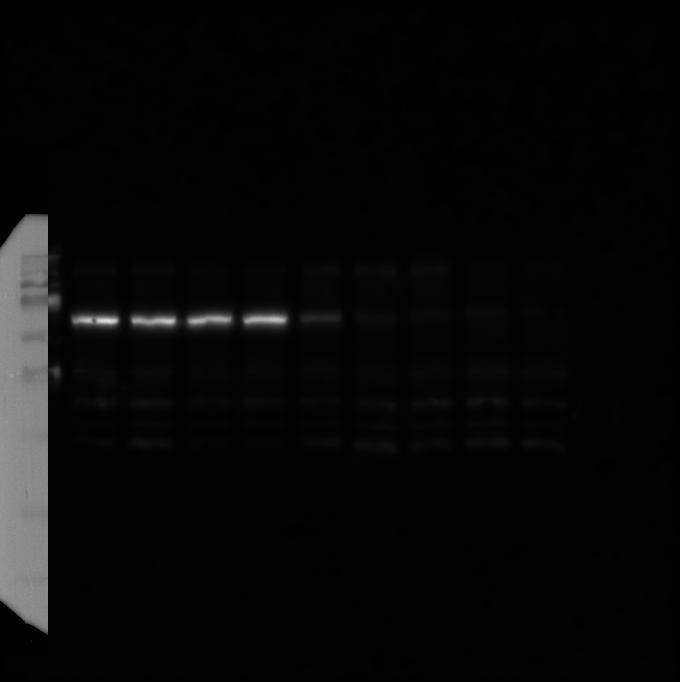

Supplement: Supplementary file 1 [file ijms-26-05476-s001.zip › Supplementary materials S1 Unprocessed immunoblots/Fig.3_AKT (Wortmannin)/not shown_241215_1_blot2_AK13-1_5uM_Wort_0-30min_v.1.2_pAkt (rb362).Tif]

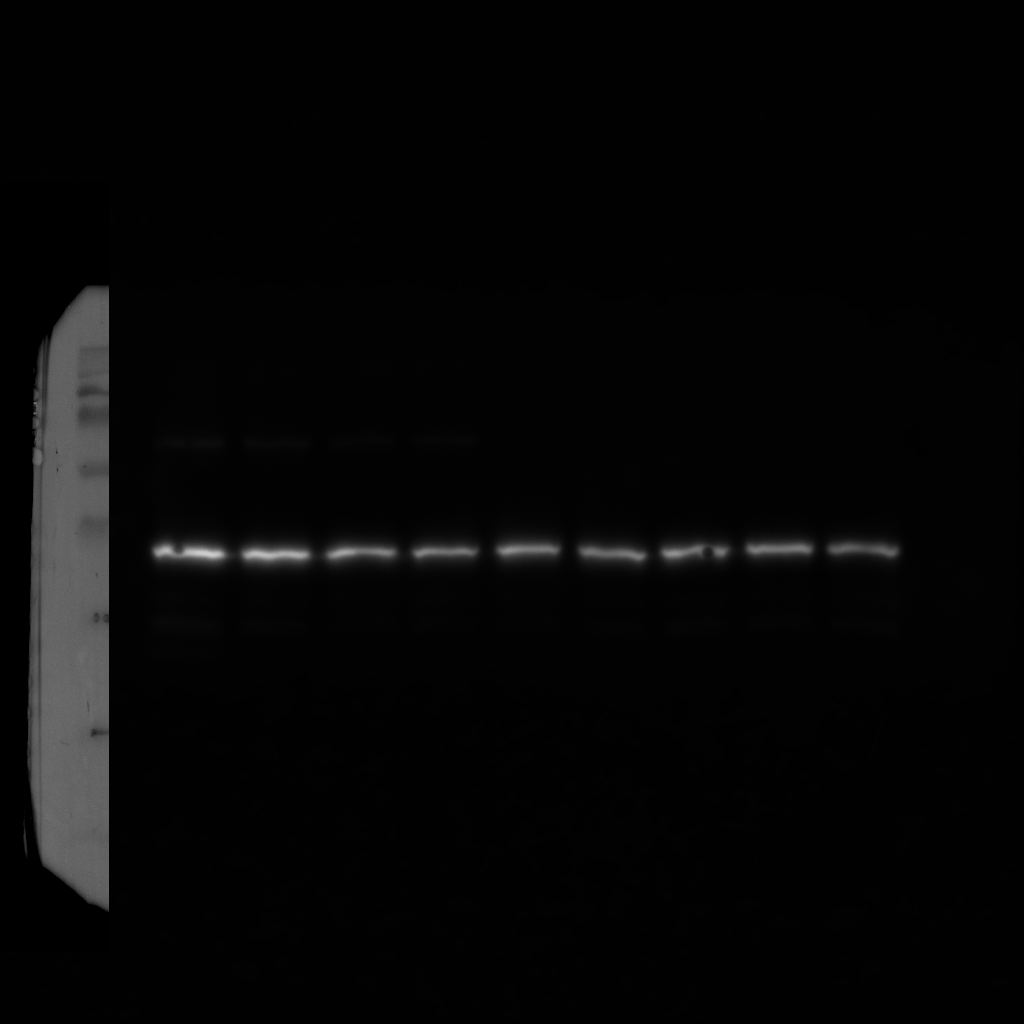

Supplement: Supplementary file 1 [file ijms-26-05476-s001.zip › Supplementary materials S1 Unprocessed immunoblots/Fig.3_AKT (Wortmannin)/not shown_241215_3_blot2_AK13-1_5uM_Wort_0-30min_v.1.2_VDAC1 (rb201).Tif]

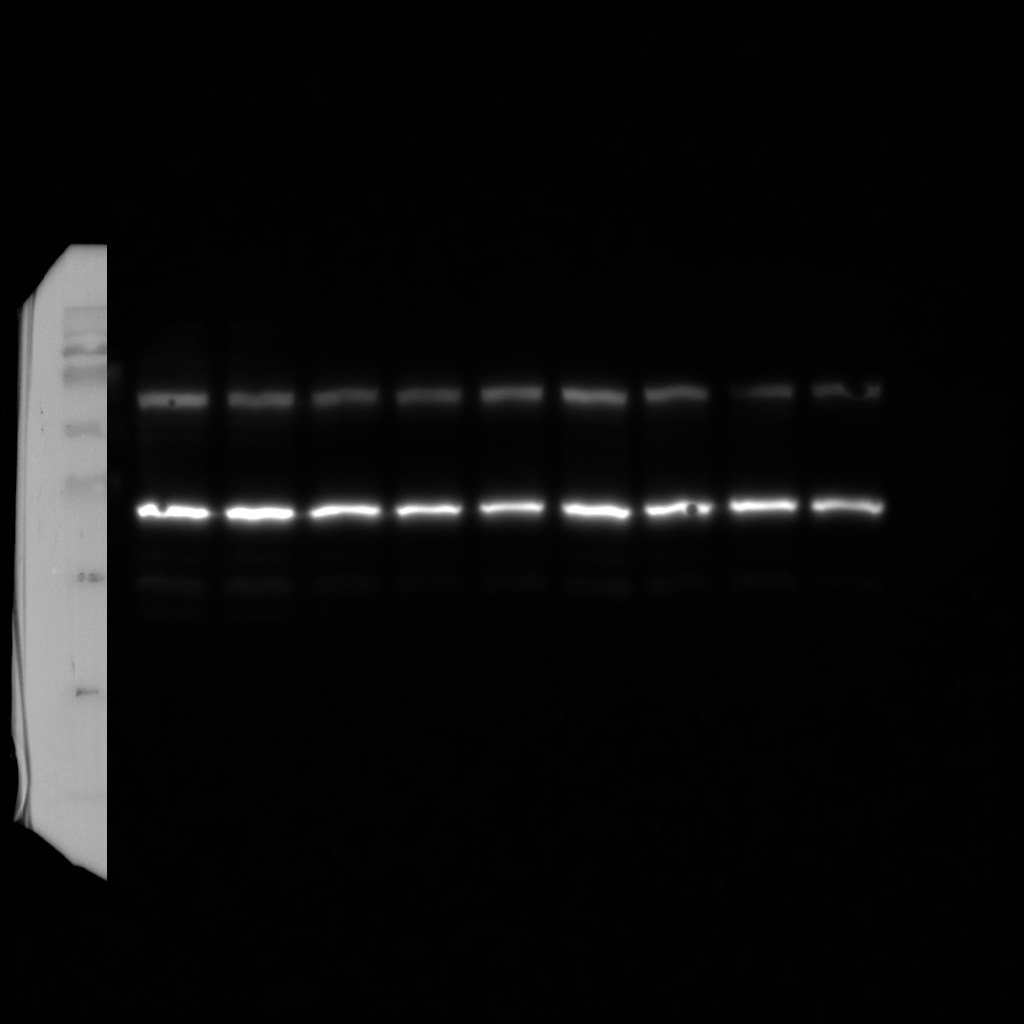

Supplement: Supplementary file 1 [file ijms-26-05476-s001.zip › Supplementary materials S1 Unprocessed immunoblots/Fig.3_AKT (Wortmannin)/not shown_241215_4_blot2_AK13-1_5uM_Wort_0-30min_v.1.2_Akt (rb361).Tif]

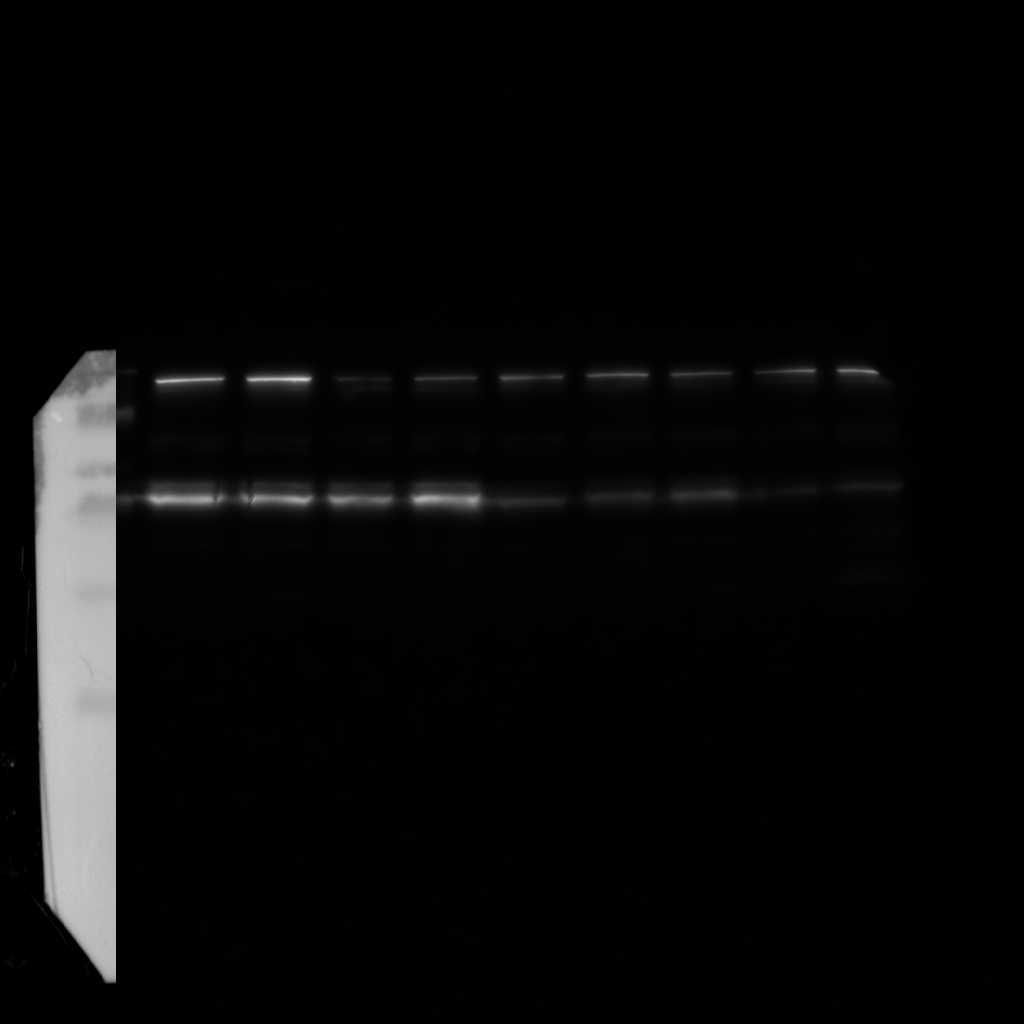

Supplement: Supplementary file 1 [file ijms-26-05476-s001.zip › Supplementary materials S1 Unprocessed immunoblots/Fig.4_ERK (FR180204)/Figure 4A_241221_1_blot1_AK13-1_0%FCS_FR-204_time_v.1.0_pERK (rb85).Tif]

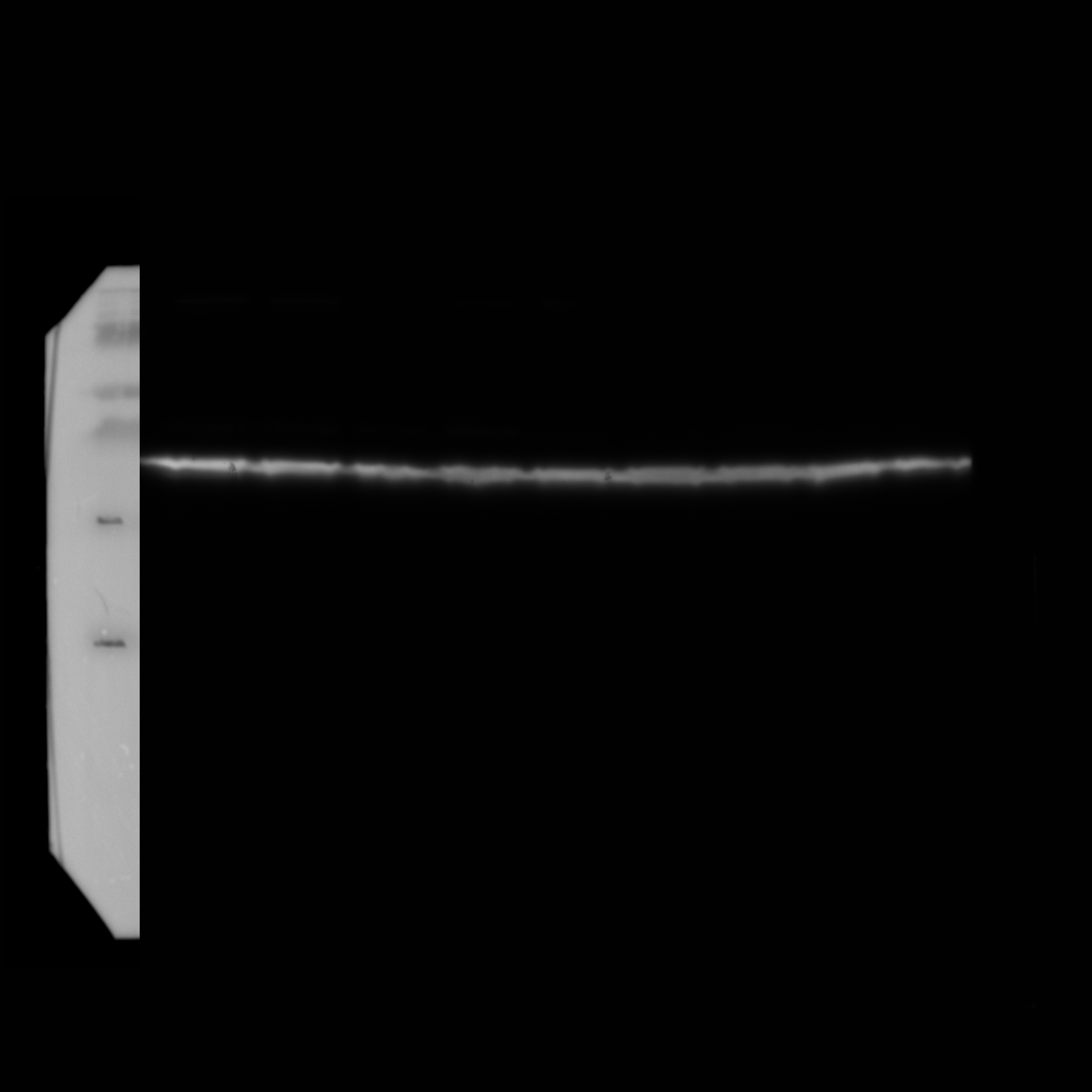

Supplement: Supplementary file 1 [file ijms-26-05476-s001.zip › Supplementary materials S1 Unprocessed immunoblots/Fig.4_ERK (FR180204)/Figure 4A_241221_2_blot1_AK13-1_FR-204_time_1.0_VDAC1 (rb201).Tif]

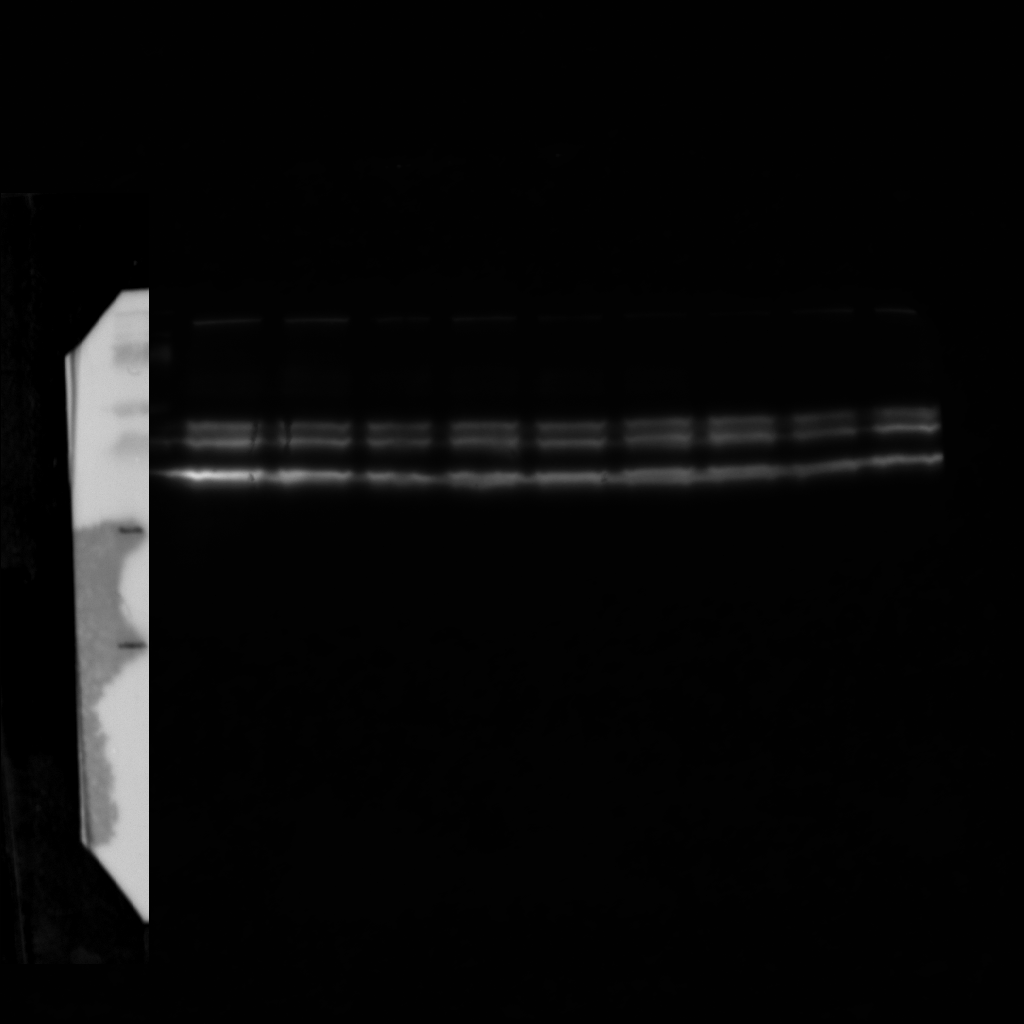

Supplement: Supplementary file 1 [file ijms-26-05476-s001.zip › Supplementary materials S1 Unprocessed immunoblots/Fig.4_ERK (FR180204)/Figure 4A_241221_3_blot1_AK13-1_FR-204_time_1.0_ERK (rb81).Tif]

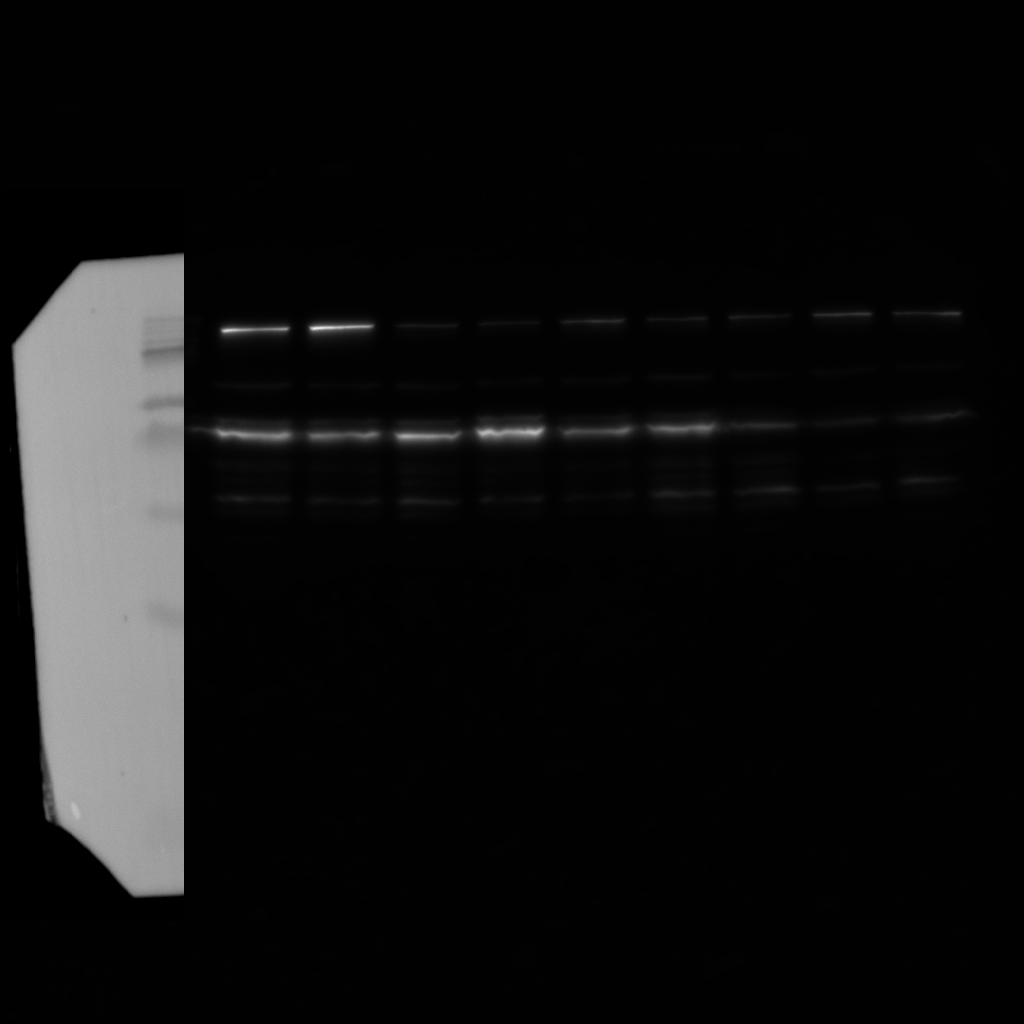

Supplement: Supplementary file 1 [file ijms-26-05476-s001.zip › Supplementary materials S1 Unprocessed immunoblots/Fig.4_ERK (FR180204)/not shown_241221_1_blot2_AK13-1_0%FCS_FR-204_time_v.1.1_pERK (rb85).Tif]

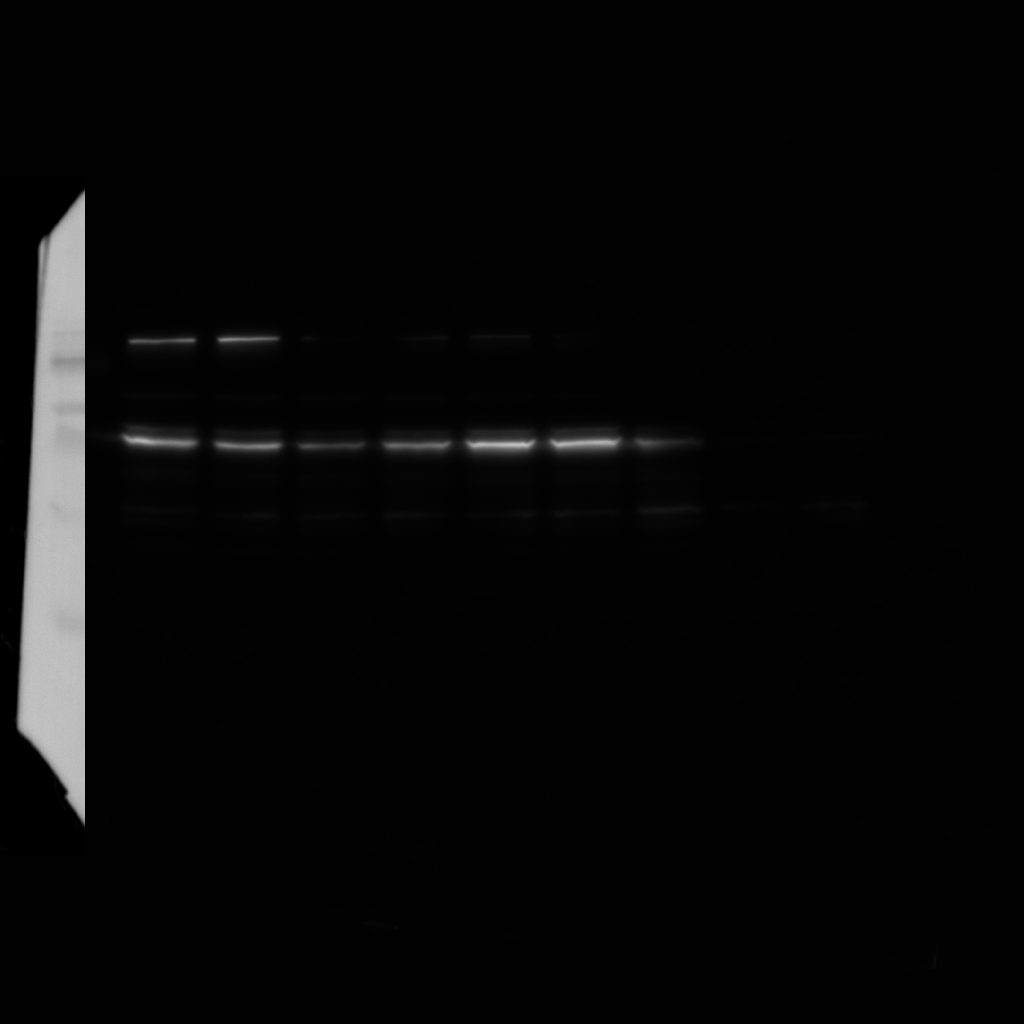

Supplement: Supplementary file 1 [file ijms-26-05476-s001.zip › Supplementary materials S1 Unprocessed immunoblots/Fig.4_ERK (FR180204)/not shown_241221_1_blot3_AK13-1_0%FCS_FR-204_time_v.1.2_pERK (rb85).Tif]

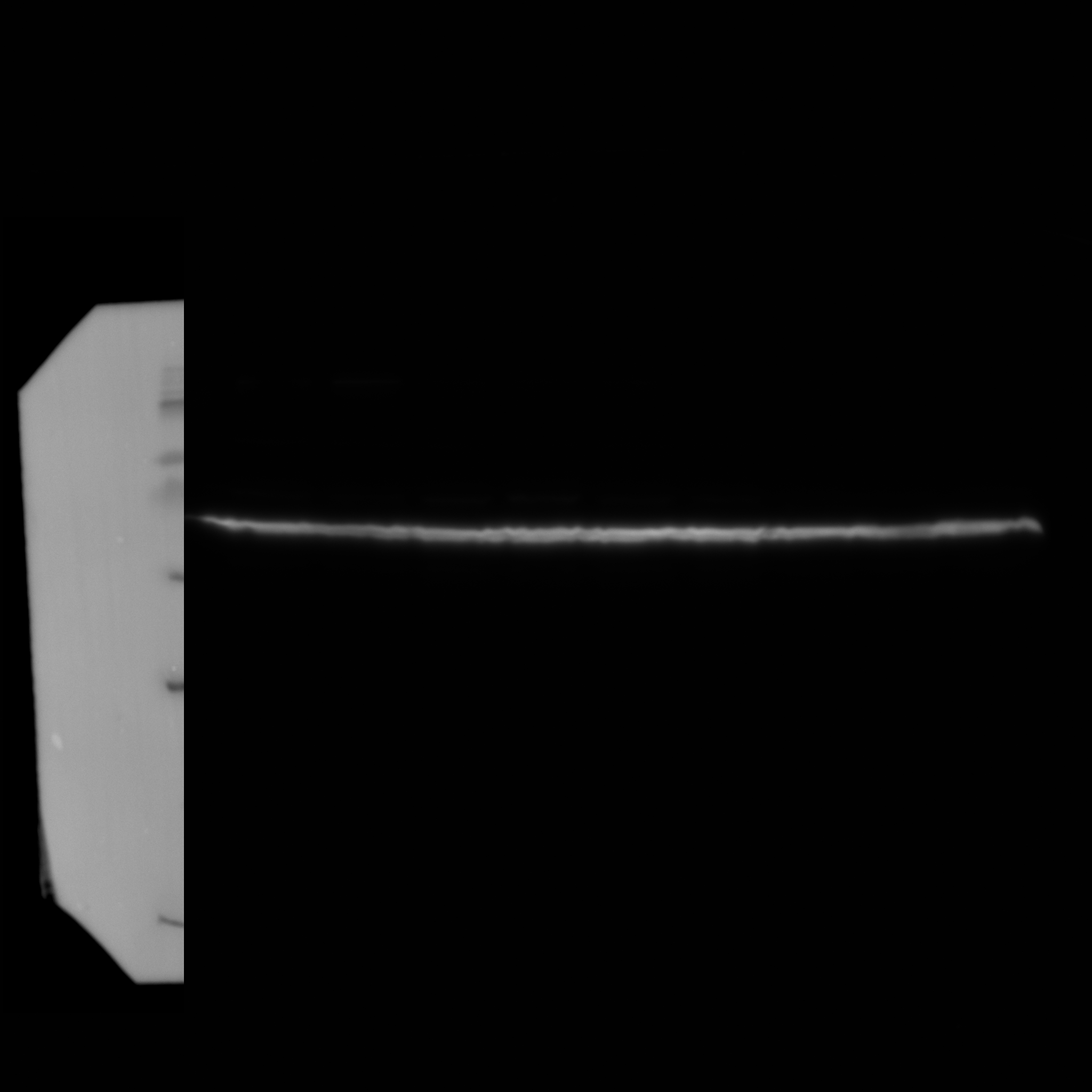

Supplement: Supplementary file 1 [file ijms-26-05476-s001.zip › Supplementary materials S1 Unprocessed immunoblots/Fig.4_ERK (FR180204)/not shown_241221_2_blot2_AK13-1_0%FCS_FR-204_time_v.1.1_VDAC1 (rb201).Tif]

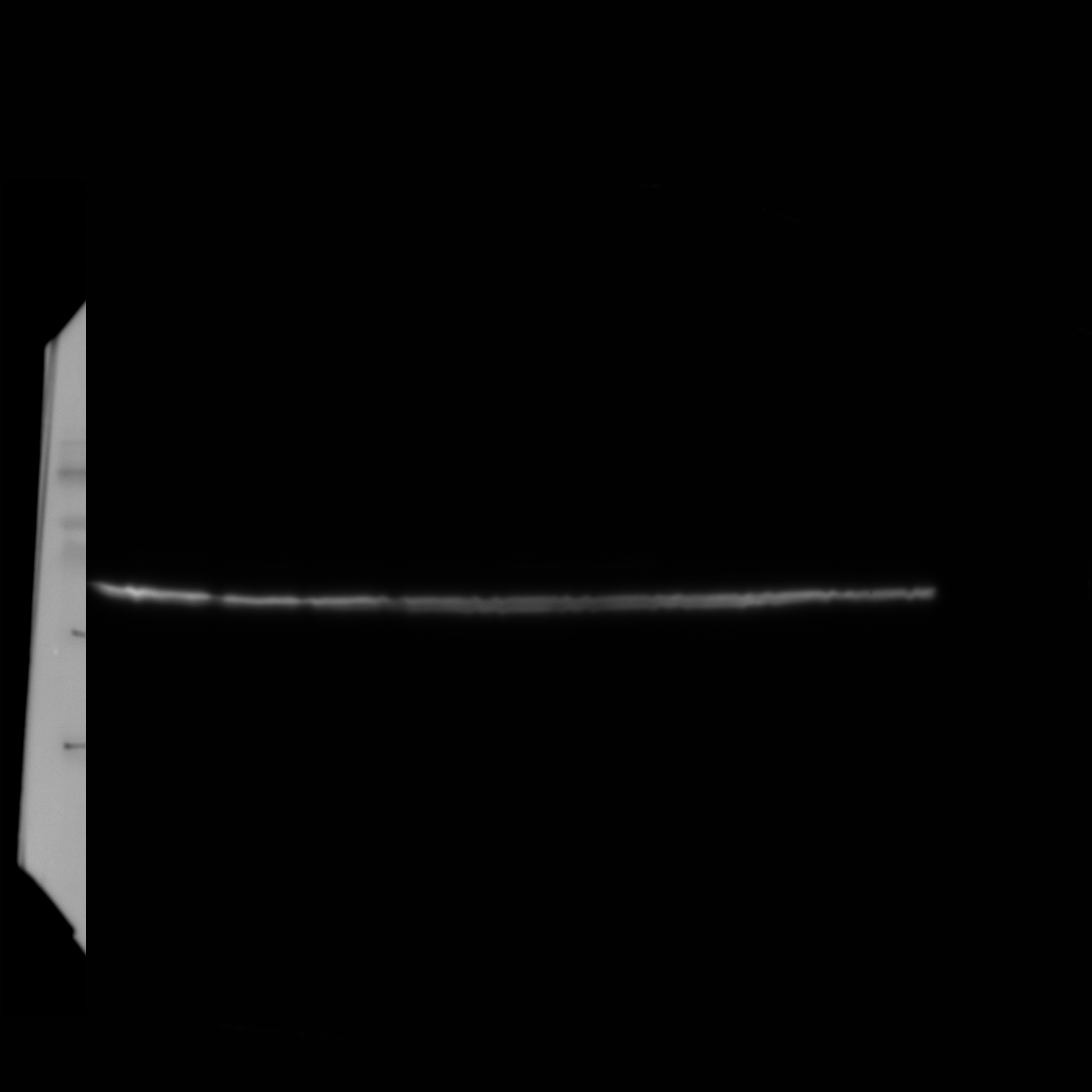

Supplement: Supplementary file 1 [file ijms-26-05476-s001.zip › Supplementary materials S1 Unprocessed immunoblots/Fig.4_ERK (FR180204)/not shown_241221_2_blot3_AK13-1_0%FCS_FR-204_time_v.1.2_VDAC1 (rb201).Tif]

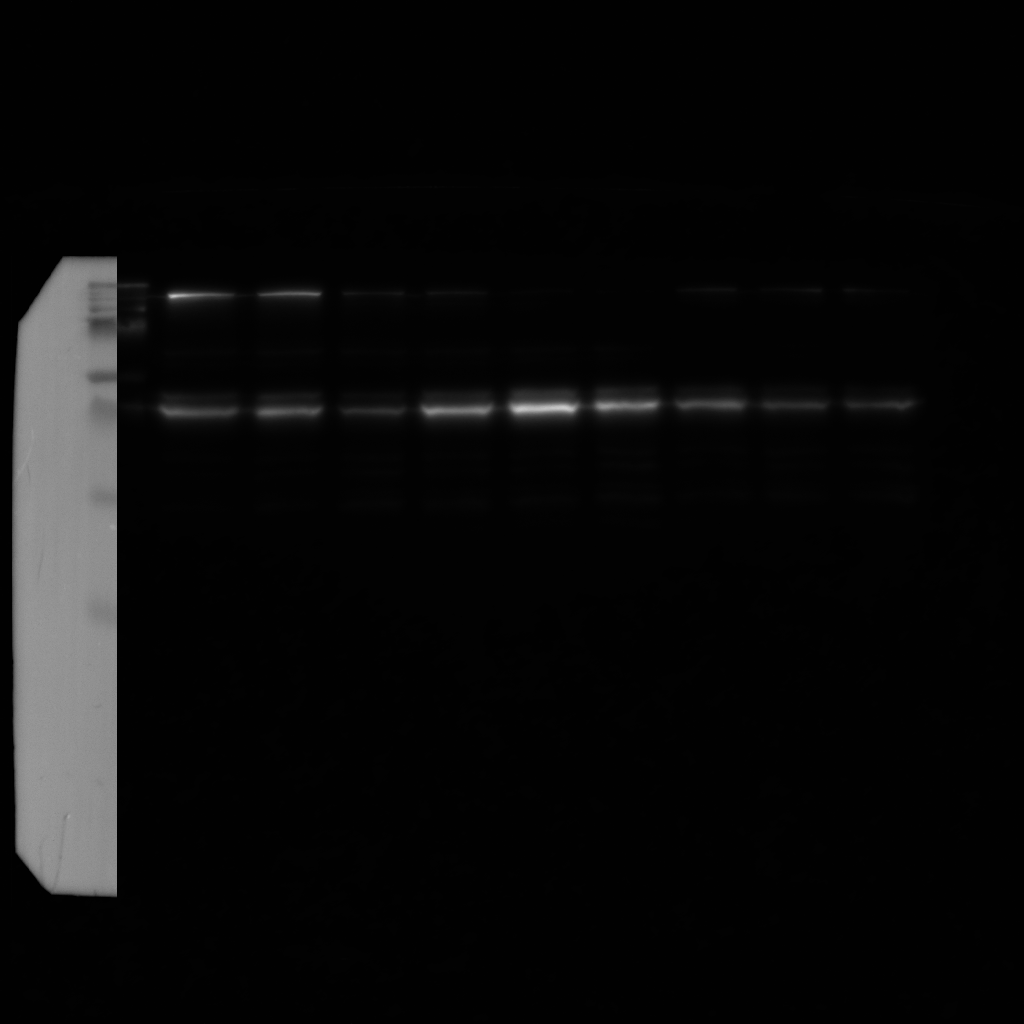

Supplement: Supplementary file 1 [file ijms-26-05476-s001.zip › Supplementary materials S1 Unprocessed immunoblots/Fig.4_ERK (SCH772984)/Figure 4A_241221_1_blot3_AK13-1_SHC-984_time_1.2_pERK (rb85).Tif]

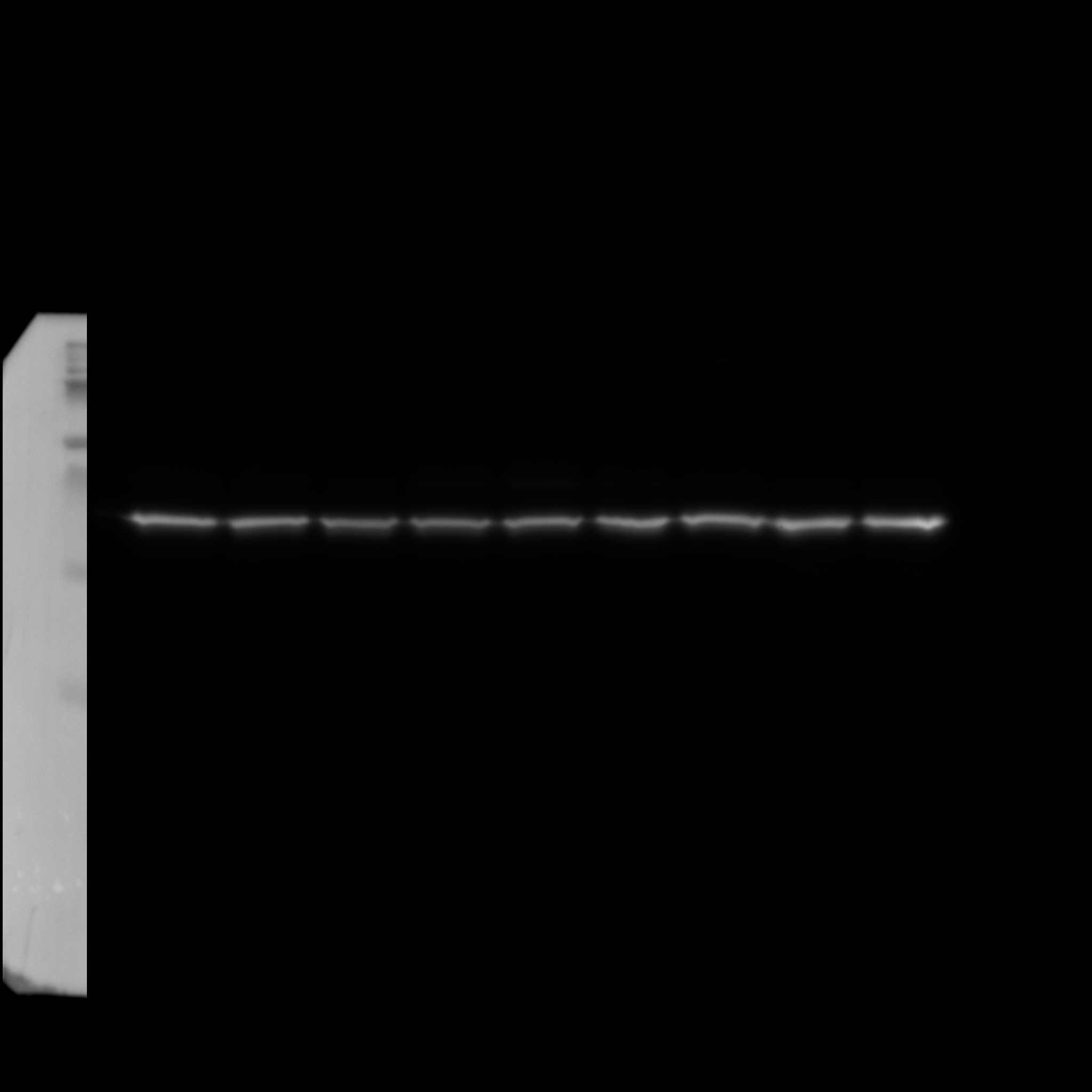

Supplement: Supplementary file 1 [file ijms-26-05476-s001.zip › Supplementary materials S1 Unprocessed immunoblots/Fig.4_ERK (SCH772984)/Figure 4A_241221_2_blot3_AK13-1_SHC-984_time_1.2_VDAC1 (rb201).Tif]

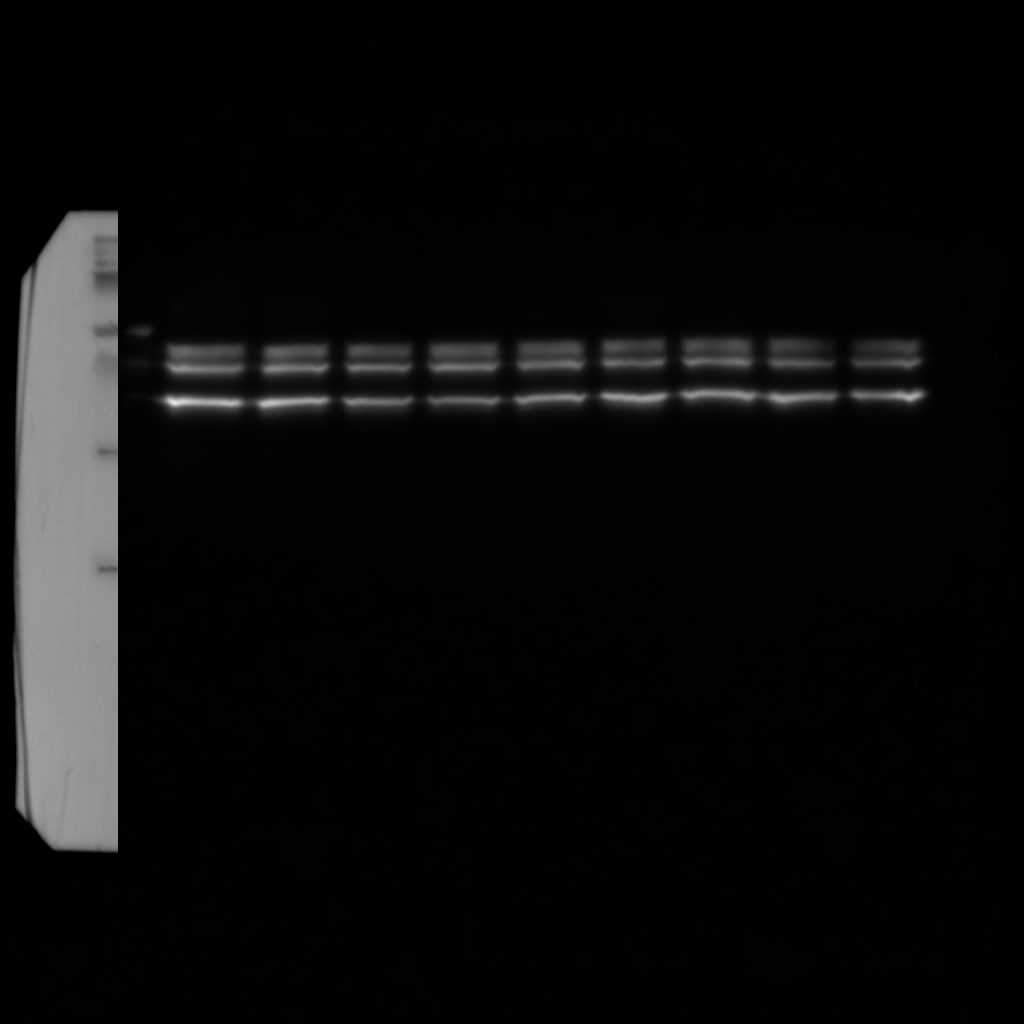

Supplement: Supplementary file 1 [file ijms-26-05476-s001.zip › Supplementary materials S1 Unprocessed immunoblots/Fig.4_ERK (SCH772984)/Figure 4A_241221_3_blot3_AK13-1_SHC-984_time_1.2_ERK (rb81).Tif]

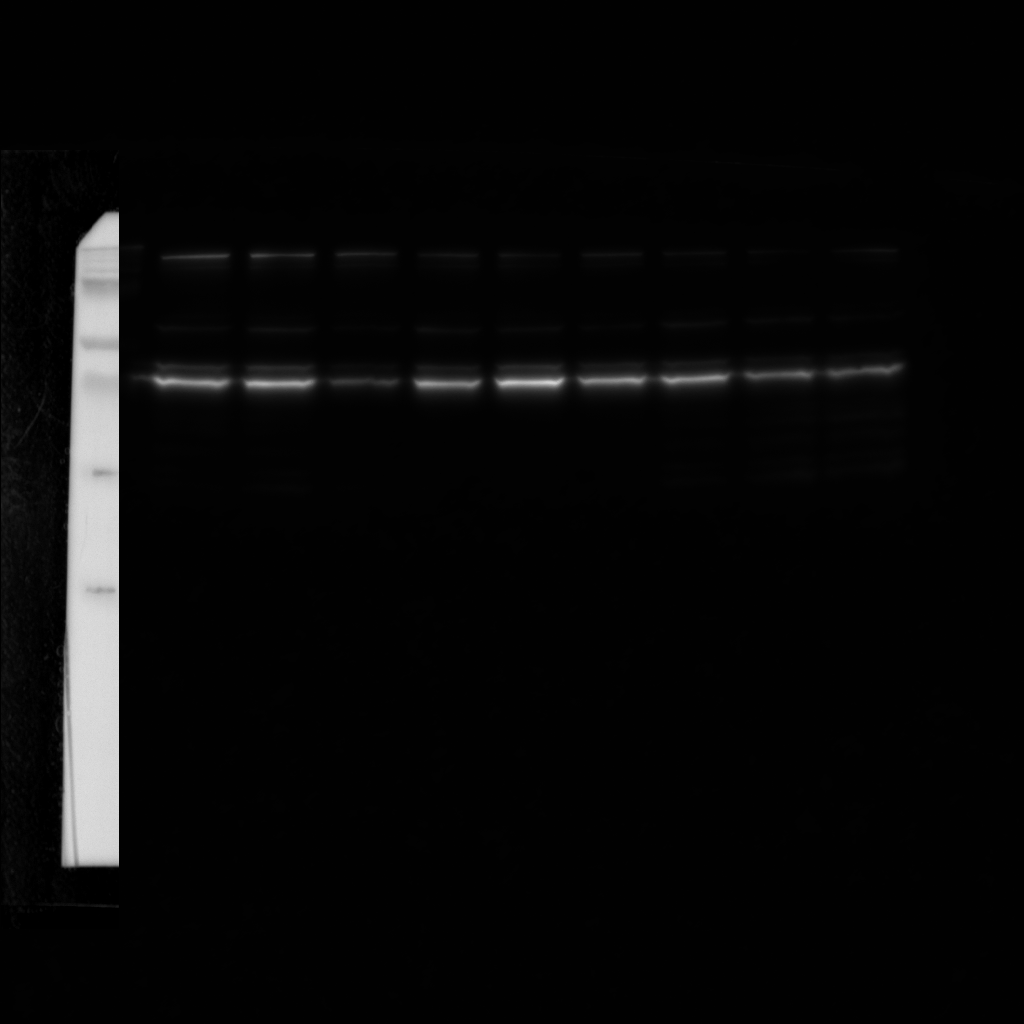

Supplement: Supplementary file 1 [file ijms-26-05476-s001.zip › Supplementary materials S1 Unprocessed immunoblots/Fig.4_ERK (SCH772984)/not shown_241221_1_blot1_AK13-1_SHC-984_time_1.0_pERK (rb85).Tif]

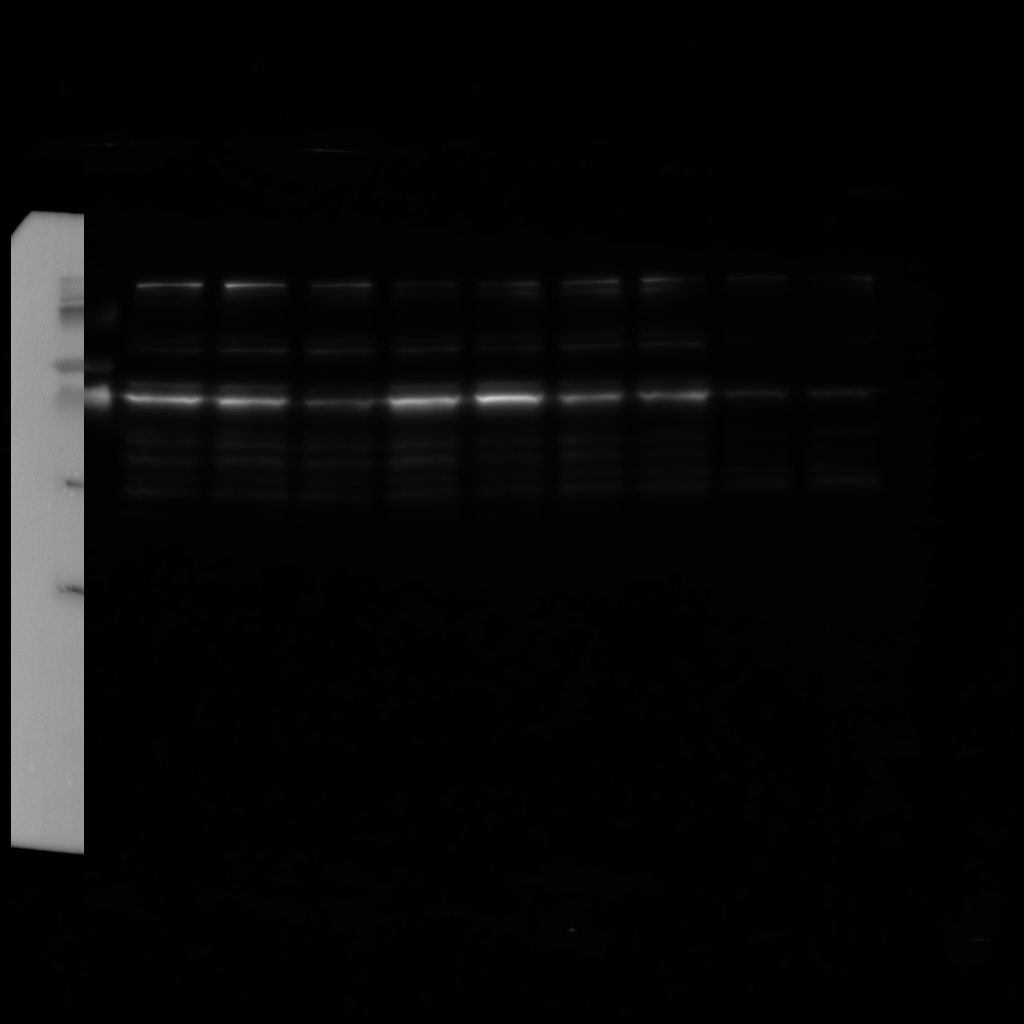

Supplement: Supplementary file 1 [file ijms-26-05476-s001.zip › Supplementary materials S1 Unprocessed immunoblots/Fig.4_ERK (SCH772984)/not shown_241221_1_blot2_AK13-1_SHC-984_time_1.1_pERK (rb85).Tif]

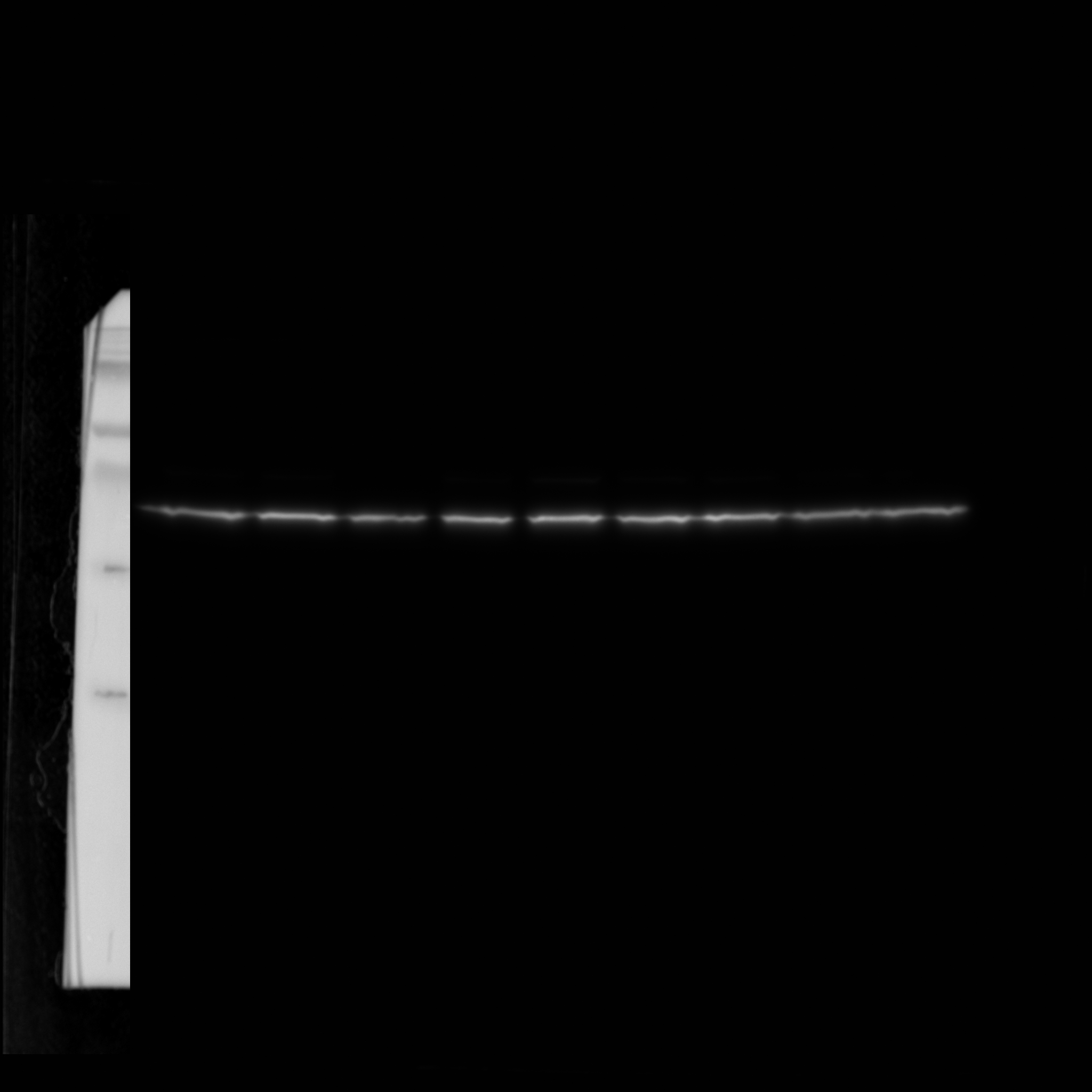

Supplement: Supplementary file 1 [file ijms-26-05476-s001.zip › Supplementary materials S1 Unprocessed immunoblots/Fig.4_ERK (SCH772984)/not shown_241221_2_blot1_AK13-1_SHC-984_time_1.0_VDAC1 (rb201).Tif]

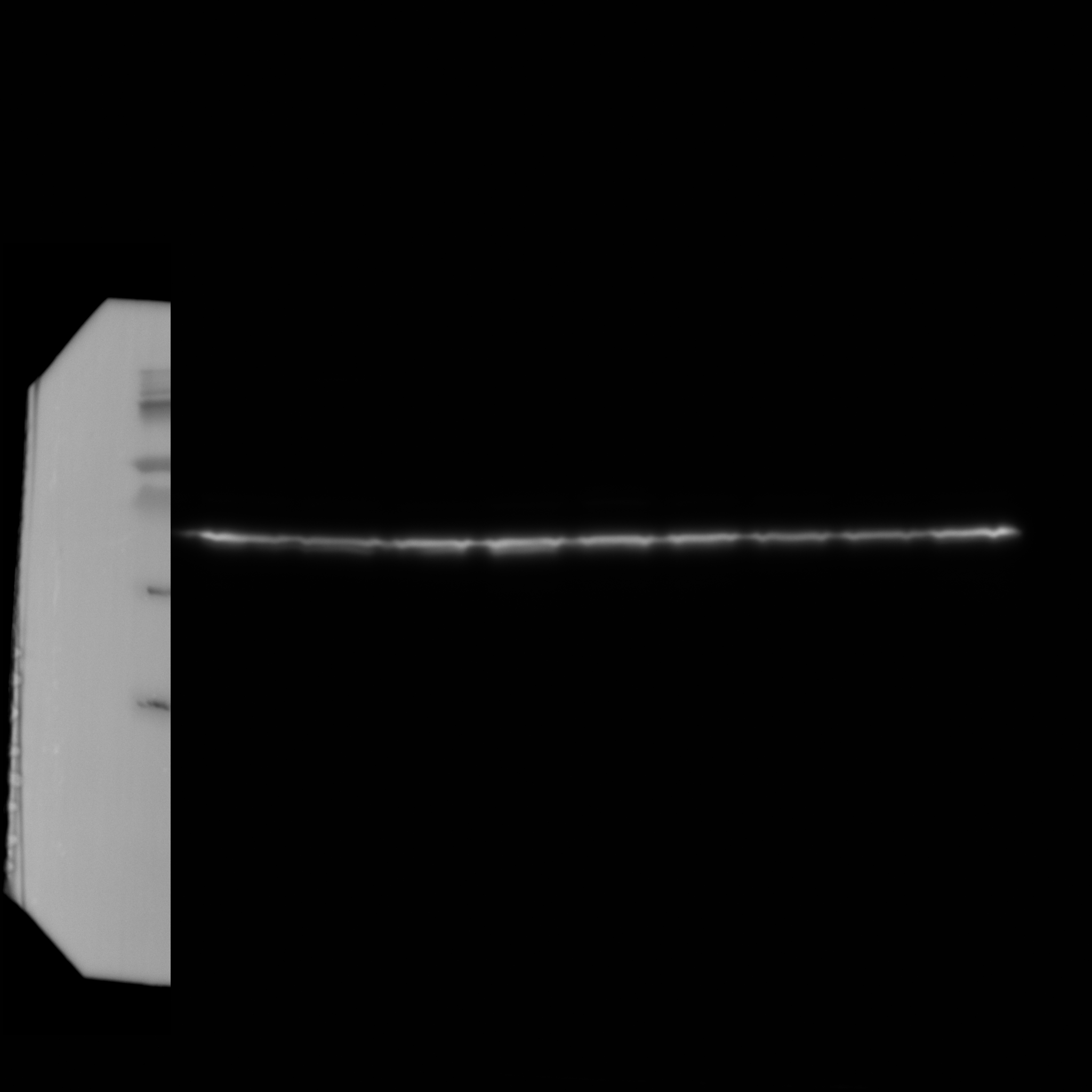

Supplement: Supplementary file 1 [file ijms-26-05476-s001.zip › Supplementary materials S1 Unprocessed immunoblots/Fig.4_ERK (SCH772984)/not shown_241221_2_blot2_AK13-1_SHC-984_time_1.1_VDAC1 (rb201).Tif]

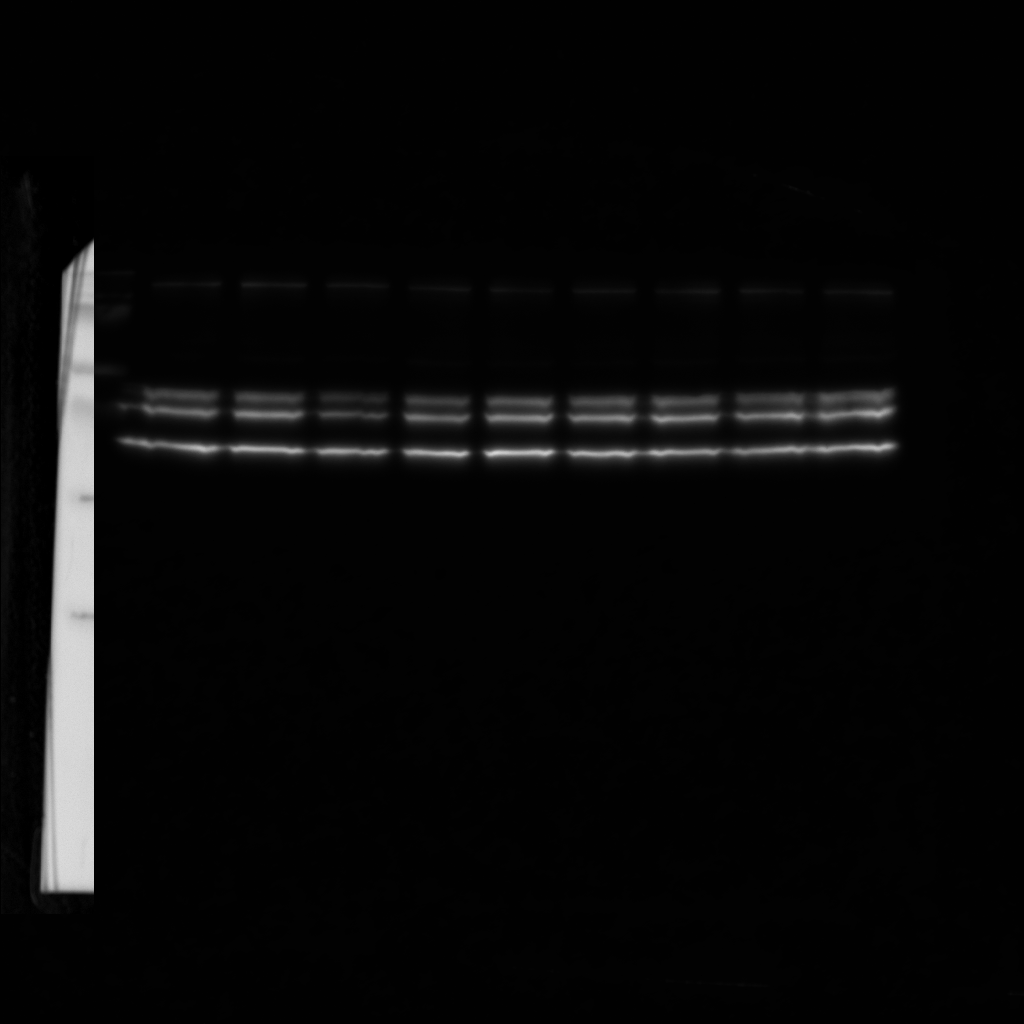

Supplement: Supplementary file 1 [file ijms-26-05476-s001.zip › Supplementary materials S1 Unprocessed immunoblots/Fig.4_ERK (SCH772984)/not shown_241221_3_blot1_AK13-1_SHC-984_time_1.0_ERK (rb81).Tif]

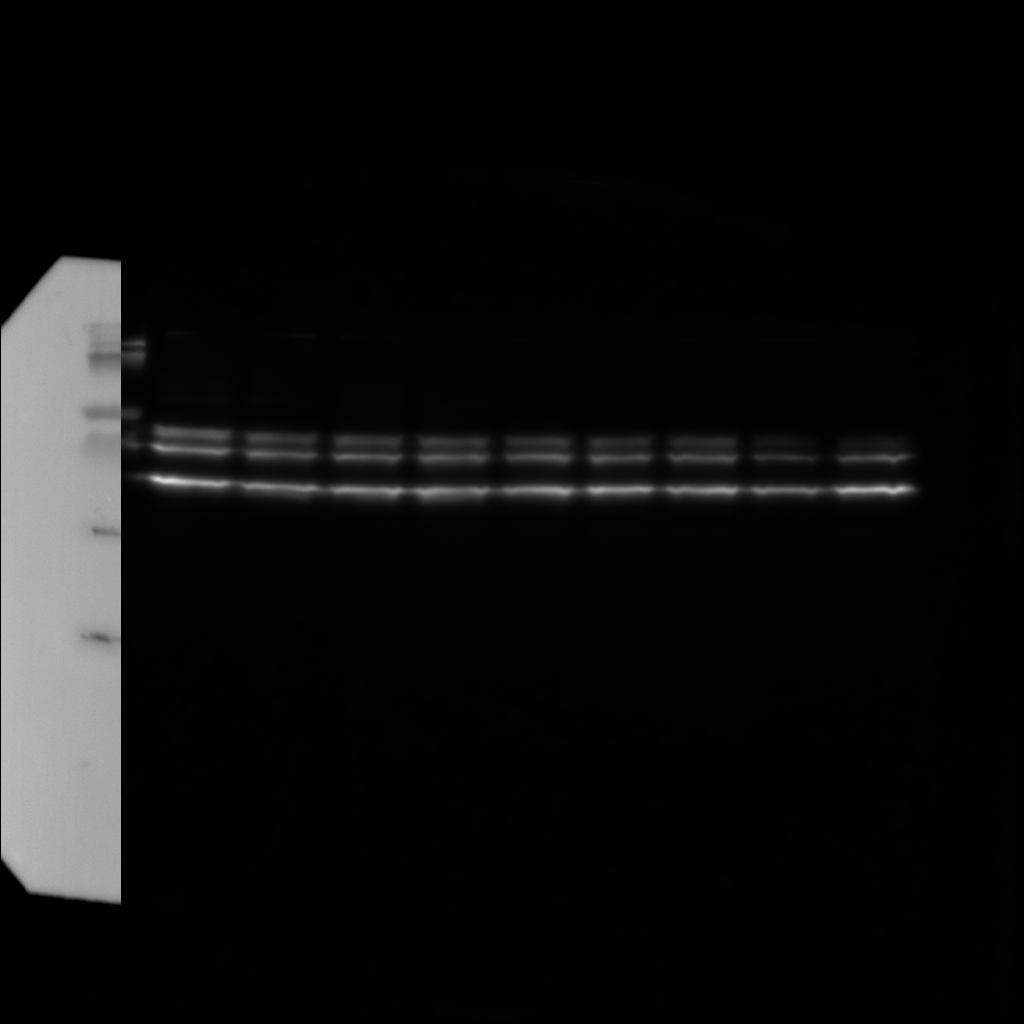

Supplement: Supplementary file 1 [file ijms-26-05476-s001.zip › Supplementary materials S1 Unprocessed immunoblots/Fig.4_ERK (SCH772984)/not shown_241221_3_blot2_AK13-1_SHC-984_time_1.1_ERK (rb81).Tif]

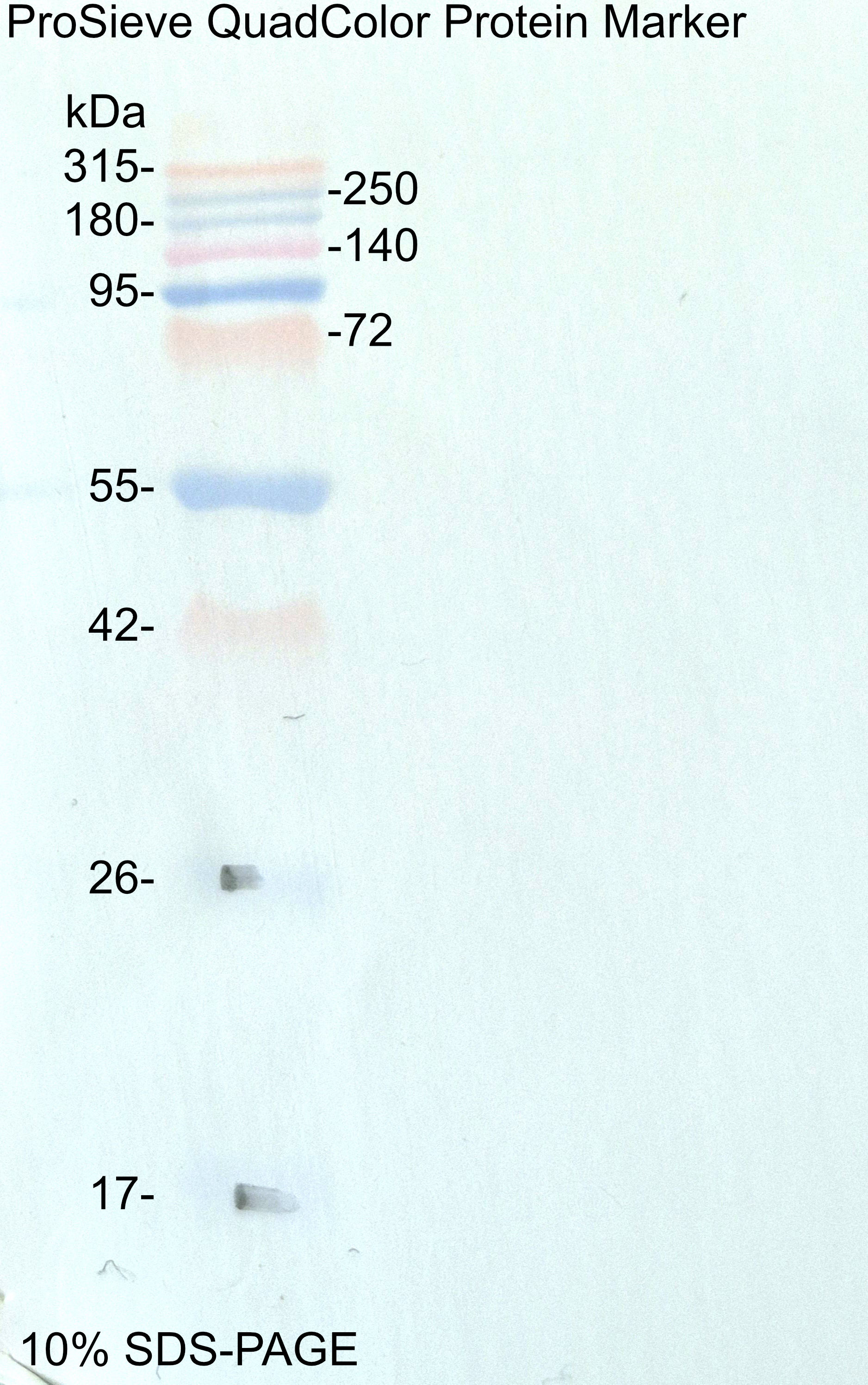

Supplement: Supplementary file 1 [file ijms-26-05476-s001.zip › Supplementary materials S1 Unprocessed immunoblots/ProSieve QuadColor Protein Marker.jpg]
